# Systems Analysis of Lactose Metabolism in *Trichoderma reesei* Identifies a Lactose Permease That Is Essential for Cellulase Induction

**DOI:** 10.1371/journal.pone.0062631

**Published:** 2013-05-08

**Authors:** Christa Ivanova, Jenny A. Bååth, Bernhard Seiboth, Christian P. Kubicek

**Affiliations:** 1 Institute of Chemical Engineering, University of Technology of Vienna, Vienna, Austria; 2 Lund University, Lund, Sweden; 3 Austrian Institute of Industrial Biotechnology (ACIB) GmBH c/o Institute of Chemical Engineering, University of Technology of Vienna, Vienna, Austria; Technical University of Denmark, Denmark

## Abstract

*Trichoderma reesei* colonizes predecayed wood in nature and metabolizes cellulose and hemicellulose from the plant biomass. The respective enzymes are industrially produced for application in the biofuel and biorefinery industry. However, these enzymes are also induced in the presence of lactose (1,4-*0*-*ß*-d-galactopyranosyl-d-glucose), a waste product from cheese manufacture or whey processing industries. In fact, lactose is the only soluble carbon source that induces these enzymes in *T. reesei* on an industrial level but the reason for this unique phenomenon is not understood. To answer this question, we used systems analysis of the *T. reesei* transcriptome during utilization of lactose. We found that the respective CAZome encoded all glycosyl hydrolases necessary for cellulose degradation and particularly for the attack of monocotyledon xyloglucan, from which *ß*-galactosides could be released that may act as the inducers of *T. reesei’s* cellulases and hemicellulases. In addition, lactose also induces a high number of putative transporters of the major facilitator superfamily. Deletion of fourteen of them identified one gene that is essential for lactose utilization and lactose uptake, and for cellulase induction by lactose (but not sophorose) in pregrown mycelia of *T. reesei*. These data shed new light on the mechanism by which *T. reesei* metabolizes lactose and offers strategies for its improvement. They also illuminate the key role of ß-D-galactosides in habitat specificity of this fungus.

## Introduction

The β-(1,4)-linked glucose polymer cellulose and hemicellulose polysaccharides of varying composition make up 60–80% of the plant cell wall and arise from the utilization of solar energy and carbon dioxide by plants at an annual production rate of about 7.2 and 6×10^10^ tons, respectively [1]. These polymers therefore represent the global reservoir of renewable carbon for the production of biofuels and platform chemicals used in biorefineries.

The fungus *Trichoderma reesei* (the anamorph of the tropical ascomycete *Hypocrea jecorina*) serves as the major producer organism for the enzymes needed to degrade the above mentioned polymers to soluble monosaccharides [2]. Since most of these enzymes are formed only adaptively, the cultivations must be performed in the presence of an inducer which is, in most cases, a cellulose and hemicellulose containing waste material. Interestingly, lactose (1,4-*0*-*ß*-d-galactopyranosyl-d-glucose), which is produced to around 1.2 million tons per annum worldwide as a by-product from cheese manufacture or from whey processing industries, also induces cellulase formation in *T. reesei* (but not in other fungi) and is the only economic soluble carbon source for this purpose [3]. On the other side, lactose metabolism is slow and leads to lower cellulase yields than on cellulose, thus warranting a deeper understanding of lactose metabolism towards its targeted improvement [4], [5].

In this study, we exploit functional genomics resources to perform a systems analysis of the *T. reesei* transcriptome during utilization of lactose and formation of cellulases and hemicellulases. We have produced strains containing deletions in genes encoding proteins identified from the transcriptome datasets and evaluated them for their ability to use lactose and produce cellulases. From this analysis, we identified a protein whose deletion completely impairs lactose utilization and cellulase gene expression in *T. reesei*. These data shed new light on the mechanism by which lactose induces cellulase formation in *T. reesei*, and illuminate the key role of ß-D-galactosides in habitat specificity of this fungus.

## Results

### The Lactose-regulated Transcriptome of *T. reesei*

In order to identify genes that are induced or repressed by lactose, we determined the transcriptional profiles of *T. reesei* QM 9414 during the initial growth phase (when 25–30% of the carbon source have been consumed) on lactose, glucose and glycerol, and monitored those that were >2-fold different at a p<0.05 between lactose and either of the two other sugars. In order to remove those genes that would be specific to either glucose or glycerol, but not lactose, we then selected those genes that were present in both comparisons. These comprised a total of 1540 genes ( = 16.8% of the total genome), of which 899 (58.4%) and 641 (41.6%) were up- and downregulated, respectively. Of these 1540 genes, 523 up- and 367 downregulated genes could either be identified or at least attributed to a function (Table 1; Table S1). It was found that 376 and 274 genes, respectively, encoded unknown or orphan, lineage-specific proteins. Also, 127 (14.1%) and 59 (9.2%) of the up- and downregulated genes encoded genes containing an N-terminal signal sequence and therefore putatively comprise secreted and/or membrane proteins.

**Table 1:** Balance of significantly* regulated transcripts during growth of *T. reesei* on lactose.

	Upregulated				Downregulated			
	all genes		secreted		all genes		secreted	
	Number	%*	Number	%**	Number	%*	Number	%**
Identified	523	58.1	88	16.8	367	57.2	25	6.8
Unknown	298	33.1	28	9.3	247	38.5	25	10.1
Unique	78	8.8	11	14.1	27	4.3	9	33.3
Total	899		127	14.1	641		59	9.2

* either>or <2-fold vs. glucose and glycerol.

* %-values refer to the total number of genes.

** %-values refer to the category of genes (e.g. identified, unknown or unique, resp.).

We employed two strategies to identify genes that are overrepresented in the lactose-specific transcriptome (see Materials and Methods): after grouping genes according to FunCat categories, we first calculated the ratio of the number of up- vs downregulated genes belonging to each category and tested which of them was beyond the average (p<0.05). In addition, we expressed the number of transcripts that belong to individual Funcat groups as the percentage of all genes in the >2-fold transcriptome, and divided this value by the percentage of the total number of genes of this Funcat group in the 9143 genes of the *T. reesei* genome. These “enrichment” values were then again tested for those lying beyond the mean variation (p<0.05). Genes involved in solute transport (of the major facilitator superfamily; “MFS permeases” and of amino acids), and “CAZymes” ( = enzymes acting on extracellular polysaccharides) were enriched in lactose-grown cultures, and genes required for proteasome and mitochondrial function were strongly downregulated (Table 2). In addition, genes that only passed the first criterion were genes encoding GH-auxiliary proteins, G-protein coupled receptors and transcription factors (which all contained more up- than downregulated members), and genes for ribosomal functions (which were mostly downregulated).

**Table 2:** Major changes in the transcriptome during cultivation of *T. reesei* on lactose.

				regulation			T	G	genome %	fold (up/down)
FunCat	function	metabolic function	up		down		1540	9143	16.8	1.49
				*up*		*down*				
1	metabolism		156		120		276	2074	13.3	1.3
98		Oxidation		29		30				
98		Short chain dehydrogenases/reductases		15		12				
01_05		carbohydrate metabolism		14		12				
98		metal ion metabolism		7		6				
01_01		amino acid metabolism		27		21				
01_06		lipid metabolism		12		6				
01_20		secondary metabolism		2		5				
ND		others		50		28				
01_25_01	glycosyl hydrolases		59		10		69	201	**34.3**	**5.9**
01_25	GH auxiliary proteins		8		1		9	35	25.7	**8**
01_25_03	proteases		17		7		24	82	29.2	2.42
4_16	mitochondrial function		3		34		37	74	**50**	**0.088**
11_02_03_04	transcription factors		42		10		52	351	14.8	**4.2**
12_4	translation		1		12		13	88	14.7	**0.083**
14_13	proteosomal function		0		12		12	27	**44.4**	N*
30_05_02_24	GPCR		5		1		6	21	28.5	**5**
20_03	major facilitator superfamily		111		36		147	260	**56.5**	**3.1**
99	orphan protein		78		32		110	506	21.7	2.4
99	unknown proteins		298		245		543	3530	15.4	1.21
	**Total**		**622**		**400**					

Data on grey background were statistically significant (p<0.05). *N, not calculated because of zero in one condition.

### The Lactose-induced CAZome

As noted above, and consistent with the hypothesis of this work, genes encoding glycosyl hydrolases and auxiliary enzymes or proteins for them comprised a major group of genes (sixty-three) in the lactose-upregulated transcriptome (Table S1). Among them, we identified both cellobiohydrolases (CEL7A and CEL6A), all endoglucanases (CEL5A, CEL7B, CEL12A, CEL45A), and six *ß*-glucosidases (Table 3). Also four xylanases (XYN2, XYN3, XYN4 and XYN6), two *ß*-xylosidases, six *α*-galactosidases, two *α*-fucosidases, two *α*-arabinofuranosidases, two methyl-*α*-glucuronidase, as well as one xyloglucanase, one endo-*ß*-1,3/1,4-glucanases, and one pectinase were present. Finally, three acetyl-xylan esterases, two GH61 cellulose monooxygenases [6], one swollenin (a protein containing an expansin and a cellulose binding domain [7] and CIP1 (a protein containing a cellulose-binding domain [8]); were found, thus completing the above enzymes to a full cellulolytic and hemicellulolytic enzyme spectrum.

**Table 3:** The lactose-induced CAZome of *T. reesei.*

Protein ID	Lact vs Glc	Lact vs Gly	p-value	annotation
22197	4.052	9.923	0.00338	GH1 ß-glucosidase
120749	21.174	116.676	0.00635	GH1 ß-glucosidase
120229	8.373	8.239	0.00323	GH10 xylanase
123818	28.587	25.835	0.00273	GH11 xylanase XYN2
123232	72.563	73.564	0.000887	GH12 endo-ß-1,4-glucanase
108477	5.864	3.143	0.0104	GH13 α-glucosidase
55886	4.553	3.907	0.0369	GH16 endo-ß-1,3/1,4-glucanase
65162	7.393	2.217	0.00458	GH18 chitinase
77299	6.826	4.682	0.0121	GH2 exo-ß-D-glucosaminidase
57857	5.956	4.869	0.0039	GH2 ß-mannosidase
69245	101.158	38.535	0.00396	GH2 ß-mannosidase
103458	2.346	2.378	0.00439	GH25 lysozyme
72632	76.354	48.322	0.00442	GH27 α-galactosidase
72704	7.091	2.103	0.00501	GH27 α-galactosidase
65986	35.526	21.134	0.00119	GH27 α-galactosidase
27259	3.285	3.374	0.0194	GH27 α-galactosidase
59391	23.278	25.785	0.00291	GH27 α-galactosidase
55999	5.679	4.295	0.0069	GH27 α-galactosidase
82227	8.106	8.403	0.0014	GH3 ß-glucosidase
46816	5.255	6.969	0.0106	GH3 ß-glucosidase
108671	2.69	2.545	0.0221	GH3 ß-glucosidase
76672	2.108	2.121	0.0421	GH3 ß-glucosidase
121127	107.07	91.618	0.0011	GH3 ß-xylosidase
111849	16.879	12.353	0.0035	GH30 glucuronoyl-xylanase
69276	4.691	3.5	0.00897	GH30 glucuroyl xylanase XYN6
60085	3.819	11.31	0.00989	GH31 α-glucosidase
69944	3.73	4.186	0.0167	GH31 α-xylosidase
64827	2.979	2.612	0.00495	GH36 α-galactosidase
124016	12.7	5.756	0.00915	GH36 α-galactosidase
123226	35.271	14.146	0.00183	GH37 trehalase
3739	233.319	63.254	0.00112	GH43 ß-xylosidase/α-L-arabinofuranosidase
49976	12.862	24.602	0.00443	GH45 endoglucanase V; C-terminal CBM1 module
45717	11.069	8.48	0.00302	GH47 α-1,2-mannosidase
120312	163.479	152.15	0.00132	GH5 endoglucanase Cel5A
81087	62.912	76.828	0.00108	GH5 glycoside hydrolase
123283	4.169	5.102	0.00817	GH54 α-L-arabinofuranosidase
55319	3.309	5.048	0.00178	GH54 α-L-arabinofuranosidase; C-terminal CBM42 module
72567	273.081	274.233	0.00225	GH6 cellobiohydrolase II (Cel6A)
120961	62.728	62.882	0.00128	GH61 polysaccharide monooxygenase
73643	21.043	25.74	0.000785	GH61 polysaccharide monooxygenase
76210	10.795	11.667	0.00228	GH62 α-L-arabinofuranosidase
65137	3.654	6.227	0.0134	GH64 ß-1,3-glucanase
25224	5.701	8.362	0.00653	GH65 a,α-trehalase
123456	11.275	2.234	0.00412	GH65 a,α-trehalase
72526	275.106	218.78	0.00163	GH67 α-glucuronidase
123989	246.472	91.566	0.00151	GH7 cellobiohydrolase I
122081	3.3	3.913	0.0095	GH7 endoglucanase Cel7B
71532	2.649	7.183	0.00359	GH71 α-1,3-glucanase
49081	5.419	3.008	0.0137	GH74 xyloglucanase; C-terminal CBM1 module
42152	2.83	6.149	0.0115	GH75 chitosanase
27395	3.292	2.494	0.0144	GH76 α-1,6-mannanase
55802	2.158	3.146	0.0206	GH76 α-1,6-mannanase
122495	19.358	20.326	0.00113	GH76 α-1,6-mannanase
106575	5.015	3.634	0.0091	GH79 methyl-ß-glucuronidase
79921	7.183	5.67	0.00323	GH92 α-1,2-mannosidase
74198	40.21	20.923	0.00652	GH92 α-1,2-mannosidase
5807	33.976	37.228	0.00346	GH95 α-L-fucosidase
58802	15.405	21.841	0.00663	GH95 α-L-fucosidase
123992	15.986	4.71	0.00527	swollenin
65215	12.104	2.749	0.00615	CE4 polysacharide deacetylase
54219	5.676	5.861	0.00149	CE5 acetyl xylan esterase
73632	5.339	7.325	0.00556	CE5 acetyl xylan esterase AXE1
73638	29.011	30.747	0.00204	CIP1

### Metabolic Characteristics of the Lactose Transcriptome

When the CAZyme encoding genes were not considered, 156 and 120 genes of the up- and downregulated transcriptome, respectively, comprised genes encoding enzymes known to be involved in metabolism. Of these genes, 106 and 92 could be attributed to specific metabolic pathways. Genes encoding enzymes for oxidative reactions and amino acid metabolism accounted for the highest number, but no significant differences were noted between the up- and downregulated genes.

Growth on lactose proceeds slower than on glucose and glycerol, and we therefore tested whether some of the genes specifically expressed on lactose could be subject to regulation by the relationship between growth rate and repression by the carbon catabolite repressor CRE1 as described by Portnoy et al. [9]. We have therefore investigated whether genes found by these authors would overlap with those upregulated on lactose. Indeed, we found 93 genes to be shared between these two conditions (Table S2). However, they were distributed between all nine groups found by Portnoy et al. [9], and also at about equal numbers. Consequently, none of these groups is particularly enriched and we therefore conclude that lactose does not create conditions for carbon catabolite repression or growth rate-dependent derepression.

Most of the transcription factors that were upregulated on lactose comprised members of the fungal-specific Zn(2)Cys(6) cluster family (58 genes), almost all of which have not yet been characterized in fungi. However, the genes encoding the general cellulase regulator XYR1 (Trire2:122208) and the recently described *N. crassa* cellulase-regulator CLR-2 (Trire2:60282; [10]) were found to be significantly upregulated on lactose (22- and 7-fold vs. glucose and 10 and 5-fold vs. glycerol, respectively).

Interestingly, eight genes that were significantly upregulated during growth on lactose comprised genes involved in sexual differentiation, including the mating type gene *mat1-2-1*, the *α*-type pheromone precursor *hpp1*, two pheromone receptors and genes involved in synthesis and processing of the pheromone precursor (Table 4).

**Table 4:** Genes involved in sexual development that are upregulated in *T. reesei* during growth on lactose.

Protein ID	lact vs glc*	lact vs glyc	p-value	annotation
62693	11.378	7.257	0.001	ABC-transporter Ste6p
34493	7.451	5.858	0.002	α-type peptide pheromone precursor *hpp1*
124222	2.898	4.37	0.010	CaaX-protease, related to *E. nidulans rce1*, involved in signal transduction
57526	15.982	16.307	0.003	GPCR, mating type pheromone G-protein coupled receptor
64018	12.801	6.654	0.003	GPCR, mating type pheromone G-protein coupled receptor
31134	7.111	5.816	0.002	isoprenylcysteine carboxyl methyltransferase
124341	18.462	4.564	0.003	mating protein MAT1-2-1
22093	3.354	4.094	0.004	Protein farnesyltransferase, alpha subunit

* fold change.

### The Lactose-induced MFS Permeases Comprise Members Involved in Transport of Hemicelluloses Monomers

**Figure 1:**
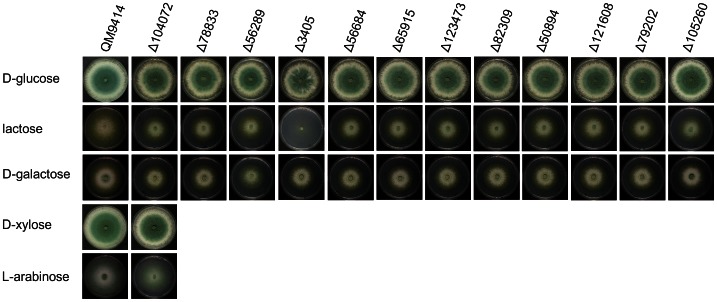
Growth of several MFS-knock out strains on D-glucose, lactose, D-galactose, and where appropriate, D-xylose, and L-arabinose. Pictures were taken 96 h after of incubation on minimal medium supplemented with the appropriate carbon source (1% w/v).

**Figure 2:**
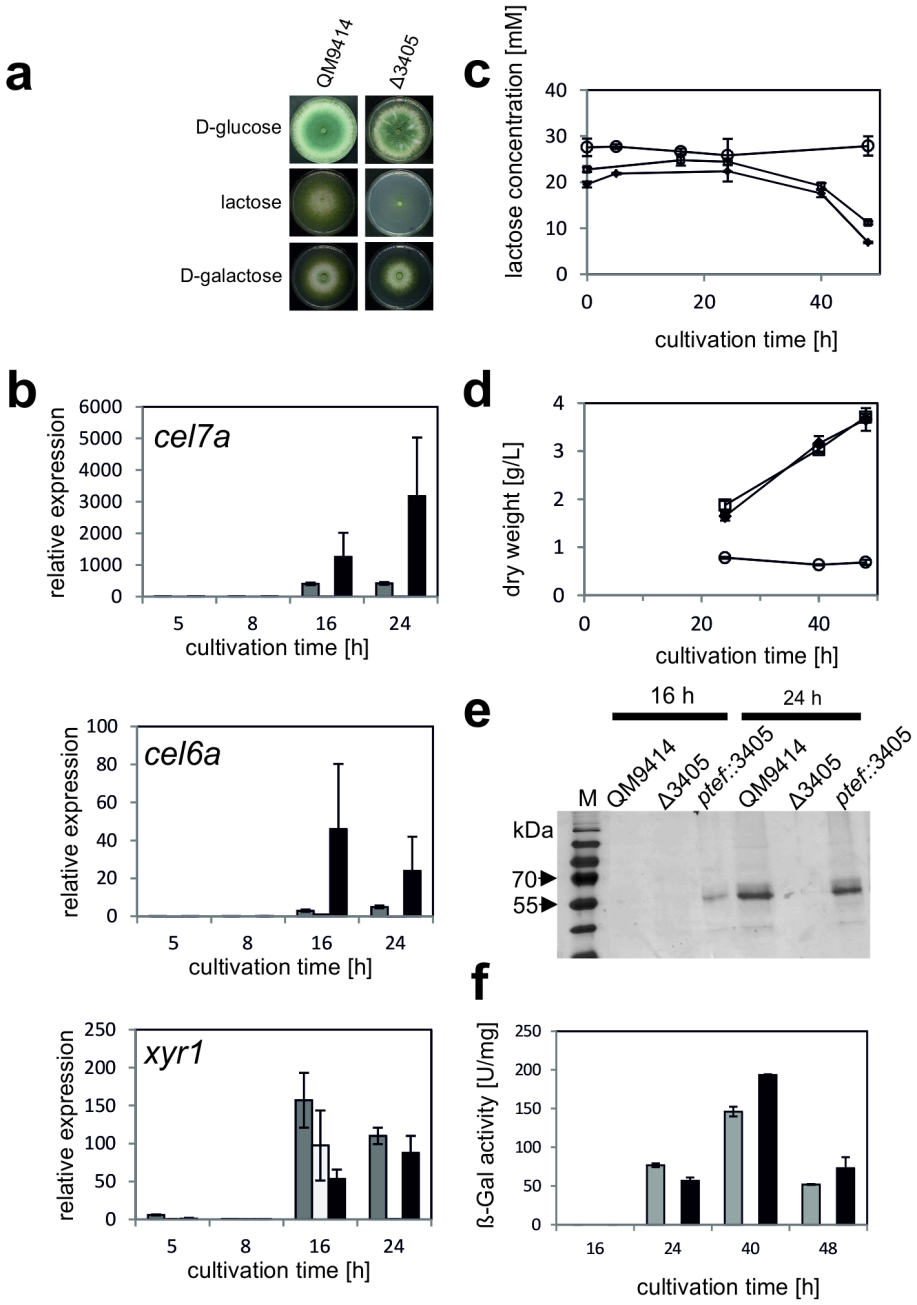
(a) Growth comparison of QM 9414 to the Δ3405 strain on minimal media supplemented with D-glucose, lactose and D-galactose. Pictures were taken on day 4. (b-g) Comparison of strains QM 9414 (grey), Δ3405 (white) and *ptef::3405* (black) pregrown in minimal medium supplemented with 1% glycerol and,after washing of the mycelium extensivly with sterile water, transferred to minimal medium containing 1% lactose. (b) Accumulation of *cel6a*, *cel7a* and *xyr1* transcripts measured by qPCR. Samples were taken 5, 8, 16 and 24 h after replacement to 1% lactose. The expression is given in relation to *tef1* gene expression, where the *tef1* expression value equals one. Mean values ± SD of three independent experiments are shown. (c) Lactose concentration in the medium during growth of QM 9414 (filled diamonds), Δ3405 (empty circles) and *ptef::3405* (empty squares) on 1% lactose. (d) Biomass formation of strains QM 9414 (filled diamonds), Δ3405 (empty circles) and *ptef::3405* (empty squares) on 1% lactose. Mean values ± SD of three independent experiments are given. (e) SDS-PAGE analysis of culture filtrates from QM 9414, Δ3405 and *ptef::3405.* Samples taken at 16 and 24 h after replacement to lactose. (f) ß-galactosidase activity determined with *o*-nitrophenyl-ß-D-galactopyranoside as the substrate. Error bars indicate the standard deviation of three independent experiments.

**Figure 3:**
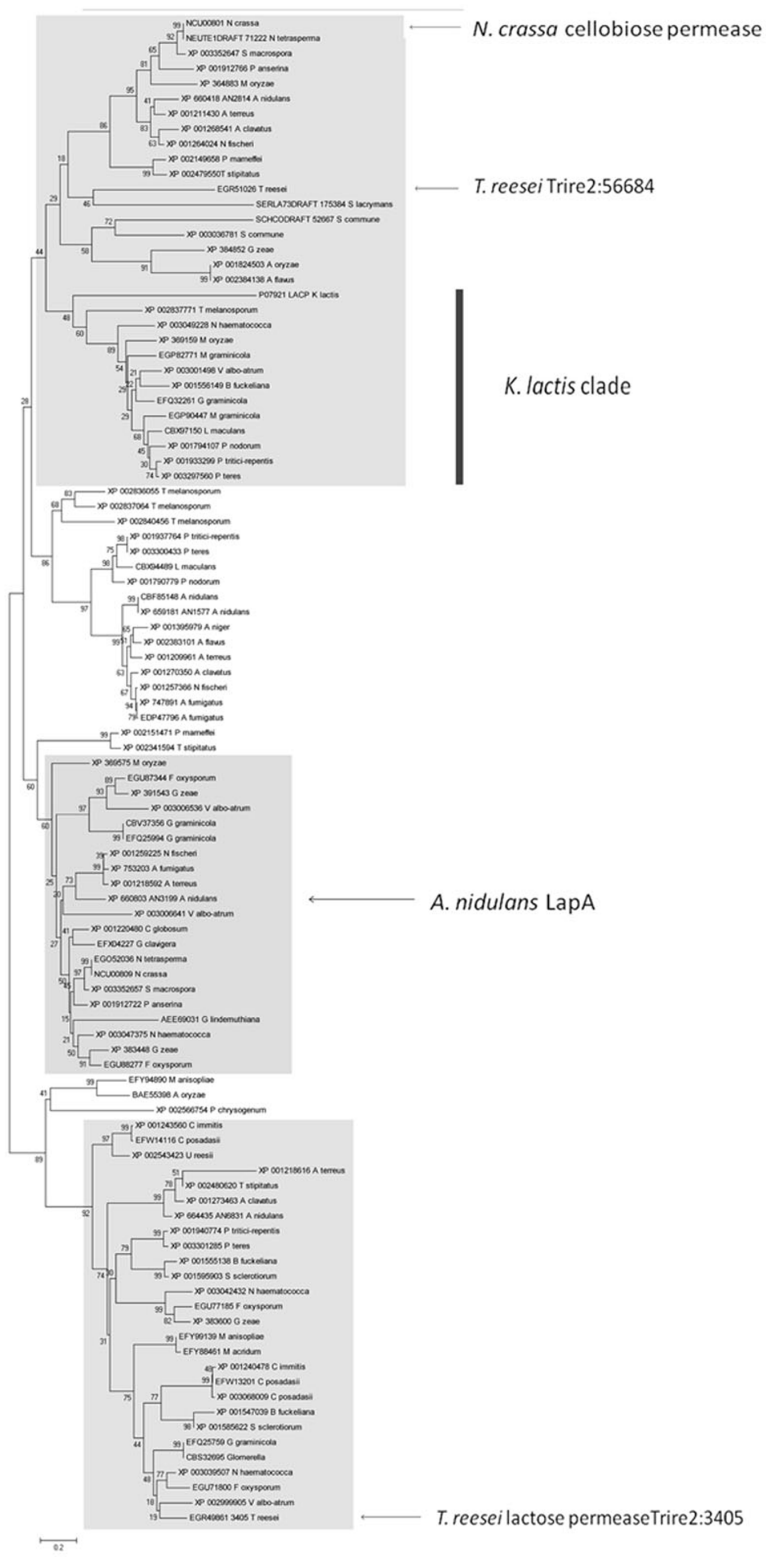
Phylogenetic analysis of the putative lactose permease Trire2:3405.

As noted above, MFS permeases comprised one of the largest gene groups upregulated on lactose (Table 2; Table S1). The function of most of them was unknown. However, BLAST analysis of their amino acid sequences resulted in putative mono- or disaccharide transporters of other fungi as next neighbours. One of them (Trire2:104072) has recently been described to enable *S. cerevisiae* to take up D-xylose [11]. To learn more about their function, we prepared deletion strains of the 14 most upregulated MFS permeases, and screened them for their ability to grow on glucose, lactose, D-galactose, and Trire2:104072 also on L-arabinose and D-xylose (Figure 1). The most severe effect was observed with Trire2:3405, whose deletion completely blocked the ability of the strain to grow on lactose containing medium, but not on D-glucose or D-galactose (Figure 2a). A phylogenetic analysis shows that Trire2:3405 is a member of a sister clade of a larger cluster containing the *K. lactis* and *A. nidulans* lactose permeases (Figure 3), and other so far uncharacterized orthologues from other Pezizomycota. All the other deletion mutants did not display any clear phenotype, and their identity therefore remains obscure. Interestingly, none of the mutants exhibited decreased growth on cellobiose, not even in the presence of nojirimycin (to inhibit the extracellular *ß*-glucosidases which could compensate the defect of a putative cellodextrin transporter; Figure S1). The expression of the MFS permeases was verified by qPCR analysis, which confirmed upregulation on lactose after 24 h of growth (Table S3).

### The Putative Lactose Permease is Essential for Cellulase Induction by Lactose

Having identified a putative lactose transporter of *T. reesei*, we were also interested whether this transporter would be relevant for cellulase production on lactose. To this end, we cultivated the deletion strain in Trire2:3405 on glycerol and then transferred it to lactose and recorded the formation of cellulases and the accumulation of cellulase transcripts (Figure 2 b–e). The Δ3405 strain did not grow after transfer to lactose, and also did not take it up; cellulase activities were reduced to zero and the levels of the *cel6A* and *cel7A* transcripts eliminated, as determined with qPCR. Placing Trire2:3405 under the strong and constitutive expression signals of the *tef1* promoter [12] resulted in an earlier appearance of the *cel6A* and *cel7A* transcripts (after 16 h of growth on 1% lactose) compared to the parental strain, and this finding was also reflected in the appearance of these two enzymes at 16 h in the culture filtrate but not in the parental strain, as shown by SDS-PAGE analysis (Figure 2e).

We also tested whether induction by sophorose, a strong inducer of cellulase formation in *T. reesei* (8), would also be affected by the knock out in the lactose transporter. However, pregrowth on glycerol and subsequent transfer to 1 mM sophorose revealed the same abundance of *cel7A* and *cel6A* transcripts in QM 9414 and Δ3405 after 4 and 6 hrs of incubation (data not shown). We therefore conclude that the lactose permease Trire2:3405 is specifically involved in cellulase induction by lactose.

Consistent with recent findings that induction of the cellulase regulator XYR1 does not require metabolism of D-galactose (14), expression of *xyr1* remained unaffected in the Δ3405 strain. Similarily, formation of the extracellular ß-galactosidase BGA1 was unaffected in strain 3405 (Figure 2 f).

## Discussion

In their natural habitat, fungi are unlikely to encounter the disaccharide lactose frequently as a carbon source. However, *T. reesei* is able to grow on it and simultaneously secretes cellulases. The present data offer some explanations for this enigma: besides a complete cellulase system, the upregulated CAZome also includes the cellulase accessory proteins CIP1, swollenin and two of the newly detected GH61 cellulose monooxygenases. Further, it comprises about half of the hemicellulases (xylanases, α- and ß-D-galactosidases, α-L-arabinofuranosidases and cellulose/hemicellulose deacetylases), three of four α-L-fucosidases and all enzymes acting on D-glucuronoyl-side chains (GH30 glucuronoyl xylanases, and GH67/GH79 α–D-glucuronidases). An indirect stimulation of expression of these genes (e.g. by a lower growth rate and/or relieve from carbon catabolite repression) appears unlikely when the respective transcriptomes displayed under these conditions are compared (*vide supra*).A plausible explanation therefore is that lactose mimics substance(s) present in the natural habitat of *T. reesei* (i.e. predegraded and decaying wood) that signal the availability of plant biomass tothe fungus. This physiological signal is likely another ß-D-galactoside, as some of them have been shown to be able to induce cellulases [13]. Such ß-D-galactooligosaccharides would typically occur in xyloglucans of the XXGG type [14], in which xylose residues can be substituted with α-1,2-L-fucopyranose-ß-1,2-D-galactopyranose and α-1,2-L-galactopyranose-ß-1,2-D-galactopyranose disaccharides and O-linked acetyl groups, side chains for which the respective hydrolytic enzymes are all induced in *T. reesei* by lactose. Further, the xyloglucans of dicotyledons are partially replaced by glucuronoarabinoxylan, which has a linear ß-1,4-linked D-xylopyranosyl backbone with glucuronosyl or 4-O-methyl glucuronosyl side chains, which are again structures for which *T. reesei* enzymes are induced. Xyloglucans are the hemicelluloses in the primary cell wall of dicotyledons and are strongly associated with cellulose by crosslinking cellulose microfibrils [15]. Our data leads to speculate that the xyloglucans are the initial target for *T. reesei* when feeding on plant biomass.

The Zn(2)Cys(6) transcriptional regulator XYR1 has been demonstrated to be responsible for the induction of cellulases, xylanases and also some of the α-L-arabinofuranosidases in *T. reesei* [12], [16], but it is not known whether this extends to all of the CAZyme genes upregulated on lactose. In this context, it is interesting that the orthologue of the recently described *N. crassa* cellulase regulator CLR-2 [10] was found to be upregulated by lactose. Since *in vivo* and *in vitro* studies have shown that only the binding sites for XYR1 and the HAP2/3/5 complex are in the *cel6A* promoter are occupied and are thus necessary for transcription of the cellulases in *T. reesei* [17], and a *xyr1* knock out is unable to induce cellulase gene transcription [16], it is unclear how CLR-2 could contribute to cellulase induction. It will be interesting to investigate whether it is responsible for the expression of some of the hemicellulase genes for which the regulator is as yet unknown.

Lactose also induces the genes for a number of oxidative enzymes such as a GMC oxidoreductase, multicopper oxidase, tyrosinase, and FAD-dependent oxidases and monoxygenases. The majority of these proteins were predicted to possess a signal peptide (by SignalP; p<0.05; [18]) and are thus putative secreted proteins. Their induction by lactose is enigmatic, because no oxidation products of lactose or D-galactose are present in the medium. On the other hand, these enzymes are considered to be involved in lignin degradation by white rot fungi [19], and aid in the attack of brown rot fungi on cellulose by Fenton chemistry [20]. We consider it possible that lactose, as a trigger for formation of the plant biomass decomposing machinery of *T. reesei*, also induces enzymes that can aid the action of the CAZymes by Fenton-type like reactions. Although the operation of Fenton-type chemistry in cellulose degradation has so far only been shown in brown-rot fungi, recent data in *N. crassa* also pointed in this direction [21]. The demonstration of Fenton-type chemistry in *T. reesei* would be an important step in further understanding the superiority of its secretome in cellulose hydrolysis.

The number of genes -1540 - that are significantly regulated during growth on lactose is higher than that recorded in previous studies on carbon catabolite repression or conidiation [9], [22], illustrating that growth on lactose involves a major physiological shift in *T. reesei*. While one would expect changes in genes associated with metabolism of lactose, our data show that other areas of metabolism (such as amino acid and lipid metabolism or metal ion uptake), mitochondrial functions and components of the proteasome are also altered. At this time, the physiological consequences of these changes are unknown; however they do not seem to be related to the different growth rate on lactose in comparison to glucose and glycerol, as only a few genes were shared between the present study and that by Portnoy et al. who used chemostat cultures at different growth rates [9]. Interestingly, secondary metabolism - which was reported to correlate with cellulase and hemicellulase gene upregulation [23] - was almost not affected by lactose: only two nonribosomal peptide synthases (Trire2:60751 and Trire2:67189) and none of the polyketide synthases at all were upregulated.

The lactose upregulated transcriptome also included a vast number of putative transporters of the major facilitator superfamily. The fact that gene deletion could only identify the function of one of them is likely due to a redundancy of their function. However, one of them turned out to be essential for lactose utilization. Interestingly, this transporter is not a member of the major fungal lactose permease clade identified recently ([24]; cf. Figure 3). The fact that its knock-out impairs triggering of cellulase gene expression by lactose also in resting cells, which do not require lactose for growth, illustrates that either the uptake or the intracellular presence and/or metabolism of lactose is essential for cellulase induction. These findings reject our previous assumption that lactose metabolism in *T. reesei* proceeds only *via* extracellular hydrolysis and subsequent uptake and metabolism of the monomers D-glucose and D-galactose [25], [26]. This theory was based on the findings that the genome inventory of *T. reesei* appears not to contain a gene encoding an intracellular ß-galactosidase. Intracellular ß-galactosidases belong to GH family 2. While the genome of *T. reesei* contains seven members of this group [6], and four have also been found in this study to be induced on lactose, a Blastp search identified all of them as ß-mannosidases (five), exo-ß-D-glucosaminidase (one) and ß-glucuronidase (one) and none as a ß-galactosidase. While we cannot rule out that one of them (e.g. the ß-glucuronidase) has also a ß-galactosidase activity, it is also possible that *T. reesei* has an intracellular ß-galactosidase that does not belonging to family 2. Identification of the respective enzyme would provide essential information for genetic manipulation of lactose metabolism and thus cellulase formation, and for the understanding of the utilization of this intriguing disaccharide in *T. reesei*.

## Materials and Methods

### Strains, Cultivations and Measurement of Growth, Lactose Consumption and Cellulase Formation

*T. reesei* QM9414 (ATCC 26921), an early cellulase producing mutant was used throughout this work and kept on potato dextrose agar (Sigma, St. Louis, MO) at 28°C. *T. reesei Δtku70* strain [27], an uridine auxotrophic strain lacking the *tku70* gene required for non-homologous end joining DNA-repair, was maintained on malt extract medium supplemented with uridine (10 mM).

Growth on different carbon sources was assessed on minimal medium containing (1 g/L MgSO_4_, 10 g/L KH_2_PO_4_, 6 g/L (NH_4_)_2_SO_4_, 3 g/L Na_3_citrate•2H_2_O, 1% glucose, 20 ml/L trace elements (5 mg/L FeSO_4_•7H_2_O, 1.6 mg/L MnSO_4_•H_2_O, 1. 4 mg/L ZnSO_4_•H_2_O and 2 mg/L CoCl_2_•2H_2_O) and 15 g/L agar) 1% (w/v) of the respective carbon source [28]. For cultivation in submerged cultures (shake flasks), strains were pregrown in 1% (w/v) glycerol for 24 h, mycelia were harvested by filtration and washed with sterile water, and equal amounts of mycelia then transferred to flasks containing the appropriate carbon source (1% w/v), and cultivated for up to 96 h.

Growth on solid medium was tested by inoculating agar plates with a small agar piece (5 mm diameter). The biomass in submerged culture was determined by filtering portions of culture onto Whatman no. 1 preweighted filter papers. The harvested biomass was dried at 80°C for 3 days, and then weighted.

Cellulase and ß-galactosidase activities were assayed as described previously [29] with *p*-nitrophenyl-*ß*-D-lactopyranoside and *p*-nitrophenyl-*ß*-D-galactopyranoside as the respective substrates. Lactose concentration in the medium was determined with the 3,5-dinitrosalicylic acid [30].

SDS-PAGE was performed as described by Ausubel et al. [31] using 10% polyacrylamide gels. To this end, the proteins from the culture supernatant were precipitated by the addition of 2 vol EtOH andthen and dissolved in one tenth of the original volume of SDS sample buffer. Gels were stained with Coomassie Brilliant Blue G250.

### Vector Construction and Generation of Gene Deletion Mutants

Deletion cassettes consisting of 1.-to 1.5 kb fragments of the gene-specific flanking regions interrupted by the *T. reesei pyr4* marker gene (encoding orotidine-5′-monophosphate decarboxylase) were assembled by yeast recombinational cloning [32]. Oligonucleotides (10 µM) 5F +5R and 3F +3R were used for amplification of the individual flanking regions from genomic *T. reesei* DNA using *Taq* Polymerase (Promega). By PCR approximately 19 bp were introduced at each flanking end that overlap with the pRS426 (URA +) yeast shuttle vector or the *pyr4* gene to allow homologous recombination. A 3.2 kb fragment of the *T. reesei pyr4* marker gene was amplified with oligonucleotides Pyr4 fw and Pyr4 rev. A PCR touchdown program ranging from 62°C to 58°C for annealing was used for amplification. Oligonucleotide sequences are shown in Table S4. Yeast transformation was performed as described [33] using the lithium chloride/polyethylene glycol procedure. Yeast strain WW-YH10 (ATCC 208405) was transformed with both flanking regions, the *pyr4* marker gene and an EcoRI/XbaI digested plasmid pRS426 [34]. Transformants were selected on synthetic complete dropout media (SC-URA with uracil dropped out). Following total DNA isolation from liquid SC-URA media [35], plasmids were introduced into chemically competent JM109 *E. coli* cells (Promega). The outside primers 5F +3R were used to synthesize the complete deletion cassette from *S. cerevisiae*.

For construction of a strain overexpressing Trire2:3405 under the constitutive *tef1* promoter, the coding region of Trire2:3405 plus approximately 500 bp downstream of it were amplified using primer st_infusion_1 and st_infusion_2 (Table S4) and inserted into the SalI/HindIII linearized plasmid pLHhph1-tef1 [12] using the In-Fusion® HD Cloning Kit (Clontech). Transformation of *T. reesei* protoplasts was performed as described by Gruber *et al.* [36]. Integration of the *ptef1::3405* fragment was verified using primer Ptef F-ch and St R-ch (Table S4).

### Transformation of *T. reesei* and Analysis of Transformants

All deletion strains were generated in the *Δtku70* strain [27] and the QM 9414 strain (ATCC 26921) was used as a control in all experiments. Protoplast preparation and transformation were performed as previously described [36]. The deletion cassettes were purified from agarose gels (QIAquick Gel Extraction kit, QIAGEN) and concentrations were determined (Nanodrop Spectrophotometer, Peqlab). After transformation protoplasts were stabilized and regenerated on minimal mediumsupplemented with 1 M D-Sorbitol. In the case of the QM9414 *tef1::3405* strain the selection media additionally contained 100 µg/mL hygromycin B (Roth). For sporulation, transformants were transferred to small plates and purified by plating conidiospores onto plates with 0.1% Triton X-100 as colony restrictor. Single colonies were transferred to selective media and screened for correct integration of the deletion cassettes or of the *tef1::3405* fragment, respectively. Genomic DNA of the transformants was extracted [37] and transformants were screened for the presence of the deletion cassettes by amplifying a fragment by PCR with one oligonucleotide specific for the upstream region outside of the deletion cassette (primer abbreviated with “ch” in Table S4) and the second specific for the *pyr4* marker gene (ch_pyr4 neu).

### Transcriptome Analysis

Mycelia were harvested from cultures growing on lactose, glucose and glycerol, respectively, for 24 hrs. Total RNAs were extracted using TRIzol® reagent (Invitrogen Life Technologies, Carlsbad, CA, USA), according to the manufacturer’s instructions, and then purified. cDNA synthesis, labelling and hybridization was performed by Roche NimbleGen (Roche-NimbleGen, Inc., Madison, WI, USA) with a high density oligonucleotide (HDO) microarray using 60-mer probes representing the 9.129 genes of *T. reesei*. Microarray scanning, data acquisition and identification of probe sets showing a significant difference (p = 0.05) in expression level between the different conditions were performed essentially as described by Metz *et al.* [22]. Gene accession numbers were annotated according to version 2 of the *T. reesei* genome assembly (http://genome.jgi-psf.org/Trire2/Trire2.home.html), and ambiguous cases annotated manually.

Genes were then classified according to their major annotation in the MIPS Functional Catalogue [38]. To determine whether there were differences in the functional categories in each cluster, the distribution within each cluster was compared to the total distribution of all the annotated genes using independent chi-square tests. The microarray data and the related protocols are available at the GEO web site (www.ncbi.nlm.nih.gov/geo/) under accession number GSE39276.

### Real Time PCR

DNase treated (DNase I, RNase free; Fermentas) RNA (5 µg) was reverse transcribed with the RevertAid™ First Strand cDNA Kit (Fermentas) according to the manufacturer’s protocol with a combination (1∶1) of the provided oligo-dT and random hexamer primers. All assays were carried out in 96-well plates which were covered with optical tape, as described by Metz *et al*. [22]. Primers, amplification efficiency and R-square values are given in Table S5. Determination of the PCR efficiency was performed using triplicate reactions from a dilution series of cDNA, and the amplification efficiency was then calculated from the given slopes in the IQ5 Optical system Software v2.0. Expression ratios were calculated using REST© Software. All samples were analyzed in two independent experiments with three replicates in each run.

### Phylogenetic Analysis

Phylogenomic relationship between Trire2:3405 and its closest neighbors in BLAST (blastP) was studied by performing a randomized bootstrap maximum-likelihood analysis using RAxML software [39], setting the bootstrap analysis to 1000 runs and the bootstrap random seed value to 12,311. The Dayhoff mutation data matrix was used for the analysis of the alignment.

## Supporting Information

Figure S1 Growth of the MFS-knock out strains on glucose and cellobiose (1%, w/v, each) in the presence of 20 and 50 µg/mL nojirimycin.
(DOCX)

Table S1 Genes that are significantly (>2-fold) and consistently up- or downregulated in T. *reesei* on lactose when compared to glucose and glycerol.
(DOCX)

Table S2 Genes significantly regulated on lactose and by CRE1 and/or growth rate.
(DOCX)

Table S3 Expression rates of the ten most upregulated MFS permeases determined qPCR analysis.
(DOCX)

Table S4 Oligonucleotides used for the construction of deletion cassettes.
(DOCX)

Table S5 Oligonucleotides used in quantitative real-time PCR.
(DOCX)
