# Supplementary material for: Systems Analysis of Lactose Metabolism in Trichoderma reesei Identifies a Lactose Permease That Is Essential for Cellulase Induction
Source: PLoS One. 2013 May 8;8(5):e62631. doi: 10.1371/journal.pone.0062631 (PMC3648571; doi:10.1371/journal.pone.0062631)

**Figure S1.** Growth of the MFS-knock out strains on glucose and cellobiose (1 %, w/v, each) in the presence of 20 and 50 µg/mL nojirimycin. Photographs were taken after 96 h of growth.


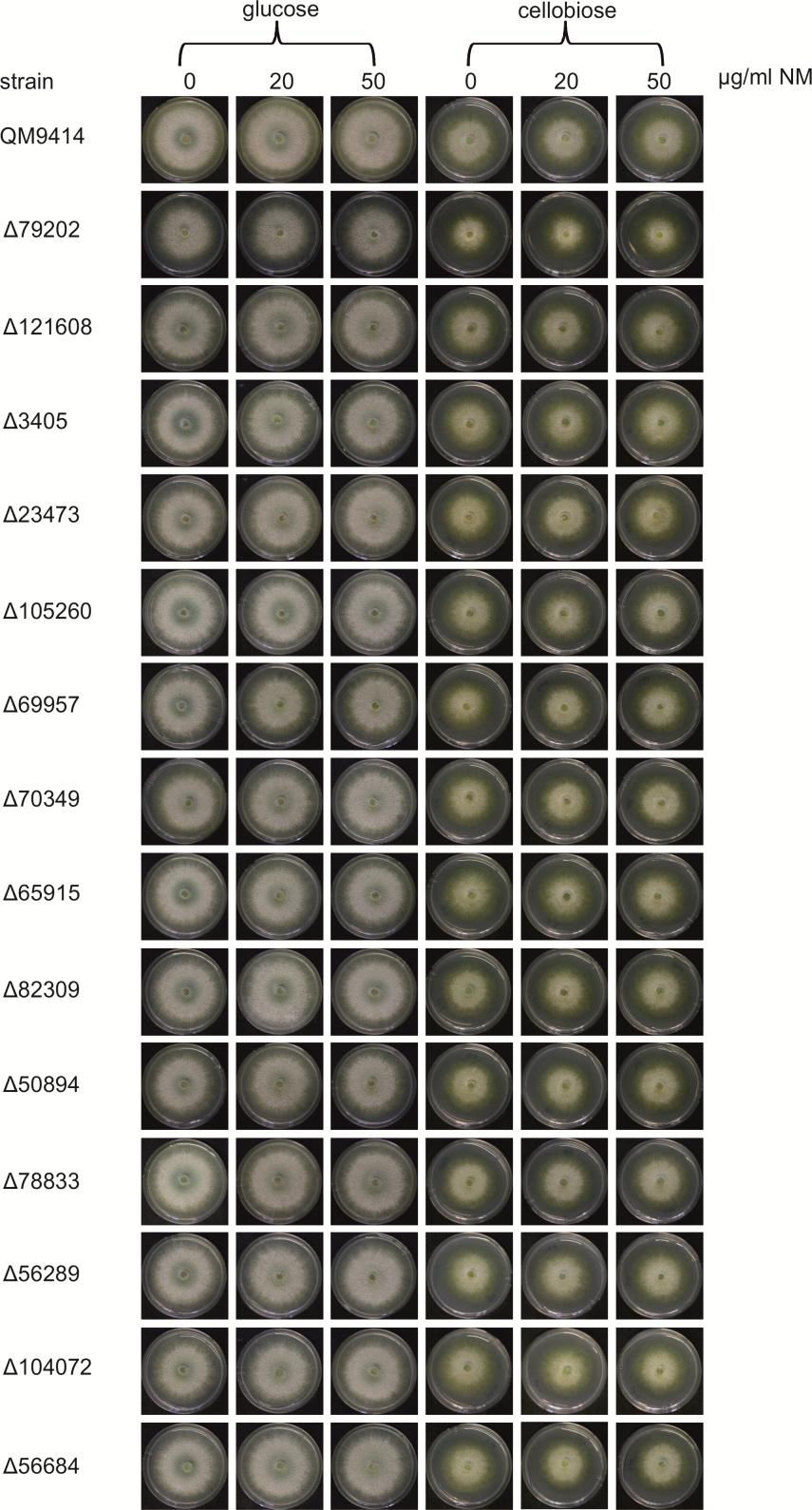

Supplement: Figure S1 — Growth of the MFS-knock out strains on glucose and cellobiose (1%, w/v, each) in the presence of 20 and 50 µg/mL nojirimycin. (DOCX) [file pone.0062631.s001.docx]
