# Supplementary material for: Systems Analysis of Lactose Metabolism in Trichoderma reesei Identifies a Lactose Permease That Is Essential for Cellulase Induction
Source: PLoS One. 2013 May 8;8(5):e62631. doi: 10.1371/journal.pone.0062631 (PMC3648571; doi:10.1371/journal.pone.0062631)
Supplement: Table S1 — Genes that are significantly (>2-fold) and consistently up- or downregulated in T. reesei on lactose when compared to glucose and glycerol. (DOCX) [file pone.0062631.s002.docx]

**Table S1.** Genes that are significantly (> 2-fold) and consistently up- or downregulated in T. *reesei* on lactose when compared to glucose and glycerol.

| **Protein ID** | **GL*** | **YL**** | **P value** | **F value** | **Annotation** |
| --- | --- | --- | --- | --- | --- |
|  | **upregulated genes** | | |  |  |
| 60490 | 4,384 | 3,01 | 0,0113 | 72,307 | 2-nitropropane dioxygenase |
| 3506 | 3,437 | 2,372 | 0,00418 | 261,53 | 2OG-Fe(II) oxygenase superfamily protein |
| 65029 | 3,107 | 3,791 | 0,0108 | 77,299 | 2OG-Fe(II) oxygenase superfamily protein |
| 102957 | 3,754 | 3,924 | 0,0107 | 78,9 | 2-oxoglutarate-dependent ethylene/succinate-forming enzyme, putative |
| 67117 | 2,301 | 4,095 | 0,0179 | 43,59 | 3'-5' exonuclease |
| 57647 | 3,58 | 2,593 | 0,00264 | 577,898 | 3-beta hydroxysteroid dehydrogenase/isomerase, putative |
| 112247 | 40,956 | 34,39 | 0,00358 | 330,51 | 3-isopropylmalate dehydrogenase |
| 112521 | 5,966 | 2,578 | 0,00172 | 1241,69 | 4,5-dihydroxyphthalate decarboxylase |
| 121405 | 22,901 | 4,347 | 0,00217 | 894,855 | 4-aminobutyrate aminotransferase |
| 121856 | 8,939 | 10,909 | 0,00275 | 526,06 | 4-hydroxyphenylpyruvate dioxygenase |
| 60018 | 3,243 | 2,856 | 0,0327 | 23,329 | AAA ATPase |
| 62693 | 11,378 | 7,257 | 0,000683 | 25592,43 | ABC-transporter Ste6p |
| 5107 | 20,412 | 106,681 | 0,00326 | 389,023 | ACC deaminase [Trichoderma asperellum] |
| 54870 | 5,099 | 2,008 | 0,00558 | 172,193 | acetate—CoA ligase |
| 59843 | 4,557 | 2,192 | 0,00573 | 167,88 | acetoacetyl-CoA synthase-like protein |
| 77093 | 23,571 | 30,769 | 0,00224 | 812,893 | acid sphingomyelin phosphodiesterase (probably vacuolar) |
| 82403 | 2,359 | 2,236 | 0,00394 | 287,054 | Acyl-CoA dehydrogenase |
| 107947 | 10,578 | 6,603 | 0,00532 | 184,567 | Acyl-CoA synthetase |
| 53372 | 3,294 | 4,408 | 0,0111 | 74,925 | acyltransferase 3 |
| 69753 | 5,701 | 2,629 | 0,0038 | 302,917 | ADA, Adenosine deaminase |
| 59053 | 3,155 | 3,416 | 0,00363 | 325,269 | Adenosine/AMP deaminase |
| 81260 | 2,626 | 2,008 | 0,0179 | 43,504 | adenylate kinase |
| 58412 | 2,419 | 6,851 | 0,00779 | 113,709 | AdhP Zn-dependent alcohol dehydrogenases |
| 120696 | 3,367 | 2,96 | 0,026 | 29,161 | alcohol dehydrogenase |
| 2038 | 8,149 | 10,088 | 0,00148 | 1911,912 | Alcohol dehydrogenase, class IV |
| 68590 | 20,819 | 20,229 | 0,00122 | 2548,292 | Alcohol dehydrogenase, class V |
| 80659 | 70,153 | 203,478 | 0,000856 | 7212,291 | alcohol oxidase AOX1 |
| 123274 | 5,456 | 21,653 | 0,0023 | 753,58 | Aldehyde dehydrogenase |
| 105363 | 5,561 | 5,447 | 0,00146 | 1960,249 | aldolase/citrate lyase family protein |
| 81843 | 7,259 | 8,099 | 0,002 | 1009,632 | Alkaline phosphatase |
| 81022 | 4,222 | 3,303 | 0,0123 | 66,215 | allantoate permease, 10 TM domains |
| 70860 | 3,934 | 3,631 | 0,00203 | 989,677 | allantoate permease, 10 TM domains |
| 59642 | 4,689 | 4,629 | 0,00507 | 196,641 | alpha beta hydrolase |
| 32243 | 5,453 | 4,557 | 0,00308 | 423,66 | Alpha/beta hydrolase |
| 62576 | 25,852 | 3,809 | 0,00466 | 223,281 | alpha/beta hydrolase |
| 66614 | 2,992 | 4,979 | 0,00335 | 372,81 | alpha/beta hydrolase |
| 63202 | 3,929 | 4,415 | 0,00466 | 223,669 | alpha/beta hydrolase, |
| 67408 | 4,781 | 4,698 | 0,00653 | 141,696 | AMA1, activator of meiotic anaphase promoting complex |
| 80645 | 3,848 | 4,841 | 0,0015 | 1887,16 | Amidase |
| 108885 | 3,349 | 3,291 | 0,0137 | 58,17 | amidase |
| 27697 | 3,135 | 5,072 | 0,00151 | 1683,557 | Amidase |
| 70859 | 2,467 | 3,726 | 0,00525 | 187,946 | amidase |
| 5889 | 2,392 | 2,353 | 0,0412 | 18,428 | amidase |
| 70375 | 11,354 | 4,907 | 0,00839 | 103,992 | amidase |
| 68615 | 10,045 | 3,087 | 0,00535 | 182,533 | Amidase |
| 123611 | 2,108 | 3,358 | 0,00532 | 184,281 | amino acid permease |
| 58511 | 3,549 | 13,799 | 0,00394 | 284,491 | amino acid permease (GABA) |
| 70098 | 134,839 | 19,457 | 0,00391 | 289,908 | amino acid permease (GABA) |
| 62172 | 3,319 | 2,631 | 0,0344 | 22,113 | amino acid permease (PotE?) |
| 23415 | 4,576 | 15,128 | 0,0119 | 68,507 | amino acid permease Dip5 |
| 64971 | 2,042 | 4,11 | 0,0327 | 23,341 | Amino acid permeases |
| 121942 | 2,038 | 2,258 | 0,0145 | 54,613 | amino acid transporter |
| 66819 | 668,102 | 107,627 | 0,00165 | 1292,594 | Amino acid transporter LysP |
| 61114 | 6,113 | 7,261 | 0,0111 | 74,322 | Amino acid transporter LysP |
| 59952 | 30,417 | 18,254 | 0,0047 | 219,884 | Amino acid transporter PotE |
| 56314 | 20,106 | 20,309 | 0,00136 | 2200,507 | Amino acid transporter PotE |
| 104077 | 107,73 | 121,74 | 0,00149 | 1883,438 | Amino acid transporter PotE |
| 109122 | 3,208 | 9,312 | 0,00331 | 378,659 | Amino acid transporters |
| 5787 | 2,968 | 4,266 | 0,0151 | 52,213 | Amino acid transporters |
| 55723 | 15,329 | 6,24 | 0,0062 | 151,496 | amino acidtransporter PotE |
| 55190 | 2,499 | 3,652 | 0,0175 | 44,537 | aminotransferase |
| 110414 | 2,855 | 2,401 | 0,0279 | 27,253 | Aminotransferase class-III |
| 103193 | 4,961 | 9,095 | 0,00521 | 189,428 | ankyrin |
| 82321 | 3,275 | 24,823 | 0,00249 | 639,641 | Aquaporin |
| 105870 | 5,203 | 5,223 | 0,00661 | 139,102 | Aquaporin |
| 55179 | 4,085 | 4,504 | 0,000848 | 10270,15 | Arginase family protein |
| 82619 | 2,344 | 8,51 | 0,00428 | 251,064 | arginosuccinate synthetase |
| 59402 | 13,969 | 6,135 | 0,0122 | 66,545 | arsenate reductase Arc2 |
| 123723 | 6,691 | 4,804 | 0,00508 | 195,959 | Arylacetamide deacetylase |
| 68161 | 2,235 | 2,507 | 0,00518 | 190,551 | Arylacetamide deacetylase |
| 70816 | 2,261 | 3,064 | 0,0107 | 78,686 | Asparaginase |
| 104073 | 7,79 | 3,632 | 0,0178 | 43,732 | aspartate racemase |
| 53961 | 28,55 | 77,792 | 0,00341 | 358,568 | Aspartyl protease |
| 34493 | 7,451 | 5,858 | 0,00231 | 746,678 | a-type peptide pheromone precursor hpp1 |
| 67639 | 6,908 | 4 | 0,00338 | 362,65 | benzoylformate decarboxylase |
| 68019 | 12,111 | 16,303 | 0,00301 | 447,947 | beta-lactamase superfamily |
| 54809 | 2,074 | 2,526 | 0,0296 | 25,746 | bicarbonate transporter |
| 69228 | 3,162 | 2,714 | 0,0123 | 65,847 | BioA Adenosylmethionine--amino--oxononanoate aminotransferase |
| 72524 | 2,559 | 9,521 | 0,00395 | 283,483 | bZIP transcription factor |
| 109538 | 8,644 | 15,467 | 0,00473 | 217,7 | bZIP transcriptional regulator |
| 120365 | 8,351 | 5,02 | 0,00501 | 199,705 | bZIP transcriptional regulator |
| 111567 | 13,597 | 7,103 | 0,0022 | 836,587 | C2H2 transcription factor |
| 62805 | 4,989 | 3,269 | 0,0247 | 30,764 | C2H2 transcriptional regulator |
| 120698 | 4,303 | 6,31 | 0,0044 | 242,52 | C2H2 transcriptional regulator |
| 102920 | 4,087 | 2,809 | 0,00538 | 181,579 | C2H2 transcriptional regulator |
| 120597 | 3,338 | 2,163 | 0,0132 | 60,775 | C2H2 transcriptional regulator |
| 4921 | 2,335 | 2,117 | 0,0432 | 17,556 | C2H2 transcriptional regulator |
| 22785 | 52,32 | 2,493 | 0,00179 | 1183,999 | C2H2 transcriptional regulator (amdA ?) |
| 34985 | 2,529 | 3,136 | 0,0134 | 59,448 | C-5 cytosine-specific DNA methylase |
| 124222 | 2,898 | 4,37 | 0,00972 | 87,255 | CaaX-protease, related to E. nidulans rce1, involved in signal transduction |
| 4171 | 6,847 | 5,564 | 0,0064 | 146,096 | calcium transporter |
| 68169 | 6,632 | 3,729 | 0,00102 | 5059,531 | Calcium transporter |
| 4875 | 4,63 | 2,19 | 0,0103 | 82,404 | carboxy-cis,cis-muconate cyclase |
| 78828 | 9,748 | 3,161 | 0,0078 | 113,216 | carboxylesterase type B |
| 22459 | 10,917 | 2,56 | 0,00251 | 626,608 | carboxypeptidase A |
| 122240 | 2,169 | 2,072 | 0,00656 | 140,805 | Carnitine o-acyltransferase |
| 67538 | 7,928 | 16,147 | 0,00111 | 4087,484 | Catalase |
| 58472 | 3,5 | 25,912 | 0,00183 | 1147,884 | Catalase |
| 120371 | 21,868 | 319,383 | 0,00122 | 2616,7 | Catalase |
| 70600 | 16,104 | 16,109 | 0,00425 | 254,05 | catalase, large subunit type |
| 62165 | 64,167 | 6,981 | 0,000918 | 5929,59 | catechol dioxigenase |
| 4876 | 3,851 | 4,154 | 0,00717 | 124,984 | catechol dioxygenase |
| 81430 | 39,767 | 6,041 | 0,00301 | 446,146 | Cation transporting ATPase |
| 81536 | 26,086 | 27,307 | 0,000213 | 116003,4 | cation transporting ATPase |
| 111094 | 16,549 | 21,247 | 0,000909 | 27702,74 | CBM 13 |
| 109235 | 19,344 | 63,498 | 0,00344 | 355,279 | CBM 18 |
| 72072 | 11,079 | 14,904 | 0,00356 | 335,522 | CE1 esterase (PHB?) |
| 65215 | 12,104 | 2,749 | 0,00615 | 153,31 | CE4 polysacharide deacetylase |
| 54219 | 5,676 | 5,861 | 0,00149 | 1875,533 | CE5 acetyl xylan esterase |
| 73632 | 5,339 | 7,325 | 0,00556 | 173,453 | CE5 acetyl xylan esteraseAXE1 |
| 60489 | 52,831 | 21,566 | 0,00451 | 233,092 | CE5 cutinase |
| 120784 | 4,27 | 2,734 | 0,00358 | 330,862 | cell wall mannoprotein |
| 104277 | 13,936 | 19,001 | 0,00425 | 254,317 | cell wall protein, distantly related to A. niger CwpA. |
| 73638 | 29,011 | 30,747 | 0,00204 | 975,957 | CIP1 |
| 5084 | 18,504 | 52,654 | 0,00583 | 164,464 | conidiation-specific protein 10 |
| 76034 | 4,175 | 3,144 | 0,00401 | 276,611 | coper radical oxidase |
| 122963 | 3,453 | 2,027 | 0,00502 | 199,08 | Copper amine oxidase |
| 69426 | 3,614 | 8,173 | 0,00425 | 252,439 | copper transporter |
| 110471 | 9,363 | 9,575 | 0,00558 | 172,753 | Cu2+/Zn2+ superoxide dismutase SOD1 |
| 123029 | 12,335 | 10,927 | 0,00578 | 166,321 | Cu2+/Zn2+ superoxide dismutase SOD1 |
| 63152 | 4,189 | 3,837 | 0,0149 | 53,056 | Cut9 interacting protein Scn1, putative |
| 41719 | 3,155 | 2,279 | 0,00597 | 159,035 | CutC family protein |
| 54166 | 2,978 | 2,937 | 0,00696 | 130,013 | Cytochrome P450 CYP2 subfamily |
| 66534 | 6,413 | 13,456 | 0,00784 | 112,447 | Cytochrome P450 CYP2 subfamily |
| 64900 | 5,385 | 2,09 | 0,00448 | 235,55 | Cytochrome P450 CYP2 subfamily |
| 70842 | 39,519 | 9,558 | 0,00222 | 822,715 | Cytochrome P450 CYP2 subfamily |
| 67964 | 36,348 | 5,461 | 0,00346 | 350,613 | Cytochrome P450 CYP2 subfamily |
| 27706 | 2,715 | 3,301 | 0,0259 | 29,265 | Cytochrome P450 CYP2 subfamily |
| 70956 | 8,511 | 3,943 | 0,00613 | 154,262 | cytochrome P450 monooxygenase |
| 58772 | 5,216 | 3,192 | 0,0034 | 360,753 | cytochrome P450 monooxygenase |
| 4726 | 3,695 | 3,329 | 0,00749 | 119,659 | Cytochrome P450 monooxygenase |
| 57555 | 16,416 | 14,241 | 0,00605 | 156,28 | cytochrome P450 monooxygenase |
| 68705 | 11,415 | 22,28 | 0,00334 | 374,263 | cytochrome P450 monooxygenase |
| 109746 | 5,829 | 7,358 | 0,00263 | 563,822 | cytochrome P450 monooxygenase |
| 73963 | 2,205 | 2,17 | 0,0178 | 43,84 | cytokinesis regulator, putative |
| 72137 | 18,462 | 32,25 | 0,00983 | 86,406 | cytosceleton binding protein |
| 59338 | 6,843 | 4,54 | 0,00653 | 142,498 | D-Alanine aminotransferase |
| 80034 | 2,733 | 3,433 | 0,00617 | 152,432 | D-amino acid oxidase |
| 4430 | 2,162 | 4,324 | 0,00951 | 89,711 | Developmental regulatory protein WetA |
| 21873 | 2,836 | 2,382 | 0,017 | 45,931 | dihydroxi acid dehydratase |
| 56830 | 6,506 | 10,396 | 0,00161 | 1446,599 | dipeptidyl peptidase 5 |
| 2044 | 4,139 | 5,619 | 0,00617 | 152,863 | dipeptidyl peptidase 5 |
| 63692 | 3,063 | 2,72 | 0,00603 | 156,928 | dipeptidyl peptidase 5 |
| 61293 | 29,995 | 10,718 | 0,00162 | 1410,417 | dipeptidyl peptidase 5 |
| 66394 | 4,206 | 6,044 | 0,0168 | 46,549 | DNA polymerase family X member (Mu) |
| 56780 | 2,048 | 2,022 | 0,0428 | 17,72 | DNA polymerase X family |
| 59964 | 2,066 | 2,058 | 0,0221 | 34,584 | DNA replication factor A subunit Ssb3, putative |
| 80714 | 3,863 | 2,199 | 0,0474 | 15,973 | dolichyl-phosphate-mannose a-mannosyltransferase |
| 61553 | 3,805 | 2,005 | 0,0077 | 115,895 | D-xylulose 5-phosphate/D-fructose 6-phosphate phosphoketolase |
| 70800 | 6,727 | 7,371 | 0,00941 | 91,084 | elastinolytic metalloproteinase |
| 3914 | 14,661 | 136,248 | 0,00356 | 338,885 | Endoplasmic reticulum protein EP58 |
| 110406 | 3,72 | 2,29 | 0,0084 | 103,917 | enoyl-CoA hydratase/isomerase |
| 5863 | 2,377 | 2,65 | 0,0331 | 23,045 | enoyl-CoA hydratase/isomerase |
| 82662 | 6,601 | 4,554 | 0,0139 | 57,032 | Epl1 |
| 58282 | 3,346 | 2,096 | 0,00802 | 109,688 | esterase family 9 |
| 70491 | 2,233 | 2,423 | 0,011 | 76,077 | esterase/lipase |
| 62230 | 2,125 | 4,801 | 0,0282 | 26,893 | esterase/lipase |
| 65677 | 3,73 | 2,581 | 0,00394 | 287,187 | Esterase/lipase/thioesterase |
| 105823 | 6,049 | 8,381 | 0,00898 | 95,698 | FAD binding protein |
| 66726 | 17,698 | 10,284 | 0,00704 | 127,987 | FAD binding protein |
| 104438 | 2,784 | 2,012 | 0,0217 | 35,376 | FAD binding protein |
| 5345 | 17,788 | 12,9 | 0,00317 | 407,622 | FAD binding protein |
| 67699 | 22,272 | 30,56 | 0,00444 | 238,448 | FAD/NAD-oxidoreductase, only in Hypocreaceae |
| 102820 | 2,312 | 3,073 | 0,0358 | 21,16 | ferrooxidoreductase |
| 76696 | 37,444 | 6,887 | 0,00263 | 564,981 | Flavin-containing monooxygenase |
| 70988 | 2,792 | 5,422 | 0,00876 | 98,783 | Flavin-containing monooxygenase |
| 76230 | 108,469 | 11,965 | 0,00419 | 260,06 | Flavin-containing monooxygenase |
| 63204 | 2,3 | 2,622 | 0,00928 | 92,328 | flavodoxin domain containing protein |
| 3719 | 3,377 | 3,119 | 0,0422 | 17,938 | Flavonol reductase/cinnamoyl-CoA reductase |
| 111716 | 2,286 | 25,491 | 0,00225 | 783,762 | Flavonol reductase/cinnamoyl-CoA reductase |
| 4146 | 8,555 | 7,201 | 0,00201 | 1003,081 | flavoprotein monooxygenase |
| 59095 | 6,411 | 3,602 | 0,00439 | 243,455 | Flavoprotein monooxygenase, putative |
| 56432 | 2,958 | 2,138 | 0,00771 | 115,777 | FMN-dependent dehydrogenase |
| 81511 | 4,843 | 10,002 | 0,00701 | 128,585 | formamidase |
| 66592 | 7,725 | 4,759 | 0,0184 | 42,23 | Fumarylacetoacetate (FAA) hydrolase |
| 5337 | 11,082 | 9,84 | 0,00544 | 178,68 | Gamma-glutamyltranspeptidase |
| 111236 | 2,607 | 5,72 | 0,00542 | 180,122 | GCN5 N-acetyltransferase |
| 2015 | 13,722 | 4,06 | 0,00374 | 312,165 | GCN5-related N-acetyltransferase |
| 33387 | 9,617 | 7,458 | 0,00825 | 105,738 | GCN5-related N-acetyltransferase, putative |
| 103451 | 2,136 | 4,412 | 0,00214 | 906,919 | GDP-forming succinate-CoA ligase, beta subunit |
| 32364 | 17,025 | 14,556 | 0,00806 | 108,556 | GDSL lipase |
| 56840 | 4,577 | 3,682 | 0,0105 | 80,354 | GFO_IDH_MocA dehydrogenase |
| 22197 | 4,052 | 9,923 | 0,00338 | 369,765 | GH1 ß-glucosidase |
| 120749 | 21,174 | 116,676 | 0,00635 | 147,346 | GH1 ß-glucosidase |
| 120229 | 8,373 | 8,239 | 0,00323 | 397,712 | GH10 xylanase |
| 123818 | 28,587 | 25,835 | 0,00273 | 531,445 | GH11 xylanase XYN2 |
| 123232 | 72,563 | 73,564 | 0,000887 | 12221,63 | GH12 endo-ß-1,4-glucanase |
| 108477 | 5,864 | 3,143 | 0,0104 | 81,118 | GH13 alpha-glucosidase |
| 55886 | 4,553 | 3,907 | 0,0369 | 20,544 | GH16 endo-ß-1,3/1,4-glucanase |
| 65162 | 7,393 | 2,217 | 0,00458 | 228,495 | GH18 chitinase |
| 77299 | 6,826 | 4,682 | 0,0121 | 67,687 | GH2 exo-ß-D-glucosaminidase |
| 57857 | 5,956 | 4,869 | 0,0039 | 294,53 | GH2 ß-mannosidase |
| 69245 | 101,158 | 38,535 | 0,00396 | 281,71 | GH2 ß-mannosidase |
| 103458 | 2,346 | 2,378 | 0,00439 | 243,601 | GH25 lysozyme |
| 72632 | 76,354 | 48,322 | 0,00442 | 241,031 | GH27 alpha-galactosidase |
| 72704 | 7,091 | 2,103 | 0,00501 | 199,917 | GH27 alpha-galactosidase |
| 65986 | 35,526 | 21,134 | 0,00119 | 2876,932 | GH27 alpha-galactosidase |
| 27259 | 3,285 | 3,374 | 0,0194 | 39,726 | GH27 alpha-galactosidase |
| 59391 | 23,278 | 25,785 | 0,00291 | 474,122 | GH27 alpha-galactosidase |
| 55999 | 5,679 | 4,295 | 0,0069 | 131,237 | GH27 alpha-galactosidase |
| 82227 | 8,106 | 8,403 | 0,0014 | 2072,773 | GH3 ß-glucosidase |
| 46816 | 5,255 | 6,969 | 0,0106 | 79,299 | GH3 ß-glucosidase |
| 108671 | 2,69 | 2,545 | 0,0221 | 34,681 | GH3 ß-glucosidase |
| 76672 | 2,108 | 2,121 | 0,0421 | 18,033 | GH3 ß-glucosidase |
| 121127 | 107,07 | 91,618 | 0,0011 | 3555,786 | GH3 ß-xylosidase |
| 111849 | 16,879 | 12,353 | 0,0035 | 345,698 | GH30 glucuronoyl-xylanase |
| 69276 | 4,691 | 3,5 | 0,00897 | 95,777 | GH30 glucuroyl xylanase XYN6 |
| 60085 | 3,819 | 11,31 | 0,00989 | 85,641 | GH31 alpha-glucosidase |
| 69944 | 3,73 | 4,186 | 0,0167 | 47,118 | GH31 alpha-xylosidase |
| 64827 | 2,979 | 2,612 | 0,00495 | 205,01 | GH36 alpha-galactosidase |
| 124016 | 12,7 | 5,756 | 0,00915 | 93,757 | GH36 alpha-galactosidase |
| 123226 | 35,271 | 14,146 | 0,00183 | 1134,939 | GH37 trehalase |
| 3739 | 233,319 | 63,254 | 0,00112 | 4158,457 | GH43 ß-xylosidase/a-L-arabinofuranosidase |
| 49976 | 12,862 | 24,602 | 0,00443 | 239,349 | GH45 endoglucanase V (EC 3.2.1.4); C-terminal CBM1 module |
| 45717 | 11,069 | 8,48 | 0,00302 | 439,293 | GH47 a-1,2-mannosidase (EC 3.2.1.113) |
| 120312 | 163,479 | 152,15 | 0,00132 | 2306,919 | GH5 endoglucanase 5A |
| 81087 | 62,912 | 76,828 | 0,00108 | 3504,589 | GH5 glycoside hydrolase |
| 123283 | 4,169 | 5,102 | 0,00817 | 106,933 | GH54 a-L-arabinofuranosidase |
| 55319 | 3,309 | 5,048 | 0,00178 | 1197,87 | GH54 a-L-arabinofuranosidase; C-terminal CBM42 module |
| 72567 | 273,081 | 274,233 | 0,00225 | 797,525 | GH6 cellobiohydrolase II (Cel6A) |
| 120961 | 62,728 | 62,882 | 0,00128 | 2371,059 | GH61 polysaccharide monooxygenase |
| 73643 | 21,043 | 25,74 | 0,000785 | 21039,43 | GH61 polysaccharide monooxygenase |
| 76210 | 10,795 | 11,667 | 0,00228 | 761,697 | GH62 a-L-arabinofuranosidase;identical to AAP57750.1 |
| 65137 | 3,654 | 6,227 | 0,0134 | 59,587 | GH64 ß-1,3-glucanase |
| 25224 | 5,701 | 8,362 | 0,00653 | 142,472 | GH65 a,a-trehalase |
| 123456 | 11,275 | 2,234 | 0,00412 | 266,761 | GH65 a,a-trehalase |
| 72526 | 275,106 | 218,78 | 0,00163 | 1542,668 | GH67 a-glucuronidase (EC 3.2.1.139) |
| 123989 | 246,472 | 91,566 | 0,00151 | 1707,905 | GH7 cellobiohydrolase I |
| 122081 | 3,3 | 3,913 | 0,0095 | 89,932 | GH7 endoglucanase Cel7B |
| 71532 | 2,649 | 7,183 | 0,00359 | 328,523 | GH71 α-1,3-glucanase |
| 49081 | 5,419 | 3,008 | 0,0137 | 57,783 | GH74 xyloglucanase (EC 3.2.1.151); C-terminal CBM1 module |
| 42152 | 2,83 | 6,149 | 0,0115 | 71,448 | GH75 chitosanase |
| 27395 | 3,292 | 2,494 | 0,0144 | 55,074 | GH76 a-1,6-mannanase |
| 55802 | 2,158 | 3,146 | 0,0206 | 37,367 | GH76 a-1,6-mannanase |
| 122495 | 19,358 | 20,326 | 0,00113 | 3904,601 | GH76 a-1,6-mannanase |
| 106575 | 5,015 | 3,634 | 0,0091 | 94,432 | GH79 ß-glycosidase |
| 79921 | 7,183 | 5,67 | 0,00323 | 397,576 | GH92 a-1,2-mannosidase |
| 74198 | 40,21 | 20,923 | 0,00652 | 142,789 | GH92 a-1,2-mannosidase |
| 5807 | 33,976 | 37,228 | 0,00346 | 352,371 | GH95 a-L-fucosidase |
| 58802 | 15,405 | 21,841 | 0,00663 | 138,713 | GH95 a-L-fucosidase |
| 71072 | 9,984 | 9,36 | 0,0129 | 62,425 | Gluconate kinase |
| 81778 | 8,115 | 4,276 | 0,00517 | 191,672 | glutaminase A |
| 112022 | 65,665 | 57,704 | 0,00248 | 649,68 | glutathione-S-transferase |
| 56819 | 3,647 | 5,234 | 0,0023 | 751,914 | glutathione-S-transferase |
| 123978 | 29,714 | 12,931 | 0,00228 | 763,035 | GMC oxidoreductase family protein |
| 60374 | 11,307 | 4,798 | 0,00315 | 414,258 | GMC oxidoreductase family protein |
| 105851 | 6,819 | 6,97 | 0,0217 | 35,296 | GMC oxidoreductase family protein |
| 67494 | 18,694 | 20,508 | 0,00158 | 1606,818 | Golgi GDP-mannose transporter |
| 63981 | 13,965 | 2,709 | 0,00232 | 740,142 | GPCR , contains RGS domain |
| 57526 | 15,982 | 16,307 | 0,00278 | 515,637 | GPCR, mating type pheromone G-protein coupled receptor |
| 123806 | 34,996 | 12,092 | 0,00609 | 155,438 | GPCR, secretin like |
| 72627 | 16,153 | 8,448 | 0,00544 | 178,372 | GPCR, secretin like |
| 64018 | 12,801 | 6,654 | 0,00323 | 397,272 | GPCR, mating type pheromone G-protein coupled receptor |
| 74807 | 3,086 | 3,263 | 0,00291 | 475,56 | GPI-anchored mannosidases |
| 73516 | 4,299 | 3,594 | 0,00965 | 87,975 | Grg1 glucose repressible protein |
| 78713 | 2,478 | 3,607 | 0,0136 | 58,766 | GT ß-(1-3) glucanosyltransferase |
| 63756 | 2,395 | 2,11 | 0,0144 | 54,808 | helicase, DEAD-box superfamily |
| 54230 | 6,183 | 4,988 | 0,00498 | 200,804 | HET domain protein with WD40 repeats |
| 30578 | 5,518 | 6,361 | 0,00413 | 266,14 | HhH-GPD family base excision DNA repair protein |
| 70630 | 4,523 | 4,42 | 0,00356 | 335,725 | homoserine acetyltransferase family protein |
| 61403 | 2,134 | 4,554 | 0,00961 | 88,284 | Hydantoinase/oxoprolinase |
| 106538 | 54,186 | 2,392 | 0,00449 | 234,751 | hydrophobin HFB5 |
| 110757 | 17,162 | 29,429 | 0,00241 | 691,487 | imidazole proprionase-related amidohydrolase |
| 123550 | 3,093 | 3,416 | 0,00672 | 136,214 | indoleamine 2,3-dioxygenase-like protein |
| 106276 | 2,251 | 2,699 | 0,0026 | 593,529 | initiator tRNA phosphoribosyl transferase. |
| 54962 | 3,369 | 4,127 | 0,00496 | 201,983 | iron transporter |
| 38812 | 154,968 | 301,65 | 0,0011 | 3654,54 | iron transporter |
| 104606 | 2,552 | 2,238 | 0,00535 | 182,946 | Iron-containing alcohol dehydrogenase |
| 54997 | 2,044 | 2,444 | 0,00635 | 147,473 | isoamyl alcohol oxidase |
| 31134 | 7,111 | 5,816 | 0,00163 | 1463,354 | isoprenylcysteine carboxyl methyltransferase |
| 56470 | 4,122 | 4,937 | 0,00346 | 350,96 | Lactate dehydrogenase |
| 59887 | 5,587 | 4,135 | 0,0127 | 63,433 | Lactate dehydrogenase, putative |
| 49753 | 7,746 | 4,695 | 0,0135 | 58,875 | L-arabinitol 4-dehydrogenase |
| 55240 | 3,131 | 5,523 | 0,00827 | 105,337 | large-conductance mechanosensitive channel |
| 78462 | 13,669 | 10,673 | 0,00287 | 484,832 | L-carnitine dehydratase/alpha-methylacyl-CoA racemase |
| 121418 | 67,182 | 64,176 | 0,000876 | 10418,9 | lipase G-D-S-L |
| 62651 | 7,094 | 5,155 | 0,00613 | 153,98 | long-chain fatty acid transporter, |
| 2076 | 17,589 | 35,171 | 0,00467 | 222,918 | malate permease |
| 79271 | 5,944 | 2,398 | 0,00291 | 475,892 | malate synthase |
| 65547 | 2,913 | 2,455 | 0,0339 | 22,443 | mandelate racemase/muconate lactonase-like protein |
| 109673 | 8,326 | 6,874 | 0,00393 | 286,051 | Mandelate racemase/muconate lactonizing enzyme |
| 104599 | 4,965 | 3,876 | 0,00425 | 252,67 | Mandelate racemase/muconate lactonizing enzyme |
| 58701 | 3,564 | 7,31 | 0,0113 | 72,372 | Mannose-6-phosphate isomerase |
| 124341 | 18,462 | 4,564 | 0,00301 | 443,846 | mating protein MAT1-2-1 |
| 71010 | 2,503 | 12,369 | 0,00518 | 190,347 | MDR-type ABC transporters |
| 1992 | 5,846 | 10,48 | 0,00403 | 274,792 | metal dependent phosphohydrolase |
| 68427 | 7,563 | 7,132 | 0,00249 | 636,528 | metal-dependent phosphohydrolase |
| 41208 | 3,484 | 3,516 | 0,00698 | 129,523 | metal-dependent phosphohydrolase |
| 65494 | 2,978 | 2,653 | 0,00502 | 198,477 | metallopeptidase |
| 3049 | 4,621 | 24,386 | 0,00603 | 156,894 | methionine aminopeptidase |
| 112230 | 2,141 | 2,51 | 0,0344 | 22,084 | Methylated-DNA-[protein]-cysteine S-methyltransferase |
| 112126 | 27,493 | 10,593 | 0,00445 | 237,583 | Methylmalonate semialdehyde dehydrogenase |
| 23237 | 2,399 | 2,679 | 0,016 | 49,067 | Methylthioadenosine phosphorylase MTAP |
| 48444 | 5,407 | 3,585 | 0,0225 | 34,066 | MFS maltose permease |
| 121441 | 150,779 | 98,893 | 0,00149 | 1775,503 | MFS permease |
| 69651 | 14,618 | 6,034 | 0,0111 | 74,223 | MFS permease |
| 69026 | 11,614 | 4,095 | 0,00712 | 125,727 | MFS permease |
| 46819 | 9,449 | 12,503 | 0,00245 | 669,685 | MFS permease |
| 27181 | 9,668 | 20,258 | 0,00616 | 152,837 | MFS permease |
| 61374 | 9,635 | 5,457 | 0,00443 | 240,225 | MFS permease |
| 28036 | 8,84 | 2,259 | 0,00668 | 137,344 | MFS permease |
| 67692 | 7,426 | 6,516 | 0,0107 | 78,717 | MFS permease |
| 60329 | 6,833 | 8,208 | 0,00627 | 149,719 | MFS permease |
| 119789 | 6,108 | 8,843 | 0,0026 | 584,823 | MFS permease |
| 123473 | 6,09 | 8,562 | 0,00314 | 413,907 | MFS permease |
| 104320 | 6,009 | 2,042 | 0,0207 | 37,187 | MFS permease |
| 80058 | 52,86 | 15,235 | 0,00212 | 942,03 | MFS permease |
| 123241 | 51,547 | 8,181 | 0,00216 | 892,758 | MFS permease |
| 105260 | 455,815 | 229,312 | 0,00142 | 2026,886 | MFS permease |
| 2068 | 4,781 | 5,214 | 0,00221 | 849,014 | MFS permease |
| 121608 | 4,754 | 13,203 | 0,00302 | 448,089 | MFS permease |
| 54972 | 4,403 | 6,843 | 0,00752 | 118,973 | MFS permease |
| 67541 | 4,19 | 4,378 | 0,0197 | 39,081 | MFS permease |
| 59796 | 3,655 | 3,402 | 0,00664 | 138,504 | MFS permease |
| 110430 | 3,356 | 2,232 | 0,0112 | 73,522 | MFS permease |
| 81670 | 3,276 | 3,478 | 0,00433 | 247,186 | MFS permease |
| 56095 | 28,314 | 10,78 | 0,00315 | 411,363 | MFS permease |
| 68925 | 26,14 | 27,259 | 0,00276 | 526,504 | MFS permease |
| 67334 | 2,937 | 4,058 | 0,0121 | 67,738 | MFS permease |
| 74953 | 2,833 | 4,595 | 0,0282 | 26,871 | MFS permease |
| 61278 | 2,717 | 2,119 | 0,0188 | 41,188 | MFS permease |
| 69611 | 2,291 | 3,446 | 0,0223 | 34,287 | MFS permease |
| 38341 | 2,196 | 2,06 | 0,00697 | 130,062 | MFS permease |
| 82309 | 17,116 | 24,504 | 0,00303 | 435,059 | MFS permease |
| 69957 | 16,247 | 19,213 | 0,00218 | 858,219 | MFS permease |
| 54632 | 14,665 | 27,341 | 0,0015 | 1803,946 | MFS permease |
| 56289 | 14,591 | 14,366 | 0,000791 | 9756,159 | MFS permease |
| 121482 | 13,313 | 4,959 | 0,0102 | 83,494 | MFS permease |
| 3405 | 116,196 | 100,633 | 0,00204 | 974,974 | MFS permease |
| 53611 | 10,917 | 7,803 | 0,00339 | 364,216 | MFS permease |
| 70108 | 10,627 | 6,64 | 0,00493 | 206,766 | MFS permease |
| 59388 | 10,545 | 15,151 | 0,008 | 110,217 | MFS permease |
| 111888 | 23,882 | 5,141 | 0,00254 | 617,64 | MFS permease |
| 106029 | 6,143 | 3,448 | 0,00368 | 318,515 | MFS permease |
| 68990 | 2,299 | 3,078 | 0,016 | 49,27 | MFS permease |
| 121986 | 16,852 | 10,335 | 0,00157 | 1376,44 | MFS permease |
| 50894 | 25,102 | 55,01 | 0,00279 | 511,414 | MFS permease |
| 68122 | 2,976 | 38,768 | 0,00279 | 509,946 | MFS permease |
| 78833 | 48,194 | 11,883 | 0,00182 | 1144,868 | MFS permease (fucose permease) |
| 22912 | 51,688 | 8,802 | 0,00242 | 688,041 | MFS permease (glucose permease HXT1) |
| 67469 | 2,175 | 2,978 | 0,00329 | 380,337 | MFS permease (maltose permease) |
| 76758 | 3,002 | 2,695 | 0,0252 | 30,193 | MFS permease (maltose) |
| 70349 | 13,794 | 14,424 | 0,00267 | 546,448 | MFS permease LIZ1 |
| 79202 | 33,668 | 86,395 | 0,00123 | 2580,639 | MFS permease, associated with cellulose signalling |
| 104072 | 26,321 | 37,835 | 0,00547 | 176,957 | MFS permease, xylose transporter |
| 4774 | 9,849 | 4,118 | 0,0047 | 219,788 | MFS transporter |
| 109677 | 8,267 | 7,511 | 0,00216 | 873,5 | MFS transporter |
| 71059 | 74,938 | 28,656 | 0,00249 | 641,536 | MFS transporter |
| 122153 | 7,803 | 10,495 | 0,00162 | 1494,032 | MFS transporter |
| 70932 | 7,686 | 7,237 | 0,0022 | 829,563 | MFS transporter |
| 55077 | 7,111 | 7,949 | 0,0422 | 17,977 | MFS transporter |
| 65915 | 47,552 | 10,203 | 0,00161 | 1559,517 | MFS transporter |
| 60177 | 4,812 | 2,621 | 0,00589 | 161,35 | MFS transporter |
| 53903 | 37,709 | 4,131 | 0,00356 | 338,042 | MFS transporter |
| 67752 | 31,13 | 4,412 | 0,00375 | 310,956 | MFS transporter |
| 5656 | 26,754 | 3,242 | 0,00459 | 227,967 | MFS transporter |
| 27770 | 24,516 | 6,507 | 0,00378 | 305,193 | MFS transporter |
| 59190 | 2,839 | 2,342 | 0,0236 | 32,371 | MFS transporter |
| 111724 | 2,324 | 2,659 | 0,0253 | 30,1 | MFS transporter |
| 76897 | 171,453 | 26,09 | 0,00224 | 781,833 | MFS transporter |
| 28409 | 17,17 | 2,189 | 0,00512 | 194,108 | MFS transporter |
| 56684 | 16,89 | 13,39 | 0,00668 | 137,396 | MFS transporter |
| 110133 | 3,27 | 2,096 | 0,0167 | 47,043 | Mitochondrial carrier protein |
| 47930 | 27,99 | 2,665 | 0,00149 | 1792,482 | Mitochondrial oxoglutarate/malate carrier proteins |
| 121743 | 33,979 | 6,851 | 0,0026 | 594,886 | Mitochondrial succinate/fumarate antiporter |
| 66854 | 7,636 | 23,909 | 0,00473 | 217,064 | Monocarboxylate transporter |
| 69282 | 4,354 | 8,396 | 0,00368 | 319,997 | monocarboxylate transporter |
| 55630 | 2,214 | 21,005 | 0,00373 | 313,748 | monocarboxylate transporter |
| 60116 | 4,98 | 5,333 | 0,00327 | 389,033 | MRP-type ABC transporter |
| 55636 | 4,155 | 4,108 | 0,0109 | 76,734 | MRP-type ABC transporter |
| 119790 | 9,121 | 5,115 | 0,00375 | 311,344 | myo-inositol oxygenase , |
| 47432 | 14,394 | 2,222 | 0,00263 | 577,585 | NAD(P) transhydrogenase beta subunit |
| 120943 | 3,557 | 2,247 | 0,00944 | 90,537 | NAD-dependent glutamate dehydrogenase |
| 76366 | 7,751 | 11,142 | 0,00259 | 590,009 | NADH:flavin oxidoreductase/12-oxophytodienoate reductase |
| 76204 | 3,349 | 5,004 | 0,00965 | 88,02 | NADH:flavin oxidoreductase/12-oxophytodienoate reductase |
| 53079 | 2,396 | 2,495 | 0,0112 | 73,416 | NADP/FAD dependent oxidoreductase |
| 3447 | 28,765 | 39,051 | 0,00188 | 1096,982 | NADP-dependent alcohol dehydrogenase (class V) |
| 122828 | 2,099 | 2,811 | 0,0205 | 37,562 | nicotinamide riboside kinase 1, putative |
| 60751 | 2,728 | 2,185 | 0,00661 | 139,354 | NRPS |
| 67189 | 2,387 | 2,421 | 0,0195 | 39,636 | NRPS |
| 22560 | 2,281 | 2,181 | 0,00406 | 270,881 | Nucleotide exchange factor Sil1 |
| 46794 | 4,221 | 12,688 | 0,0118 | 69,663 | oligopeptide transporter |
| 120017 | 2,414 | 4,723 | 0,0148 | 53,309 | oligopeptide transporter |
| 109487 | 3,876 | 2,384 | 0,0226 | 33,898 | oligopeptide transporter superfamily domain |
| 5366 | 3,191 | 4,969 | 0,00543 | 178,55 | O-methyltransferase, putative |
| 50323 | 10,571 | 5,924 | 0,0011 | 3266,006 | OOC1 |
| 76136 | 3,028 | 2,035 | 0,00803 | 109,425 | oxidoreductase |
| 70316 | 2,797 | 2,768 | 0,00548 | 176,781 | oxidoreductase, putative |
| 58479 | 2,785 | 2,352 | 0,0104 | 81,641 | oxidoreductase, unknown |
| 82327 | 3,948 | 6,807 | 0,014 | 56,674 | PDR-type ABC transporters |
| 61258 | 2,785 | 3,17 | 0,0137 | 57,736 | Peptidase C12, ubiquitin carboxyl-terminal hydrolase 1 |
| 4941 | 6,534 | 4,302 | 0,00324 | 397,764 | phenylacetyl-CoA ligase, |
| 60988 | 6,849 | 4,479 | 0,00355 | 336,818 | phosphate transporter |
| 111890 | 2,696 | 2,709 | 0,019 | 40,544 | Phosphate transporter |
| 124056 | 3,188 | 10,569 | 0,00466 | 223,412 | phosphatidic acid phosphatase |
| 104288 | 2,801 | 2,79 | 0,0129 | 62,378 | phosphatidylethanolamine-binding protein |
| 2492 | 3,834 | 2,668 | 0,0215 | 35,728 | Phosphatidylinositol-4-phosphate 5-kinase |
| 67579 | 2,056 | 8,166 | 0,0127 | 63,61 | phospholipase A2 |
| 73039 | 2,833 | 6,873 | 0,0137 | 58,133 | Phosphotransferase enzyme family |
| 80523 | 2,161 | 2,398 | 0,00936 | 91,564 | pH-response regulator protein palC |
| 81275 | 7,427 | 4,086 | 0,0041 | 268,368 | phytanoyl-CoA dioxygenase family protein |
| 111245 | 3,382 | 13,732 | 0,00245 | 668,451 | PL polysaccharide lyase; distantly related to chondroitin lyases |
| 46639 | 2,006 | 2,176 | 0,0112 | 73,403 | Prenylated Rab acceptor 1 Yip3 |
| 5371 | 2,298 | 3,63 | 0,0395 | 19,178 | Prolyl 4-hydroxylase, alpha subunit |
| 22093 | 3,354 | 4,094 | 0,00407 | 271,078 | Protein farnesyltransferase, alpha subunit |
| 106487 | 2,394 | 2,479 | 0,00858 | 101,404 | Protein kinase |
| 53776 | 2,118 | 2,335 | 0,0285 | 26,656 | Protein kinase |
| 122131 | 4,669 | 3,064 | 0,0328 | 23,25 | Protein-L-isoaspartate(D-aspartate) O-methyltransferase |
| 107042 | 6,148 | 2,903 | 0,00508 | 196,241 | PTH11 GPCR |
| 109146 | 54,534 | 52,972 | 0,000743 | 8662,295 | PTH11 GPCR |
| 39588 | 2,105 | 2,098 | 0,0233 | 32,888 | Pyridine nucleotide-disulphide oxidoreductase, |
| 65037 | 2,458 | 2,912 | 0,0224 | 34,092 | pyrophosphatase |
| 59267 | 2,804 | 3,272 | 0,0416 | 18,243 | pyruvate decarboxylase |
| 74282 | 2,12 | 8,081 | 0,00356 | 337,344 | QI74 orthologue |
| 80091 | 54,241 | 11,961 | 0,00359 | 329,933 | quinate permease, contains 11 transmembrane domains |
| 44278 | 2,663 | 59,72 | 0,00162 | 1405,441 | Rab geranyl transferase escort protein |
| 4213 | 5,725 | 4,956 | 0,0094 | 91,245 | ribonuclease T2 |
| 65615 | 5,079 | 4,153 | 0,00117 | 2789,177 | ribosomal RNA methyltransferase RrmJ/FtsJ domain. |
| 103482 | 4,072 | 4,518 | 0,0127 | 63,795 | RING-type zinc finger protein |
| 49048 | 4,486 | 5,807 | 0,00648 | 144,138 | RNA-dependent RNA-polymerase |
| 41325 | 3,298 | 8,467 | 0,0026 | 591,004 | RTA1 like protein; 7 TM |
| 70355 | 10,829 | 9,116 | 0,00514 | 193,188 | SAM-dependent methylransferase |
| 60758 | 3,117 | 3,381 | 0,0207 | 37,04 | SAM-dependent methyltransferase |
| 59381 | 8,575 | 5,409 | 0,00653 | 142,173 | SAM-dependent methyltransferases |
| 53428 | 4,243 | 9,599 | 0,00942 | 90,889 | SAM-dependent metyhltransferase |
| 124170 | 2,911 | 2,87 | 0,00379 | 305,655 | SAM-dependent S-methyltransferase |
| 61127 | 3,46 | 2,669 | 0,00394 | 286,419 | Serine carboxypeptidase |
| 121968 | 4,365 | 4,892 | 0,0173 | 45,149 | serine peptidase S28 |
| 108715 | 2,237 | 2,336 | 0,0238 | 32,065 | serine peptidase S28 |
| 59364 | 51,622 | 41,341 | 0,00221 | 837,262 | Sexual differentiation process protein ISP4 |
| 109687 | 18,837 | 9,599 | 0,00278 | 514,007 | Shikimate dehydrogenase |
| 65869 | 63,017 | 11,857 | 0,00253 | 621,22 | Shikimate kinase |
| 4990 | 5,477 | 5,406 | 0,0241 | 31,614 | short chain dehydrogenase/reductase |
| 106164 | 75,291 | 81,026 | 0,00087 | 6987,92 | short chain dehydrogenase/reductase |
| 70334 | 59,703 | 14,767 | 0,0013 | 2298,822 | short chain dehydrogenase/reductase |
| 60517 | 41,377 | 11,842 | 0,00142 | 2016,857 | short chain dehydrogenase/reductase |
| 22771 | 2,205 | 2,262 | 0,0249 | 30,619 | short chain dehydrogenase/reductase |
| 4682 | 19,45 | 8,755 | 0,00178 | 1208,023 | short chain dehydrogenase/reductase |
| 3055 | 19,283 | 21,189 | 0,00396 | 281,404 | short chain dehydrogenase/reductase |
| 77202 | 11,559 | 7,218 | 0,00163 | 1499,531 | short chain dehydrogenase/reductase |
| 54086 | 4,448 | 4,988 | 0,0111 | 74,38 | short-chain dehydrogenase, putative |
| 104557 | 2,358 | 2,789 | 0,00841 | 103,66 | short-chain dehydrogenase/reductase |
| 54227 | 7,858 | 34,194 | 0,00191 | 1078,1 | short-chain dehydrogenase/reductase |
| 54550 | 4,015 | 2,16 | 0,00392 | 285,719 | Short-chain dehydrogenase/reductase SDR |
| 81553 | 2,66 | 3,798 | 0,00942 | 90,886 | Short-chain dehydrogenase/reductase SDR |
| 66175 | 2,163 | 2,211 | 0,0156 | 50,342 | Short-chain dehydrogenase/reductase SDR |
| 66117 | 3,27 | 3,575 | 0,00425 | 255,273 | short-chain dehydrogenase/reductase SDR |
| 106138 | 2,961 | 2,546 | 0,0235 | 32,554 | SNF2 family DNA-dependent ATPase |
| 70197 | 22,433 | 22,313 | 0,00898 | 95,644 | ß-lactamase |
| 58717 | 7,997 | 5,282 | 0,0012 | 2879,618 | ß-lactamase class C |
| 107137 | 8,947 | 6,233 | 0,00151 | 1678,452 | sterol desaturase family |
| 123234 | 4,847 | 5,455 | 0,00739 | 121,16 | Subtilisin like protease (SUB9) |
| 51365 | 3,167 | 3,866 | 0,00712 | 125,903 | Subtilisin-like protease PPRC1 |
| 70383 | 3,877 | 3,444 | 0,0058 | 165,748 | succinate semialdehyde dehydrogenase, NADP |
| 111447 | 2,603 | 2,684 | 0,00358 | 333,039 | Sulfatase |
| 52073 | 2,097 | 2,639 | 0,00748 | 119,489 | sulfatase |
| 62367 | 7,75 | 3,136 | 0,00164 | 1471,154 | Sulfite oxidase, molybdopterin-binding component |
| 76601 | 3,972 | 24,483 | 0,00386 | 297,47 | Sulfite oxidase, molybdopterin-binding component |
| 106695 | 2,498 | 2,014 | 0,0119 | 68,688 | Sulfite oxidase, molybdopterin-binding component |
| 123992 | 15,986 | 4,71 | 0,00527 | 187,073 | swollenin |
| 122736 | 4,344 | 6,493 | 0,0158 | 49,614 | tagatose bisphosphate aldolase |
| 105238 | 7,947 | 10,067 | 0,00558 | 172,152 | Taurine catabolism dioxygenase TauD/TfdA |
| 42866 | 4,987 | 6,558 | 0,00887 | 96,969 | thioesterase family protein |
| 57322 | 3,555 | 7,972 | 0,0037 | 317,196 | Transcriptional regulator ARO (DNA-binding HTH domain) |
| 105978 | 2,806 | 6,185 | 0,0227 | 33,652 | Transcriptional regulator ARO8 (DNA-binding HTH domain) |
| 64175 | 2,76 | 2,545 | 0,00849 | 102,692 | transcriptional regulator HMG type |
| 112330 | 3,055 | 4,592 | 0,00162 | 1448,049 | transcriptional regulator, putative |
| 59740 | 2,365 | 3,073 | 0,0233 | 32,776 | transcriptional regulator, unknown |
| 2211 | 10,07 | 4,468 | 0,00578 | 166,272 | Transketolase |
| 73897 | 4,007 | 3,815 | 0,00906 | 94,837 | Trypsin-like protease |
| 65741 | 3,001 | 2,394 | 0,00349 | 347,349 | tryptophanyl-tRNA synthetase |
| 45445 | 6,39 | 2,04 | 0,00536 | 182,153 | tyrosinase |
| 58321 | 2,596 | 3,02 | 0,0461 | 16,401 | ubiquitin-conjugating enzyme |
| 108157 | 3,626 | 2,873 | 0,00502 | 199,199 | unique protein |
| 112520 | 52,284 | 8,025 | 0,00171 | 1249,486 | unique protein |
| 122087 | 14,315 | 5,52 | 0,00493 | 206,336 | unique protein |
| 111527 | 11,521 | 5,983 | 0,00248 | 650,07 | unique protein |
| 111566 | 9,592 | 8,885 | 0,00388 | 293,235 | unique protein |
| 123236 | 8,656 | 6,975 | 0,00532 | 184,413 | unique protein |
| 111050 | 8,138 | 8,864 | 0,00178 | 1209,82 | unique protein |
| 121594 | 7,99 | 9,637 | 0,00217 | 865,154 | unique protein |
| 123777 | 7,759 | 8,524 | 0,0133 | 60,246 | unique protein |
| 112049 | 7,603 | 7,834 | 0,00179 | 1180,168 | unique protein |
| 105106 | 7,594 | 2,127 | 0,00775 | 114,552 | unique protein |
| 123095 | 7,477 | 2,758 | 0,0219 | 34,909 | unique protein |
| 111038 | 6,954 | 2,531 | 0,0113 | 72,456 | unique protein |
| 102906 | 6,665 | 6,316 | 0,015 | 52,572 | unique protein |
| 110440 | 6,634 | 3,16 | 0,000831 | 7511,71 | unique protein |
| 103172 | 6,148 | 5,167 | 0,00397 | 280,343 | unique protein |
| 109367 | 55,613 | 66,397 | 0,00108 | 3218,616 | unique protein |
| 110167 | 5,595 | 10,159 | 0,00425 | 255,282 | unique protein |
| 122614 | 5,44 | 9,42 | 0,00703 | 127,689 | unique protein |
| 106537 | 5,106 | 4,768 | 0,00539 | 181,165 | unique protein |
| 70972 | 49,977 | 141,26 | 0,00142 | 2003,48 | unique protein |
| 109972 | 4,786 | 3,692 | 0,00753 | 118,72 | unique protein |
| 103660 | 4,503 | 4,523 | 0,00527 | 186,91 | unique protein |
| 105722 | 4,466 | 24,263 | 0,00149 | 1795,852 | unique protein |
| 112112 | 4,116 | 4,497 | 0,0402 | 18,889 | unique protein |
| 105514 | 3,954 | 2,32 | 0,00985 | 86,064 | unique protein |
| 121396 | 3,83 | 4,959 | 0,0103 | 81,716 | unique protein |
| 105349 | 3,806 | 3,001 | 0,00767 | 116,379 | unique protein |
| 111951 | 3,772 | 5,971 | 0,0111 | 74,873 | unique protein |
| 119534 | 3,715 | 2,151 | 0,0111 | 74,044 | unique protein |
| 105844 | 3,697 | 12,946 | 0,00514 | 193,193 | unique protein |
| 110214 | 3,446 | 3,654 | 0,00669 | 137,524 | unique protein |
| 120504 | 3,44 | 2,062 | 0,00712 | 125,683 | unique protein |
| 110767 | 3,281 | 3,603 | 0,0195 | 39,471 | unique protein |
| 110323 | 3,03 | 8,78 | 0,00269 | 541,676 | unique protein |
| 106181 | 21,115 | 8,368 | 0,00203 | 985,019 | unique protein |
| 112540 | 2,98 | 3,98 | 0,00345 | 352,281 | unique protein |
| 107297 | 2,964 | 3,932 | 0,0183 | 42,334 | unique protein |
| 106242 | 2,861 | 4,613 | 0,00998 | 84,63 | unique protein |
| 123732 | 2,816 | 2,828 | 0,0309 | 24,693 | unique protein |
| 121082 | 2,813 | 2,975 | 0,00806 | 108,956 | unique protein |
| 106584 | 2,735 | 2,173 | 0,0461 | 16,423 | unique protein |
| 66092 | 2,561 | 3,087 | 0,00962 | 88,564 | unique protein |
| 111374 | 2,561 | 2,23 | 0,00802 | 109,613 | unique protein |
| 111731 | 2,352 | 2,067 | 0,0129 | 62,091 | unique protein |
| 105174 | 2,272 | 3,359 | 0,00544 | 178,771 | unique protein |
| 106554 | 2,15 | 3,936 | 0,00245 | 665,69 | unique protein |
| 110830 | 2,097 | 4,875 | 0,00702 | 128,476 | unique protein |
| 122198 | 13,631 | 6,486 | 0,0129 | 62,074 | unique protein |
| 107494 | 12,529 | 5,997 | 0,00221 | 838,511 | unique protein |
| 111443 | 11,899 | 10,406 | 0,00552 | 175,702 | unique protein |
| 107913 | 11,347 | 9,569 | 0,00338 | 367,965 | unique protein |
| 104867 | 5,292 | 2,17 | 0,00915 | 93,871 | unique protein |
| 107914 | 36,151 | 13,95 | 0,00162 | 1462,135 | unique protein |
| 106089 | 3,503 | 3,562 | 0,0107 | 78,623 | unique protein |
| 108870 | 2,82 | 3,175 | 0,00806 | 108,318 | unique protein |
| 103799 | 2,803 | 2,534 | 0,00266 | 550,03 | unique protein |
| 107218 | 2,697 | 2,707 | 0,00539 | 181,019 | unique protein |
| 102850 | 2,373 | 2,546 | 0,0245 | 31,139 | unique protein |
| 124259 | 19,042 | 15,456 | 0,00373 | 314,148 | unique protein |
| 112400 | 5,18 | 3,943 | 0,00681 | 134,044 | unique protein |
| 112276 | 3,868 | 4,806 | 0,0214 | 35,823 | unique protein |
| 106958 | 3,72 | 3,471 | 0,00636 | 147,73 | unique protein |
| 112064 | 3,293 | 2,008 | 0,0156 | 50,537 | unique protein |
| 112459 | 2,575 | 2,087 | 0,00651 | 143,132 | unique protein |
| 106900 | 2,386 | 2,159 | 0,0188 | 41,202 | unique protein |
| 112325 | 2,368 | 2,544 | 0,00441 | 241,882 | unique protein |
| 109244 | 2,302 | 3,498 | 0,026 | 29,145 | unique protein |
| 104907 | 2,087 | 2,282 | 0,0186 | 41,67 | unique protein |
| 103153 | 11,828 | 4,823 | 0,00226 | 784,403 | unique protein |
| 104322 | 38,115 | 6,253 | 0,00239 | 712,681 | unique protein |
| 121475 | 4,667 | 2,379 | 0,0145 | 54,548 | unique protein |
| 109779 | 2,334 | 4,309 | 0,0171 | 45,567 | unique protein |
| 123797 | 9,705 | 3,853 | 0,00123 | 2556,426 | unique protein |
| 122975 | 5,515 | 6,409 | 0,00108 | 3820,305 | unique protein with collagen triple helix repeat |
| 107960 | 7,494 | 4,339 | 0,00363 | 324,71 | unique protein with WSC domain |
| 121285 | 3,862 | 6,535 | 0,0107 | 78,233 | unique protein, 1 TM |
| 111762 | 5,49 | 3,902 | 0,0167 | 47,235 | unique protein, 1TM |
| 59382 | 7,692 | 6,854 | 0,0119 | 68,755 | unknown protein |
| 53660 | 2,077 | 2,052 | 0,00778 | 113,83 | unknown protein |
| 120767 | 2,2 | 2,072 | 0,0368 | 20,604 | unknown protein |
| 53989 | 3,322 | 2,332 | 0,00491 | 207,355 | unknown protein |
| 61055 | 2,931 | 4,691 | 0,00266 | 554,556 | unknown protein |
| 105816 | 9,961 | 12,802 | 0,00602 | 157,181 | unknown protein |
| 54226 | 9,818 | 8,701 | 0,0059 | 161,001 | unknown protein |
| 70894 | 9,748 | 2,335 | 0,00285 | 493,463 | unknown protein |
| 53561 | 9,573 | 3,829 | 0,00309 | 422,437 | unknown protein |
| 110831 | 9,018 | 3,718 | 0,00368 | 319,655 | unknown protein |
| 103009 | 86,864 | 235,255 | 0,002 | 1015,098 | unknown protein |
| 67084 | 8,743 | 2,883 | 0,00346 | 350,093 | unknown protein |
| 75027 | 8,715 | 7,966 | 0,00301 | 445,133 | unknown protein |
| 123888 | 8,621 | 6,297 | 0,00686 | 132,143 | unknown protein |
| 108540 | 8,574 | 4,628 | 0,00779 | 113,553 | unknown protein |
| 62285 | 8,355 | 4,141 | 0,00482 | 212,426 | unknown protein |
| 67866 | 8,346 | 7,322 | 0,00553 | 175,374 | unknown protein |
| 121653 | 8,293 | 11,837 | 0,004 | 278,125 | unknown protein |
| 105718 | 8,222 | 4,883 | 0,00543 | 178,658 | unknown protein |
| 79726 | 7,738 | 2,374 | 0,0093 | 92,101 | unknown protein |
| 53777 | 7,526 | 7,289 | 0,0133 | 59,917 | unknown protein |
| 76713 | 7,459 | 2,169 | 0,00608 | 155,417 | unknown protein |
| 77743 | 7,383 | 4,242 | 0,00461 | 226,971 | unknown protein |
| 41425 | 7,057 | 5,854 | 0,00264 | 565,067 | unknown protein |
| 74508 | 6,873 | 4,231 | 0,0112 | 73,644 | unknown protein |
| 64274 | 6,811 | 6,814 | 0,00313 | 417,862 | unknown protein |
| 30166 | 6,617 | 5,377 | 0,0137 | 57,794 | unknown protein |
| 109925 | 6,592 | 3,507 | 0,00352 | 344,259 | unknown protein |
| 110342 | 6,586 | 7,938 | 0,00491 | 208,165 | unknown protein |
| 106371 | 6,539 | 6,993 | 0,00301 | 446,856 | unknown protein |
| 62114 | 6,294 | 5,88 | 0,00216 | 885,817 | unknown protein |
| 57643 | 51,591 | 54,072 | 0,00287 | 486,548 | unknown protein |
| 65739 | 5,992 | 4,886 | 0,00873 | 99,48 | unknown protein |
| 56117 | 5,933 | 2,672 | 0,00653 | 141,79 | unknown protein |
| 1999 | 5,867 | 2,026 | 0,0102 | 82,945 | unknown protein |
| 107217 | 5,801 | 7,269 | 0,00161 | 1390,892 | unknown protein |
| 110615 | 5,773 | 6,194 | 0,00107 | 4815,949 | unknown protein |
| 55126 | 5,747 | 6,658 | 0,00527 | 187,232 | unknown protein |
| 70808 | 5,68 | 4,567 | 0,0103 | 82,393 | unknown protein |
| 102966 | 5,652 | 7,807 | 0,0064 | 145,992 | unknown protein |
| 41518 | 5,648 | 2,689 | 0,00901 | 95,335 | unknown protein |
| 61164 | 5,633 | 4,08 | 0,00775 | 115,083 | unknown protein |
| 124092 | 5,501 | 3,006 | 0,00765 | 116,786 | unknown protein |
| 107853 | 5,464 | 10,37 | 0,0127 | 63,705 | unknown protein |
| 49366 | 5,448 | 3,855 | 0,00307 | 425,284 | unknown protein |
| 104302 | 5,425 | 6,149 | 0,00165 | 1304,413 | unknown protein |
| 102830 | 5,386 | 6,543 | 0,0126 | 64,06 | unknown protein |
| 121189 | 5,239 | 3,315 | 0,00519 | 190,061 | unknown protein |
| 106877 | 5,225 | 2,126 | 0,0134 | 59,726 | unknown protein |
| 81659 | 5,217 | 2,144 | 0,00503 | 198,867 | unknown protein |
| 104295 | 5,178 | 4,174 | 0,00454 | 230,869 | unknown protein |
| 59152 | 5,175 | 2,754 | 0,00464 | 225,147 | unknown protein |
| 109311 | 5,168 | 4,693 | 0,00251 | 631,492 | unknown protein |
| 105356 | 5,162 | 5,61 | 0,00473 | 218,037 | unknown protein |
| 107644 | 5,095 | 3,592 | 0,00849 | 102,796 | unknown protein |
| 4851 | 40,513 | 2,143 | 0,00201 | 1023,071 | unknown protein |
| 53500 | 4,91 | 5,797 | 0,0108 | 77,511 | unknown protein |
| 121439 | 4,891 | 2,385 | 0,00391 | 290,224 | unknown protein |
| 61939 | 4,834 | 4,563 | 0,0111 | 74,34 | unknown protein |
| 105287 | 4,83 | 23,757 | 0,00425 | 255,507 | unknown protein |
| 80685 | 4,822 | 4,027 | 0,0139 | 57,127 | unknown protein |
| 107495 | 4,803 | 10,926 | 0,00594 | 159,813 | unknown protein |
| 104425 | 4,796 | 4,259 | 0,031 | 24,61 | unknown protein |
| 40156 | 4,775 | 4,907 | 0,00482 | 213,828 | unknown protein |
| 41171 | 4,663 | 6,473 | 0,00653 | 142,172 | unknown protein |
| 120326 | 4,61 | 3,034 | 0,0183 | 42,607 | unknown protein |
| 105771 | 4,582 | 3,482 | 0,0108 | 77,325 | unknown protein |
| 111135 | 4,582 | 6,407 | 0,00263 | 575,727 | unknown protein |
| 2399 | 4,571 | 4,135 | 0,00403 | 275,48 | unknown protein |
| 66786 | 4,56 | 3,73 | 0,0126 | 64,323 | unknown protein |
| 62836 | 4,557 | 4,189 | 0,0126 | 64,109 | unknown protein |
| 79606 | 4,522 | 4,06 | 0,0112 | 73,164 | unknown protein |
| 54790 | 4,51 | 2,635 | 0,00625 | 150,117 | unknown protein |
| 110768 | 4,353 | 7,551 | 0,00302 | 439,342 | unknown protein |
| 52438 | 4,321 | 3,172 | 0,00887 | 97,225 | unknown protein |
| 66929 | 4,264 | 2,626 | 0,00527 | 186,557 | unknown protein |
| 124198 | 4,254 | 4,5 | 0,00703 | 127,864 | unknown protein |
| 78792 | 4,198 | 3,141 | 0,00447 | 236,082 | unknown protein |
| 40943 | 4,186 | 6,218 | 0,00329 | 380,342 | unknown protein |
| 22510 | 4,166 | 5,22 | 0,00404 | 272,325 | unknown protein |
| 41663 | 4,123 | 4,932 | 0,0119 | 68,616 | unknown protein |
| 105223 | 4,08 | 3,19 | 0,019 | 40,691 | unknown protein |
| 54372 | 4,077 | 2,952 | 0,00481 | 214,134 | unknown protein |
| 67286 | 4,057 | 2,31 | 0,0147 | 53,625 | unknown protein |
| 111059 | 4,016 | 7,906 | 0,00661 | 139,201 | unknown protein |
| 112660 | 4,014 | 3,685 | 0,0133 | 59,991 | unknown protein |
| 59372 | 32,478 | 37,477 | 0,00109 | 3509,344 | unknown protein |
| 53360 | 3,995 | 3,329 | 0,00378 | 307,393 | unknown protein |
| 59073 | 3,946 | 2,625 | 0,0217 | 35,332 | unknown protein |
| 64898 | 3,848 | 2,926 | 0,0219 | 35,015 | unknown protein |
| 106252 | 3,802 | 2,978 | 0,0343 | 22,145 | unknown protein |
| 21120 | 3,752 | 4,641 | 0,0126 | 64,127 | unknown protein |
| 123188 | 3,731 | 2,26 | 0,0111 | 74,674 | unknown protein |
| 57002 | 3,724 | 4,927 | 0,00586 | 163,012 | unknown protein |
| 109321 | 3,693 | 2,019 | 0,0135 | 59,207 | unknown protein |
| 51558 | 3,692 | 5,062 | 0,0153 | 51,372 | unknown protein |
| 2322 | 3,619 | 5,531 | 0,012 | 68,115 | unknown protein |
| 2703 | 3,6 | 3,111 | 0,0139 | 57,166 | unknown protein |
| 108669 | 3,595 | 3,606 | 0,00856 | 101,581 | unknown protein |
| 119619 | 3,573 | 3,292 | 0,00689 | 131,596 | unknown protein |
| 111168 | 3,557 | 4,035 | 0,0168 | 46,731 | unknown protein |
| 110595 | 3,544 | 2,274 | 0,0473 | 16,025 | unknown protein |
| 61097 | 3,531 | 2,445 | 0,013 | 61,818 | unknown protein |
| 60270 | 3,516 | 2,275 | 0,0101 | 84,142 | unknown protein |
| 57383 | 3,474 | 2,198 | 0,00303 | 434,427 | unknown protein |
| 112147 | 3,458 | 4,34 | 0,00424 | 254,038 | unknown protein |
| 110403 | 3,439 | 2,235 | 0,0183 | 42,66 | unknown protein |
| 107279 | 3,398 | 2,756 | 0,0147 | 53,645 | unknown protein |
| 57776 | 3,371 | 3,338 | 0,0213 | 36,052 | unknown protein |
| 106082 | 3,36 | 3,141 | 0,0238 | 31,979 | unknown protein |
| 56700 | 3,34 | 3,756 | 0,00659 | 139,893 | unknown protein |
| 76269 | 3,282 | 3,218 | 0,024 | 31,808 | unknown protein |
| 105617 | 3,281 | 2,88 | 0,0237 | 32,293 | unknown protein |
| 107111 | 3,28 | 2,543 | 0,0153 | 51,585 | unknown protein |
| 105917 | 3,274 | 2,302 | 0,0167 | 47,071 | unknown protein |
| 65735 | 3,273 | 3,87 | 0,00756 | 117,985 | unknown protein |
| 112285 | 3,262 | 5,355 | 0,00844 | 103,257 | unknown protein |
| 108158 | 3,214 | 3,924 | 0,0016 | 1445,352 | unknown protein |
| 107855 | 3,21 | 2,652 | 0,00659 | 139,987 | unknown protein |
| 67133 | 3,202 | 2,38 | 0,00458 | 228,539 | unknown protein |
| 124134 | 3,199 | 2,028 | 0,014 | 56,608 | unknown protein |
| 44624 | 3,197 | 2,034 | 0,0307 | 24,818 | unknown protein |
| 59940 | 3,193 | 5,122 | 0,0101 | 83,849 | unknown protein |
| 105765 | 3,153 | 3,423 | 0,0166 | 47,323 | unknown protein |
| 47286 | 3,117 | 2,71 | 0,0157 | 49,995 | unknown protein |
| 112551 | 3,102 | 5,125 | 0,00502 | 198,422 | unknown protein |
| 104137 | 3,073 | 3,965 | 0,0214 | 35,834 | unknown protein |
| 79222 | 3,052 | 20,105 | 0,00284 | 497,625 | unknown protein |
| 62053 | 3,021 | 5,243 | 0,00295 | 463,585 | unknown protein |
| 104463 | 3,015 | 2,246 | 0,0148 | 53,448 | unknown protein |
| 111194 | 3,009 | 3,073 | 0,039 | 19,458 | unknown protein |
| 60370 | 28,322 | 36,753 | 0,0036 | 327,397 | unknown protein |
| 60445 | 274,609 | 246,075 | 0,00113 | 4241,641 | unknown protein |
| 106615 | 27,113 | 7,444 | 0,0015 | 1717,913 | unknown protein |
| 104304 | 21,792 | 11,681 | 0,0015 | 1800,768 | unknown protein |
| 70373 | 21,105 | 19,08 | 0,00474 | 218,074 | unknown protein |
| 102411 | 2,983 | 3,227 | 0,0219 | 35,066 | unknown protein |
| 103336 | 2,969 | 3,13 | 0,00601 | 157,523 | unknown protein |
| 104820 | 2,965 | 3,123 | 0,00463 | 225,462 | unknown protein |
| 33632 | 2,95 | 2,315 | 0,0221 | 34,553 | unknown protein |
| 74515 | 2,932 | 2,592 | 0,00288 | 482,289 | unknown protein |
| 5347 | 2,925 | 3,879 | 0,00302 | 441,88 | unknown protein |
| 70329 | 2,914 | 2,796 | 0,00302 | 438,736 | unknown protein |
| 4659 | 2,905 | 2,037 | 0,0328 | 23,225 | unknown protein |
| 64834 | 2,903 | 5,032 | 0,00395 | 282,843 | unknown protein |
| 109410 | 2,898 | 2,914 | 0,0123 | 65,763 | unknown protein |
| 105051 | 2,886 | 2,318 | 0,00527 | 186,693 | unknown protein |
| 76117 | 2,878 | 2,999 | 0,00558 | 172,96 | unknown protein |
| 56593 | 2,876 | 2,46 | 0,0278 | 27,334 | unknown protein |
| 62484 | 2,859 | 4,297 | 0,0139 | 56,931 | unknown protein |
| 112390 | 2,859 | 4,135 | 0,0133 | 60,378 | unknown protein |
| 107775 | 2,854 | 8,618 | 0,00601 | 157,722 | unknown protein |
| 121163 | 2,804 | 5,157 | 0,0148 | 53,243 | unknown protein |
| 103063 | 2,802 | 2,864 | 0,0482 | 15,689 | unknown protein |
| 119607 | 2,793 | 2,366 | 0,00391 | 290,914 | unknown protein |
| 34353 | 2,787 | 3,274 | 0,0102 | 83,189 | unknown protein |
| 46266 | 2,776 | 3,809 | 0,00952 | 89,497 | unknown protein |
| 121065 | 2,752 | 2,743 | 0,00619 | 151,66 | unknown protein |
| 68430 | 2,747 | 2,215 | 0,0142 | 55,537 | unknown protein |
| 53401 | 2,734 | 2,584 | 0,00355 | 336,774 | unknown protein |
| 106828 | 2,721 | 2,307 | 0,00357 | 334,074 | unknown protein |
| 59056 | 2,707 | 2,617 | 0,00817 | 106,953 | unknown protein |
| 79990 | 2,707 | 3,209 | 0,0296 | 25,709 | unknown protein |
| 39606 | 2,704 | 2,139 | 0,034 | 22,421 | unknown protein |
| 107547 | 2,698 | 2,992 | 0,0165 | 47,707 | unknown protein |
| 4494 | 2,693 | 2,543 | 0,0484 | 15,621 | unknown protein |
| 111579 | 2,667 | 2,446 | 0,00962 | 88,462 | unknown protein |
| 62556 | 2,654 | 4,941 | 0,00489 | 208,817 | unknown protein |
| 45317 | 2,649 | 3,261 | 0,0363 | 20,894 | unknown protein |
| 103156 | 2,621 | 11,104 | 0,00581 | 165,067 | unknown protein |
| 103852 | 2,611 | 2,448 | 0,0128 | 63,029 | unknown protein |
| 107197 | 2,587 | 2,004 | 0,0375 | 20,198 | unknown protein |
| 104879 | 2,541 | 2,869 | 0,0267 | 28,445 | unknown protein |
| 43893 | 2,534 | 3,617 | 0,0187 | 41,425 | unknown protein |
| 121689 | 2,512 | 2,041 | 0,0305 | 25,021 | unknown protein |
| 5942 | 2,51 | 2,453 | 0,0185 | 41,865 | unknown protein |
| 40374 | 2,507 | 2,79 | 0,0106 | 79,627 | unknown protein |
| 102904 | 2,5 | 2,974 | 0,0104 | 81,002 | unknown protein |
| 63416 | 2,499 | 2,835 | 0,0249 | 30,559 | unknown protein |
| 112380 | 2,498 | 2,276 | 0,021 | 36,52 | unknown protein |
| 121187 | 2,496 | 3,005 | 0,0122 | 66,854 | unknown protein |
| 112193 | 2,474 | 5,565 | 0,00287 | 484,362 | unknown protein |
| 110174 | 2,461 | 3,952 | 0,0191 | 40,38 | unknown protein |
| 111476 | 2,421 | 2,345 | 0,00911 | 94,261 | unknown protein |
| 107552 | 2,397 | 2,242 | 0,0172 | 45,452 | unknown protein |
| 103446 | 2,392 | 2,867 | 0,0267 | 28,401 | unknown protein |
| 123710 | 2,381 | 3,587 | 0,0124 | 65,291 | unknown protein |
| 120774 | 2,375 | 3,666 | 0,0042 | 258,693 | unknown protein |
| 66286 | 2,374 | 2,035 | 0,0118 | 69,494 | unknown protein |
| 2343 | 2,37 | 2,916 | 0,00535 | 182,777 | unknown protein |
| 4677 | 2,333 | 2,42 | 0,0199 | 38,699 | unknown protein |
| 76065 | 2,305 | 2,199 | 0,0207 | 37,183 | unknown protein |
| 67324 | 2,303 | 3,12 | 0,0111 | 75,107 | unknown protein |
| 123019 | 2,302 | 3,103 | 0,0148 | 53,18 | unknown protein |
| 109708 | 2,291 | 2,406 | 0,0298 | 25,54 | unknown protein |
| 109929 | 2,287 | 2,51 | 0,0189 | 40,825 | unknown protein |
| 42326 | 2,282 | 2,164 | 0,00202 | 997,792 | unknown protein |
| 103930 | 2,276 | 3,202 | 0,0237 | 32,268 | unknown protein |
| 69416 | 2,265 | 2,462 | 0,00425 | 252,372 | unknown protein |
| 109883 | 2,247 | 4,039 | 0,00388 | 295,634 | unknown protein |
| 67605 | 2,243 | 4,022 | 0,00863 | 100,79 | unknown protein |
| 79677 | 2,241 | 3,451 | 0,0182 | 42,876 | unknown protein |
| 120320 | 2,238 | 2,089 | 0,0207 | 37,04 | unknown protein |
| 56026 | 2,221 | 4,297 | 0,00986 | 86,005 | unknown protein |
| 111129 | 2,21 | 2,168 | 0,00775 | 114,693 | unknown protein |
| 69933 | 2,204 | 3,966 | 0,00986 | 85,977 | unknown protein |
| 105538 | 2,19 | 2,141 | 0,0316 | 24,106 | unknown protein |
| 111253 | 2,178 | 2,097 | 0,00944 | 90,597 | unknown protein |
| 108806 | 2,169 | 2,448 | 0,0256 | 29,658 | unknown protein |
| 74971 | 2,163 | 3,524 | 0,00441 | 240,975 | unknown protein |
| 119568 | 2,144 | 2,353 | 0,0109 | 76,689 | unknown protein |
| 67035 | 2,136 | 3,227 | 0,0195 | 39,443 | unknown protein |
| 62979 | 2,134 | 2,187 | 0,0433 | 17,498 | unknown protein |
| 122187 | 2,125 | 3,658 | 0,0491 | 15,402 | unknown protein |
| 61222 | 2,112 | 2,303 | 0,0346 | 21,903 | unknown protein |
| 110496 | 2,107 | 2,13 | 0,0201 | 38,206 | unknown protein |
| 105682 | 2,091 | 2,219 | 0,0199 | 38,631 | unknown protein |
| 111604 | 2,091 | 2,15 | 0,0137 | 58,219 | unknown protein |
| 57237 | 2,086 | 2,752 | 0,0348 | 21,814 | unknown protein |
| 102619 | 2,082 | 2,252 | 0,00922 | 92,972 | unknown protein |
| 48852 | 2,08 | 2,139 | 0,0461 | 16,416 | unknown protein |
| 110493 | 2,079 | 2,423 | 0,0339 | 22,446 | unknown protein |
| 65164 | 2,062 | 4,135 | 0,0112 | 73,167 | unknown protein |
| 65992 | 2,037 | 2,017 | 0,0188 | 41,094 | unknown protein |
| 67868 | 2,021 | 2,382 | 0,0232 | 33,042 | unknown protein |
| 108537 | 2,019 | 2,727 | 0,0124 | 65,328 | unknown protein |
| 106569 | 2,018 | 2,038 | 0,00401 | 277,047 | unknown protein |
| 30478 | 2,016 | 2,582 | 0,0206 | 37,311 | unknown protein |
| 2499 | 2,008 | 5,385 | 0,0111 | 75,221 | unknown protein |
| 107591 | 2,007 | 2,268 | 0,0155 | 50,827 | unknown protein |
| 41362 | 2,006 | 2,099 | 0,0375 | 20,185 | unknown protein |
| 109444 | 2,005 | 2,116 | 0,0276 | 27,481 | unknown protein |
| 103725 | 2 | 2,586 | 0,00442 | 240,129 | unknown protein |
| 64656 | 18,108 | 2,895 | 0,00757 | 117,825 | unknown protein |
| 112239 | 170,439 | 258,778 | 0,000732 | 8546,835 | unknown protein |
| 106258 | 15,691 | 6,763 | 0,000993 | 12555,81 | unknown protein |
| 60560 | 14,627 | 14,642 | 0,00363 | 324,735 | unknown protein |
| 104215 | 12,542 | 13,045 | 0,00381 | 303,033 | unknown protein |
| 82374 | 12,324 | 2,9 | 0,00936 | 91,627 | unknown protein |
| 122089 | 11,574 | 9,932 | 0,0026 | 595,246 | unknown protein |
| 60565 | 11,267 | 11,255 | 0,00583 | 164,276 | unknown protein |
| 111362 | 10,952 | 16,034 | 0,00455 | 230,194 | unknown protein |
| 58639 | 10,63 | 2,173 | 0,00249 | 630,329 | unknown protein |
| 103147 | 10,436 | 9,002 | 0,00266 | 550,855 | unknown protein |
| 119956 | 10,41 | 6,972 | 0,00238 | 719,088 | unknown protein |
| 80335 | 10,374 | 3,911 | 0,00687 | 132,174 | unknown protein |
| 74745 | 106,865 | 5,407 | 0,00119 | 2701,265 | unknown protein |
| 5016 | 3,239 | 2,099 | 0,00508 | 196,281 | unknown protein |
| 69857 | 7,37 | 7,693 | 0,00468 | 221,298 | unknown protein |
| 112439 | 7,318 | 3,78 | 0,00487 | 210,153 | unknown protein |
| 34252 | 6,443 | 3,403 | 0,0037 | 316,916 | unknown protein |
| 109404 | 5,439 | 2,262 | 0,00535 | 182,661 | unknown protein |
| 108591 | 5,068 | 2,062 | 0,0112 | 73,302 | unknown protein |
| 54659 | 34,713 | 8,844 | 0,00276 | 520,619 | unknown protein |
| 58244 | 3,604 | 2,819 | 0,00406 | 270,835 | unknown protein |
| 48080 | 18,68 | 4,518 | 0,000884 | 11692,66 | unknown protein |
| 104584 | 2,703 | 2,758 | 0,00766 | 116,545 | unknown protein |
| 67642 | 5,37 | 5,911 | 0,00753 | 118,438 | unknown protein |
| 68566 | 5,042 | 2,907 | 0,00797 | 110,541 | unknown protein |
| 69613 | 10,16 | 9,588 | 0,00278 | 516,45 | unknown protein (Duf227) |
| 62872 | 473,2 | 15,232 | 0,00109 | 3226,832 | unknown protein GPR1/FUN34/yaaH-like |
| 53373 | 6,641 | 4,582 | 0,0015 | 1703,015 | unknown protein of CAIB/BAIF family |
| 104593 | 6,405 | 6,709 | 0,00381 | 301,691 | unknown protein with ankyrin, Leu-zipper and WD40 |
| 34197 | 2,133 | 2,334 | 0,0106 | 79,594 | unknown protein with BolA-like domain |
| 59151 | 29,897 | 2,031 | 0,00291 | 476,809 | unknown protein with BYS1 domain |
| 4862 | 2,682 | 2,163 | 0,00779 | 113,519 | unknown protein with DUF207 domain-containing protein |
| 80863 | 2,624 | 2,358 | 0,0191 | 40,335 | unknown protein with Duf895 |
| 67971 | 2,702 | 2,154 | 0,0111 | 74,224 | unknown protein with MYND zinc finger and RNA-binding domain |
| 108201 | 6,973 | 3,328 | 0,00961 | 88,345 | Unknown protein with reductase domain |
| 5614 | 4,345 | 3,477 | 0,00635 | 147,222 | unknown protein with UPF0075 domain |
| 80149 | 5,419 | 2,942 | 0,0084 | 103,617 | unknown protein, |
| 54352 | 33,965 | 2,775 | 0,00301 | 448,778 | unknown protein, |
| 66696 | 10,39 | 3,466 | 0,00232 | 739,413 | unknown protein, Duf636 |
| 120837 | 3,496 | 6,712 | 0,00368 | 318,322 | unknown protein, 1 TM domain |
| 122108 | 198,102 | 2,132 | 0,00117 | 2840,801 | unknown protein, 1TM, only in Gibberella and Chaetomium |
| 70500 | 5,455 | 4,187 | 0,00544 | 178,137 | unknown protein, 6TM |
| 123120 | 19,606 | 2,543 | 0,0108 | 77,986 | unknown protein, 8 TM |
| 110891 | 11,622 | 3,537 | 0,00262 | 572,096 | unknown protein, cupin region |
| 5359 | 4,96 | 4,165 | 0,00897 | 95,879 | unknown protein, Duf1348 |
| 106043 | 9,146 | 20,112 | 0,00336 | 372,038 | unknown protein, GFA-domain |
| 60810 | 132,265 | 60,488 | 0,00295 | 463,079 | unknown protein, GPR1/FUN34/yaaH protein, 6TMs |
| 70608 | 185,509 | 315,075 | 0,00124 | 2560,161 | unknown protein, HHE domains |
| 107525 | 3,67 | 2,045 | 0,00315 | 411,617 | unknown protein, only in fungi |
| 106676 | 4,081 | 3,271 | 0,0148 | 53,255 | unknown protein, only in Gibberella |
| 106129 | 31,766 | 13,599 | 0,00177 | 1202,977 | unknown protein, only present in ascomycota |
| 119576 | 4,347 | 2,671 | 0,00942 | 90,938 | unknown protein, only present in ascomycota and Streptomyces |
| 111887 | 35,846 | 10,007 | 0,0179 | 43,523 | unknown protein, only present in fungi |
| 42972 | 5,03 | 3,019 | 0,00961 | 88,341 | unknown protein, only present in Gibberella and Magnaporthe |
| 111138 | 8,016 | 6,453 | 0,00287 | 485,529 | unknown protein, secreted |
| 108642 | 64,054 | 75,806 | 0,00299 | 455,052 | unknown protein, secreted |
| 4454 | 6,643 | 2,852 | 0,00448 | 235,487 | unknown protein, secreted |
| 102851 | 3,724 | 3,152 | 0,0128 | 63,139 | unknown protein, secreted |
| 61642 | 10,008 | 3,011 | 0,0084 | 103,768 | unknown protein, secreted |
| 112258 | 8,938 | 8,244 | 0,00337 | 367,588 | unknown protein, unknown |
| 60482 | 7,424 | 2,582 | 0,00511 | 195,007 | unknown protein, unknown |
| 74962 | 2,128 | 2,524 | 0,0152 | 51,716 | unknown protein, WD repeats |
| 122527 | 12,086 | 7,027 | 0,00508 | 195,854 | unknown proteinwith Duf718 domain |
| 53824 | 42,078 | 3,948 | 0,00106 | 4642,885 | unknown secreted protein |
| 3462 | 2,375 | 3,497 | 0,0257 | 29,562 | UreF urease accessory protein |
| 72086 | 5,01 | 2,146 | 0,0103 | 81,818 | Vacuolar carboxypeptidase Cps1 |
| 78797 | 3,543 | 2,178 | 0,0231 | 33,166 | xanthine dehydrogenase |
| 107776 | 2,813 | 5,392 | 0,0118 | 68,963 | xylose reductase |
| 44965 | 6,789 | 7,805 | 0,0169 | 46,361 | zinc binding oxidoreductase |
| 59322 | 11,4 | 14,704 | 0,00159 | 1380,603 | Zinc binding oxidoreductase |
| 22210 | 5,431 | 2,122 | 0,00919 | 93,23 | Zinc carboxypeptidase |
| 66827 | 3,326 | 3,104 | 0,0194 | 39,761 | Zinc-containing alcohol dehydrogenase superfamily |
| 58634 | 2,599 | 2,851 | 0,0219 | 34,936 | Zn2Cys6 transcriptional regulator |
| 107858 | 2,95 | 2,431 | 0,0472 | 16,049 | Zn2Cys6 transcriptional regulator |
| 106259 | 8,276 | 8,75 | 0,00613 | 154,135 | Zn2Cys6 transcriptional regulator |
| 60282 | 7,496 | 5,279 | 0,002 | 1022,713 | Zn2Cys6 transcriptional regulator |
| 54502 | 7,461 | 3,033 | 0,00544 | 178,884 | Zn2Cys6 transcriptional regulator |
| 105784 | 7,237 | 7,33 | 0,00919 | 93,317 | Zn2Cys6 transcriptional regulator |
| 105269 | 65,591 | 8,191 | 0,00287 | 484,694 | Zn2Cys6 transcriptional regulator |
| 121474 | 4,799 | 3,041 | 0,00505 | 197,305 | Zn2Cys6 transcriptional regulator |
| 70351 | 4,633 | 7,803 | 0,0016 | 1350,204 | Zn2Cys6 transcriptional regulator |
| 123510 | 4,515 | 6,075 | 0,0149 | 52,891 | Zn2Cys6 transcriptional regulator |
| 36468 | 4,421 | 5,162 | 0,00682 | 133,849 | Zn2Cys6 transcriptional regulator |
| 71080 | 4,39 | 2,933 | 0,0132 | 60,555 | Zn2Cys6 transcriptional regulator |
| 121138 | 4,389 | 3,4 | 0,00897 | 95,634 | Zn2Cys6 transcriptional regulator |
| 103034 | 4,125 | 6,778 | 0,0195 | 39,645 | Zn2Cys6 transcriptional regulator |
| 105980 | 4,049 | 6,065 | 0,0039 | 293,61 | Zn2Cys6 transcriptional regulator |
| 72611 | 32,651 | 25,539 | 0,00302 | 450,09 | Zn2Cys6 transcriptional regulator |
| 47479 | 3,909 | 2,653 | 0,00635 | 147,563 | Zn2Cys6 transcriptional regulator |
| 106654 | 3,871 | 4,236 | 0,0114 | 71,9 | Zn2Cys6 transcriptional regulator |
| 105239 | 3,742 | 5,957 | 0,0209 | 36,742 | Zn2Cys6 transcriptional regulator |
| 69972 | 3,436 | 4,813 | 0,00368 | 319,237 | Zn2Cys6 transcriptional regulator |
| 109394 | 3,367 | 3,35 | 0,0134 | 59,42 | Zn2Cys6 transcriptional regulator |
| 80139 | 3,299 | 2,875 | 0,0261 | 29,05 | Zn2Cys6 transcriptional regulator |
| 122208 | 28,209 | 10,333 | 0,00249 | 642,864 | Zn2Cys6 transcriptional regulator |
| 112134 | 22,544 | 3,547 | 0,00674 | 135,649 | Zn2Cys6 transcriptional regulator |
| 26163 | 21,315 | 22,504 | 0,00265 | 557,054 | Zn2Cys6 transcriptional regulator |
| 112401 | 2,969 | 3,269 | 0,0346 | 21,934 | Zn2Cys6 transcriptional regulator |
| 79725 | 2,829 | 3,849 | 0,0276 | 27,46 | Zn2Cys6 transcriptional regulator |
| 105263 | 2,765 | 2,507 | 0,0174 | 44,971 | Zn2Cys6 transcriptional regulator |
| 112560 | 2,526 | 3,348 | 0,0154 | 51,112 | Zn2Cys6 transcriptional regulator |
| 112539 | 2,484 | 4,265 | 0,014 | 56,505 | Zn2Cys6 transcriptional regulator |
| 106657 | 2,313 | 2,111 | 0,0172 | 45,415 | Zn2Cys6 transcriptional regulator |
| 70414 | 2,266 | 3,182 | 0,029 | 26,196 | Zn2Cys6 transcriptional regulator |
| 111776 | 2,258 | 2,583 | 0,0149 | 52,781 | Zn2Cys6 transcriptional regulator |
| 70570 | 2,131 | 2,747 | 0,0197 | 38,961 | Zn2Cys6 transcriptional regulator |
| 53067 | 2,085 | 2,869 | 0,0128 | 63,126 | Zn2Cys6 transcriptional regulator |
| 120228 | 16,067 | 8,04 | 0,00327 | 389,532 | Zn2Cys6 transcriptional regulator |
| 65854 | 141,373 | 28,769 | 0,00522 | 188,684 | Zn2Cys6 transcriptional regulator |
| 55105 | 12,986 | 8,202 | 0,00164 | 1526,703 | Zn2Cys6 transcriptional regulator |
| 111515 | 11,596 | 7,497 | 0,00576 | 167,076 | Zn2Cys6 transcriptional regulator |
| 69695 | 11,337 | 9,308 | 0,0039 | 294,025 | Zn2Cys6 transcriptional regulator |
| 111446 | 11,117 | 7,825 | 0,00874 | 99,356 | Zn2Cys6 transcriptional regulator |
| 104380 | 10,249 | 10,343 | 0,00526 | 187,5 | Zn2Cys6 transcriptional regulator |
|  |  |  |  |  |  |
|  | **Downregulated genes** | | |  |  |
| 63751 | 2,211 | 2,071 | 0,00508 | 195,816 | 19S regulatory particle ATPase Rpt4 |
| 55644 | 3,089 | 3,492 | 0,00703 | 127,771 | 20S proteasome alpha subunit Pup2 |
| 121343 | 2,448 | 2,269 | 0,00302 | 437,584 | 20S proteasome alpha-type subunit Pre5 |
| 66707 | 2,1 | 2,093 | 0,013 | 61,406 | 20S proteasome beta-type subunit Pre3 |
| 78882 | 2,233 | 2,626 | 0,00274 | 529,573 | 20S proteasome beta-type subunit Pre7 |
| 105189 | 2,074 | 2,115 | 0,0128 | 62,946 | 20S proteasome beta-type subunit Pup1 |
| 58125 | 2,284 | 2,168 | 0,0233 | 32,806 | 20S proteasome beta-type subunit Pup3 |
| 78423 | 2,333 | 2,281 | 0,0491 | 15,397 | 26S proteasome regulatory complex subunit Rpn2 |
| 77591 | 2,634 | 2,374 | 0,0199 | 38,599 | 26S proteasome regulatory complex subunit Rpn3 |
| 54454 | 2,033 | 2,235 | 0,00512 | 194,125 | 26S proteasome regulatory complex subunit Rpn6 |
| 49923 | 2,373 | 2,671 | 0,0413 | 18,388 | 26S proteasome regulatory complex subunit Rpn7 |
| 77330 | 2,76 | 2,248 | 0,00622 | 150,983 | 26S proteasome regulatory complex subunit Rpn9 |
| 66604 | 4,562 | 7,955 | 0,0113 | 72,358 | 2-acetyl-1-alkylglycerophosphocholine esterase |
| 123431 | 2,236 | 3,155 | 0,00251 | 632,291 | 2-ketoglutarate (FeII) dependent dioxygenase |
| 3653 | 3,029 | 2,446 | 0,00617 | 152,397 | 2-oxoglutarate dehydrogenase component E2 |
| 122745 | 4,633 | 2,286 | 0,0021 | 932,442 | 2-oxoisovalerate dehydrogenase subunit beta, putative |
| 64345 | 4,275 | 2,724 | 0,021 | 36,451 | 3-Methylcrotonyl-CoA carboxylase, non-biotin containing subunit |
| 104890 | 5,644 | 2,373 | 0,00495 | 205,193 | 4-nitrophenylphosphatase |
| 104704 | 3,165 | 2,032 | 0,00749 | 119,37 | 60S ribosomal protein RML2, mitochondrial precursor from Ashbya gossypii. |
| 120272 | 3,229 | 2,604 | 0,00943 | 90,756 | 64 kDa mitochondrial NADH dehydrogenase, putative |
| 67658 | 2,3 | 2,005 | 0,00826 | 105,512 | A/G-specific adenine DNA glycosylase |
| 66551 | 5,956 | 4,602 | 0,00756 | 118,06 | AAA ATPase |
| 46128 | 4,693 | 6,235 | 0,0028 | 507,381 | AAA ATPase |
| 58366 | 199,051 | 63,593 | 0,00249 | 638,825 | AAA+-type ATPase |
| 79034 | 2,968 | 2,625 | 0,0137 | 57,909 | ABC1 family protein |
| 47926 | 3,934 | 3,564 | 0,0215 | 35,71 | ABC1 family protein |
| 65921 | 3,167 | 2,061 | 0,0433 | 17,478 | Acetyl/propionyl-CoA carboxylase alpha subunit |
| 59673 | 5,445 | 2,129 | 0,00162 | 1453,442 | acetyltransferase, GNAT family family |
| 35386 | 2,091 | 2,024 | 0,0111 | 74,905 | actin-interacting protein AIP3 |
| 123837 | 2,531 | 2,007 | 0,00374 | 311,966 | actin-like protein, centractin |
| 75704 | 14,69 | 3,237 | 0,00148 | 1773,447 | Adenosine 5'-phosphosulfate kinase |
| 109187 | 3,363 | 2,213 | 0,0168 | 46,879 | alanine transaminase-like protein |
| 69956 | 20,164 | 2,915 | 0,00159 | 1439,012 | Alcohol dehydrogenase, class V |
| 23382 | 24,352 | 6,148 | 0,00212 | 939,014 | aldehyde reductase AKR7 |
| 55351 | 2,803 | 2,776 | 0,0278 | 27,272 | Alkyl hydroperoxide reductase/peroxiredoxin |
| 57940 | 2,627 | 2,317 | 0,0053 | 185,444 | alternative oxidase aox1 |
| 69863 | 2,679 | 2,461 | 0,0162 | 48,586 | Amidase |
| 78072 | 2,343 | 2,212 | 0,0343 | 22,177 | Amidase |
| 109378 | 3,461 | 2,754 | 0,011 | 75,848 | Amidases |
| 82014 | 38,598 | 2,654 | 0,00141 | 2086,631 | Amino acid permease |
| 67806 | 6,291 | 2,193 | 0,0107 | 78,391 | Amino acid permease |
| 54365 | 2,803 | 2,361 | 0,0128 | 62,723 | Amino acid transporter |
| 68831 | 86,765 | 2,624 | 0,00281 | 502,161 | amino acid transporter |
| 123718 | 628,761 | 496,845 | 0,00107 | 3768,766 | amino acid transporter, neutral 11 TM |
| 110316 | 718,275 | 48,075 | 0,00338 | 365,911 | Amino acid transporters |
| 121139 | 29,249 | 5,688 | 0,0025 | 630,94 | Amino acid transporters |
| 43671 | 22,897 | 16,919 | 0,002 | 1019,785 | ammonium permease MEA1 |
| 82095 | 5,157 | 3,13 | 0,0214 | 35,816 | ammonium transporter, high affinity |
| 82204 | 14,408 | 4,517 | 0,003 | 453,216 | ammonium transporter, high affinity |
| 77811 | 3,037 | 2,56 | 0,0197 | 38,96 | AMP-dependent synthetase and ligase |
| 75859 | 2,898 | 2,133 | 0,0283 | 26,828 | anion-transporting ATPase |
| 106314 | 9,735 | 4,035 | 0,00875 | 99,104 | Ankyrin |
| 63687 | 6,878 | 8,348 | 0,00184 | 1125,926 | ankyrin |
| 60773 | 2,839 | 6,539 | 0,00775 | 115,069 | ankyrin |
| 52222 | 3,866 | 7,736 | 0,00721 | 124,302 | ankyrin repeat domain-containing protein |
| 5233 | 2,182 | 2,025 | 0,00164 | 1517,421 | aspartate/other aminotransferase |
| 74156 | 89,063 | 4,354 | 0,00113 | 3997,421 | aspartyl protease |
| 122076 | 2,959 | 8,554 | 0,00261 | 584,977 | aspartyl protease |
| 108686 | 2,098 | 4,077 | 0,0112 | 73,589 | Aspartyl protease |
| 48819 | 3,828 | 4,318 | 0,00527 | 187,011 | aspartyl-tRNA synthetase, class IIb. |
| 81576 | 45,898 | 12,741 | 0,00262 | 563,253 | assimilatory sulfite reductase, alpha subunit |
| 74892 | 3,141 | 2,769 | 0,00292 | 470,859 | ATP synthase regulation protein NCA2, putative |
| 22885 | 5,151 | 2,526 | 0,00486 | 210,982 | ATP11 protein |
| 121248 | 2,64 | 2,061 | 0,0077 | 115,967 | ATP12 chaperone , putative |
| 3579 | 4,806 | 3,831 | 0,00559 | 172,5 | ATP-dependent RNA helicases. |
| 4767 | 4,787 | 4,272 | 0,0135 | 58,865 | biotin synthase |
| 72780 | 12,526 | 8,103 | 0,011 | 75,734 | bZIP transcription factor |
| 107974 | 2,336 | 87,4 | 0,00261 | 596,09 | bZIP transcriptional regulator transcription factor JlbA/IDI-4 |
| 75890 | 4,72 | 2,082 | 0,0111 | 75,395 | C1 tetrahydrofolate synthase, putative |
| 122448 | 12,063 | 8,456 | 0,00158 | 1379,117 | C2H2 transcription factor |
| 39637 | 2,187 | 5,115 | 0,00233 | 736,988 | C4-dicarboxylate transporter/malic acid transport protein |
| 107073 | 9,816 | 3,454 | 0,00802 | 109,899 | Ca2+/calmodulin-dependent protein kinase, EF-Hand protein superfamily |
| 119707 | 2,201 | 3,136 | 0,0402 | 18,884 | calponin-like actin binding domain |
| 55049 | 4,654 | 2,631 | 0,00227 | 774,099 | casein kinase 1 delta |
| 80672 | 2,943 | 2,91 | 0,0285 | 26,588 | cation efflux family protein, putative |
| 21181 | 2,649 | 2,624 | 0,0149 | 52,995 | cell division cycle 37 protein, CDC37 |
| 31481 | 2,306 | 2,19 | 0,00474 | 217,317 | chaperone protein dnaJ 6 |
| 57287 | 2,148 | 2,176 | 0,0181 | 42,97 | chitin synthase activator, putative |
| 123084 | 54,473 | 45,916 | 0,000822 | 9809,57 | chloroperoxidase |
| 68950 | 3,039 | 4,284 | 0,0043 | 249,464 | chloroperoxidase |
| 69696 | 238,3 | 7,009 | 0,00204 | 979,271 | [coenzyme F420-dependent N5,N10-methylene tetrahydromethanopterin reductase](http://www.genome.jp/dbget-bin/www_bget?aor:AO090124000018) |
| 60136 | 2,062 | 2,01 | 0,0405 | 18,716 | COPII coat assembly protein Sec16 |
| 108749 | 13,665 | 13,4 | 0,00162 | 1560,267 | Copper transporter |
| 52315 | 1756,734 | 1966,144 | 0,00111 | 3278,897 | copper transporter |
| 62716 | 1586,692 | 1452,16 | 0,000904 | 11036,16 | copper transporter family protein |
| 71029 | 8,013 | 10,008 | 0,0033 | 381,719 | copper transporter, putative |
| 123382 | 2,268 | 3,427 | 0,00404 | 273,055 | Coproporphyrinogen III oxidase |
| 105449 | 2,1 | 4,99 | 0,0111 | 74,902 | Cyclin C-dependent kinase CDK8 |
| 81089 | 8,46 | 5,647 | 0,00236 | 725,868 | CysK, Cysteine synthase; aa370-414 cd02205, CBS domain |
| 3641 | 5,812 | 2,791 | 0,0023 | 751,68 | Cystathionine beta-lyases/cystathionine gamma-synthases |
| 69605 | 2,578 | 2,285 | 0,0375 | 20,22 | Cystathionine beta-lyases/cystathionine gamma-synthases |
| 77955 | 2,228 | 2,253 | 0,025 | 30,386 | cytochrome b5-like Heme/Steroid binding domain-containing protein |
| 23431 | 2,796 | 2,617 | 0,0275 | 27,602 | Cytochrome bd ubiquinol oxidase, 14 kDa subunit |
| 51130 | 4,102 | 3,275 | 0,0179 | 43,553 | cytochrome c |
| 77758 | 5,34 | 3,348 | 0,00307 | 426,212 | cytochrome c heme lyase, putative |
| 4668 | 3,177 | 2,893 | 0,0102 | 83,166 | cytochrome c oxidase assembly protein |
| 54676 | 8,416 | 6,384 | 0,0165 | 47,75 | cytochrome C oxidase assembly protein COX17 |
| 80932 | 3,198 | 2,013 | 0,00799 | 110,205 | Cytochrome c oxidase assembly protein CtaG/Cox11 |
| 49205 | 3,481 | 2,488 | 0,0201 | 38,194 | cytochrome C peroxidase |
| 109811 | 2,125 | 2,078 | 0,0169 | 46,291 | Cytochrome P450 |
| 62231 | 7,84 | 4,469 | 0,0139 | 57,097 | Cytochrome P450 CYP3/CYP5/CYP6/CYP9 subfamilies |
| 78223 | 14,091 | 4,74 | 0,00239 | 703,531 | Cytochrome P450 CYP3/CYP5/CYP6/CYP9 subfamilies |
| 4517 | 59,004 | 73,042 | 0,00161 | 1492,829 | Cytochrome P450 CYP4/CYP19/CYP26 subfamilies |
| 64869 | 3,84 | 8,97 | 0,0104 | 80,766 | Cytochrome P450 CYP4/CYP19/CYP26 subfamilies |
| 65141 | 7,541 | 2,719 | 0,00424 | 254,854 | cytochrome P450 monooxygenase |
| 111049 | 6,209 | 4,219 | 0,00339 | 364,305 | cytochrome P450 protein, class IV |
| 52489 | 6,548 | 4,448 | 0,00439 | 243,103 | cytosin/purin permease |
| 120064 | 4,408 | 2,211 | 0,00162 | 1414,792 | DEAD box helicase Hel1 |
| 57868 | 3,665 | 2,203 | 0,0165 | 47,846 | Delta 1-pyrroline-5-carboxylate reductase |
| 63742 | 2,384 | 2,479 | 0,00962 | 88,354 | Demethoxyubiquinone hydroxylase |
| 75226 | 4,03 | 2,167 | 0,00694 | 130,577 | dihydroorotate dehydrogenase |
| 59508 | 3,695 | 4,149 | 0,00992 | 85,324 | dipeptidyl peptidase 5 |
| 74943 | 2,994 | 2,223 | 0,016 | 49,19 | Diphthine synthase |
| 110362 | 2,689 | 2,42 | 0,0218 | 35,134 | DNA polymerase gamma, the mitochondrial replicative DNA polymerase |
| 105676 | 3,495 | 2,981 | 0,00712 | 125,78 | DNA replication licensing factor mcm7 |
| 59876 | 317,15 | 4,25 | 0,00246 | 670,981 | DszA-like P540-dependent xenobiotic monoxygenase |
| 111564 | 3,941 | 2,416 | 0,00338 | 363,714 | D-tyrosyl-tRNA deacylase |
| 77481 | 2,04 | 2,783 | 0,00201 | 1000,999 | D-xylulose 5-phosphate/D-fructose 6-phosphate phosphoketolase |
| 79690 | 2,794 | 2,089 | 0,00535 | 182,88 | Electron transfer flavoprotein-ubiquinone oxidoreductase |
| 123588 | 5,506 | 3,119 | 0,00688 | 131,745 | electron transport protein, probably involved in cytochrome C assembly |
| 123131 | 4,48 | 3,501 | 0,0165 | 47,818 | endonuclease/exonuclease/phosphatase, putative |
| 61442 | 2,826 | 2,723 | 0,0158 | 49,734 | enoyl-CoA hydratase/isomerase |
| 79741 | 26,586 | 5,9 | 0,00735 | 121,942 | ER-bound Farnesyl-diphosphate farnesyltransferase |
| 111837 | 2,04 | 4,464 | 0,00217 | 877,645 | Esterase/lipase/thioesterase superfamily |
| 106405 | 2,339 | 2,236 | 0,00204 | 972,896 | extracellular lipase-like protein |
| 109201 | 2,201 | 8,101 | 0,0128 | 62,955 | FAD binding domain-containing protein |
| 58333 | 4,215 | 3,128 | 0,00231 | 745,211 | FAD binding protein |
| 60784 | 6,526 | 2,055 | 0,00915 | 93,725 | FAD binding protein |
| 69650 | 21,5 | 59,058 | 0,00263 | 570,111 | FAD binding protein |
| 1751 | 2,886 | 3,963 | 0,00437 | 244,953 | FAD monooxygenase |
| 60849 | 20,334 | 11,153 | 0,00301 | 443,545 | FAD-dependent sulfhydryl oxidase Erv1 |
| 54144 | 8,892 | 2,656 | 0,00574 | 167,392 | ferric reductase |
| 22845 | 5,831 | 5,997 | 0,00653 | 141,737 | ferric reductase |
| 111750 | 23,153 | 23,115 | 0,00131 | 2306,795 | ferric reductase |
| 23353 | 1853,847 | 1794,549 | 0,000877 | 6327,492 | ferric reductase |
| 81096 | 12,439 | 12,972 | 0,00116 | 4275,83 | ferric reductase |
| 5119 | 72,437 | 79,895 | 0,00327 | 387,941 | ferrooxidoreductase |
| 22110 | 35,624 | 2,2 | 0,00303 | 433,192 | Flavin-containing monooxygenase |
| 111681 | 2,953 | 2,944 | 0,0108 | 77,792 | Flavin-containing monooxygenase |
| 74351 | 2,031 | 2,111 | 0,0108 | 77,293 | Flavin-containing monooxygenase Fmo1 |
| 123251 | 2,9 | 4,048 | 0,0124 | 65,523 | flavohemoglobin |
| 76722 | 2,273 | 3,891 | 0,00708 | 126,725 | flavohemoglobin |
| 70311 | 4,143 | 3,634 | 0,00569 | 168,932 | flavoprotein monooxygenase |
| 73623 | 1986,095 | 1234,879 | 0,00112 | 3889,996 | flavoprotein monooxygenase |
| 74122 | 7,908 | 4,361 | 0,00906 | 94,903 | Galactokinase |
| 124158 | 3,391 | 2,307 | 0,0103 | 82,001 | GCN5 N-acetyltransferase |
| 106798 | 2,678 | 8,254 | 0,00373 | 314,37 | GCN5-N-acetyltransferase |
| 78864 | 4,295 | 2,007 | 0,00991 | 85,219 | GcvT Glycine cleavage system T protein |
| 68022 | 2,882 | 2,543 | 0,0198 | 38,778 | geranylgeranyl diphosphate synthase, related to N. crassa albino-3 |
| 61703 | 2,248 | 2,214 | 0,0256 | 29,689 | germinal center kinase, related to S. cerevisiae Kic1 |
| 73101 | 2,301 | 2,143 | 0,0452 | 16,729 | GH16 endo-ß-1,3/1,4-glucanase |
| 49274 | 19,08 | 11,25 | 0,00582 | 164,25 | GH16 endo-ß-1,3/1,4-glucanase |
| 65406 | 2,09 | 3,055 | 0,00416 | 264,11 | GH16 endo-ß-1,3/1,4-glucanase |
| 82633 | 3,294 | 5,709 | 0,0018 | 1162,841 | GH2 ß-1,3-glucanosyltransferase Gel3 |
| 109278 | 2,481 | 3,232 | 0,00772 | 115,497 | GH24 lysozyme |
| 70186 | 7,605 | 7,397 | 0,000328 | 137976,1 | GH28 polygalacturonase |
| 3094 | 2,878 | 5,938 | 0,00787 | 112,012 | GH30 glucuronyl-xylanase |
| 64375 | 2,419 | 2,449 | 0,0195 | 39,353 | GH5 exo-ß-1,3-glucanase |
| 54242 | 14,048 | 35,509 | 0,00301 | 447,504 | GH55 ß-1,3-glucanase |
| 123639 | 17,698 | 9,893 | 0,00419 | 259,496 | GH64 ß-1,3-glucanase |
| 121664 | 10,782 | 3,263 | 0,00565 | 169,904 | Glutamate decarboxylase |
| 124332 | 2,84 | 2,147 | 0,00806 | 108,466 | glutamyl-tRNA synthetase, class Ic. |
| 47136 | 4,69 | 3,563 | 0,0123 | 65,934 | Glutathione peroxidase |
| 111082 | 11,895 | 8,214 | 0,00119 | 2953,275 | glutathione S transferase, 2 TM |
| 112633 | 6,661 | 2,001 | 0,00389 | 293,892 | glutathione S-transferase |
| 22453 | 20,118 | 4,809 | 0,00354 | 342,276 | glutathione-S-transferase |
| 2574 | 2,177 | 2,006 | 0,00915 | 93,755 | glycerol-3-phosphate dehydrogenase, NAD-dependent |
| 57357 | 4,601 | 2,933 | 0,0207 | 37,221 | Glycine cleavage T protein (aminomethyl transferase), putative |
| 66795 | 351,932 | 4,163 | 0,00315 | 414,013 | GMC oxidoreductase family protein |
| 27948 | 2,294 | 2,549 | 0,00574 | 167,703 | GPCR, rhodopsin type |
| 72788 | 2,975 | 3,322 | 0,0164 | 48,183 | GT 31 glycosyltransferase |
| 120923 | 3,549 | 176,411 | 0,00286 | 491,087 | GT 32 |
| 77547 | 2,64 | 2,285 | 0,00314 | 413,596 | GT1 glycosyltransferase |
| 56638 | 2,888 | 3,462 | 0,00498 | 201,403 | GT64 alpha-N-acetylhexosaminyltransferases |
| 62377 | 4,619 | 6,053 | 0,00425 | 252,838 | GT64 a-N-acetylhexosaminyltransferases |
| 59542 | 2,005 | 2,949 | 0,0247 | 30,761 | Guanyl-nucleotide exchange factor Sec2 |
| 68444 | 2,99 | 2,662 | 0,00489 | 209,106 | haloacid dehalogenase-like hydrolase, putative |
| 41660 | 3,035 | 8,661 | 0,0108 | 77,446 | HET domain protein |
| 112083 | 6,797 | 5,884 | 0,00437 | 244,965 | HET protein |
| 123733 | 6,014 | 10,096 | 0,00339 | 361,644 | HET protein |
| 103189 | 2,696 | 3,774 | 0,0171 | 45,741 | HET protein |
| 109101 | 8,59 | 12,034 | 0,00328 | 386,658 | HET protein |
| 103065 | 2,539 | 16,705 | 0,00247 | 654,835 | HET protein |
| 107071 | 12,324 | 2,218 | 0,00515 | 192,391 | HET-domain protein, unknown unknown |
| 68728 | 3,44 | 2,18 | 0,0155 | 50,668 | hexaprenyl pyrophosphate synthase |
| 105869 | 11,307 | 6,594 | 0,00432 | 248,211 | HFB, class II hydrophobin |
| 81188 | 2,657 | 2,075 | 0,00293 | 467,365 | HisF Imidazoleglycerol-phosphate synthase |
| 61081 | 2,495 | 2,372 | 0,0419 | 18,112 | histone acetyltransferase SPT10 |
| 120786 | 2,17 | 2,045 | 0,0435 | 17,416 | HMGL-like |
| 58853 | 16,353 | 16,765 | 0,00286 | 489,198 | Homeodomain-like |
| 123468 | 9,888 | 5,014 | 0,00231 | 753,879 | IMP dehydrogenase |
| 104161 | 4,84 | 3,924 | 0,0118 | 69,2 | importin beta-5 subunit, putative |
| 77552 | 18,147 | 38,809 | 0,00328 | 385,289 | Inorganic phosphate transporter |
| 102559 | 3,661 | 2,369 | 0,00873 | 99,468 | integral membrane protein |
| 80639 | 46,463 | 69,71 | 0,0045 | 233,885 | iron transporter |
| 67217 | 2,623 | 3,455 | 0,00586 | 162,482 | iron-sulfur cluster assembly accessory protein Isa1, putative |
| 73631 | 166,217 | 100,128 | 0,000945 | 12303,86 | isoamyl alcohol oxidase |
| 62359 | 188,457 | 80,042 | 0,00118 | 2848,535 | isoflavon reductase |
| 110276 | 5,229 | 3,952 | 0,00919 | 93,334 | isoflavone reductase |
| 81525 | 19,159 | 6,699 | 0,00211 | 927,927 | isoflavone reductase |
| 122010 | 2,968 | 3,021 | 0,0065 | 143,491 | Lactate dehydrogenase |
| 22351 | 4,43 | 3,585 | 0,0121 | 67,476 | legume-like lectin |
| 77795 | 3,456 | 2,148 | 0,0029 | 478,468 | LIMPET, E3-ubiquitin ligase SCON-2 |
| 81362 | 6,716 | 2,753 | 0,00543 | 179,546 | Lipoate synthase |
| 25040 | 2,103 | 2,15 | 0,0387 | 19,581 | Lrg1 which is highly expressed in sporulation |
| 111600 | 2,673 | 3,946 | 0,0103 | 82,345 | lysosomal cystine transporter |
| 62153 | 2,634 | 7,511 | 0,00161 | 1401,034 | malate dehydrogenase |
| 76633 | 5,107 | 5,726 | 0,00585 | 162,968 | MDR multidrug transporter |
| 123293 | 3,909 | 2,242 | 0,0126 | 64,553 | MDR-type ABC transporters |
| 60425 | 3,447 | 3,99 | 0,00328 | 383,108 | mechanosensitive ion channel family |
| 111121 | 42,808 | 359,603 | 0,00192 | 1068,354 | metallopeptidase |
| 2433 | 10,893 | 3,833 | 0,00672 | 135,973 | methyltransferase, putative |
| 76800 | 2,911 | 18,635 | 0,00416 | 264,228 | MFS transporter |
| 82280 | 3,642 | 2,058 | 0,00661 | 139,136 | mitochondrial 2-oxoglutarate/2-oxoadipate transporter-like protein |
| 54064 | 5,592 | 2,182 | 0,0105 | 80,178 | mitochondrial ATP-dependent RNA-helicase mrh4 |
| 23295 | 4,405 | 2,555 | 0,00227 | 785,171 | mitochondrial carrier protein |
| 80332 | 2,527 | 2,075 | 0,00266 | 551,33 | mitochondrial carrier protein, putative |
| 56467 | 6,625 | 3,682 | 0,00626 | 149,644 | Mitochondrial deoxynucleotide carrier protein |
| 62470 | 3,292 | 2,177 | 0,0088 | 98,028 | mitochondrial elongation factor G. |
| 60847 | 4,14 | 2,608 | 0,00558 | 171,747 | Mitochondrial F1F0-ATP synthase, subunit c/ATP9/proteolipid |
| 121436 | 6,369 | 2,365 | 0,00686 | 132,374 | Mitochondrial inner membrane insertase |
| 81822 | 2,917 | 3,874 | 0,0128 | 62,645 | mitochondrial leucyl-tRNA synthetase. |
| 60406 | 5,52 | 2,41 | 0,00797 | 110,646 | mitochondrial matrix iron-sulfur protein |
| 61794 | 4,172 | 2,696 | 0,00529 | 186,002 | mitochondrial nuclease |
| 78836 | 3,298 | 2,22 | 0,00803 | 109,45 | mitochondrial phosphate carrier |
| 77122 | 7,928 | 3,838 | 0,00853 | 101,883 | mitochondrial presequence protease CYM1 |
| 102593 | 4,133 | 2,616 | 0,0112 | 73,018 | Mitochondrial ribosomal protein L17 |
| 3909 | 3,568 | 3,421 | 0,0128 | 62,629 | Mitochondrial ribosomal protein L43 |
| 123695 | 2,988 | 2,298 | 0,0487 | 15,553 | mitochondrial ribosomal protein MRPL11. |
| 63269 | 3,489 | 2,091 | 0,0109 | 76,939 | mitochondrial ribosomal protein MRPL3. |
| 67366 | 2,554 | 2,017 | 0,00707 | 126,932 | mitochondrial ribosomal protein MRPL49. |
| 31611 | 7,133 | 3,519 | 0,00248 | 647,507 | mitrochondrial inner membrane precurser translocase TIM40 |
| 66345 | 6,678 | 7,111 | 0,00321 | 401,414 | Mn superoxide dismutase |
| 56176 | 3,813 | 2,259 | 0,00644 | 145,265 | MRP-type ABC transporter |
| 67732 | 2,48 | 4,527 | 0,0123 | 66,192 | MRP-type ABC transporter |
| 80879 | 81,861 | 45,529 | 0,00322 | 401,732 | MSF H+/oligopeptide transporter |
| 43701 | 101,205 | 97,534 | 0,00162 | 1401,307 | MSF multidrug transporter |
| 80086 | 15,511 | 2,062 | 0,0126 | 64,286 | MSF peptide transporter |
| 76641 | 9,07 | 3,534 | 0,00355 | 336,274 | MSF permease |
| 123702 | 19,973 | 2,819 | 0,00323 | 395,229 | MSF permease |
| 107936 | 6,768 | 4,294 | 0,00432 | 247,922 | MSF transporter |
| 62747 | 5,947 | 6,619 | 0,00328 | 383,398 | MSF transporter |
| 65583 | 5,473 | 3,043 | 0,00187 | 1096,225 | MSF transporter |
| 122948 | 2,515 | 2,521 | 0,00829 | 105,157 | multicopper oxidase |
| 67107 | 2,47 | 2,746 | 0,00304 | 430,759 | MutS-related protein involved in mismatch repair |
| 69656 | 2,704 | 8,781 | 0,00338 | 363,556 | MYND zinc finger protein |
| 106928 | 18,075 | 38,036 | 0,00119 | 2728,823 | Na/K ATPase alpha 1 subunit, |
| 120231 | 3,098 | 5,871 | 0,0113 | 72,41 | NACHT domain WD40 repeat-containing protein |
| 3327 | 11,032 | 5,963 | 0,00482 | 213,318 | NADH:flavin oxidoreductase/NADH oxidase |
| 122416 | 5,039 | 2,485 | 0,013 | 61,727 | NADH:flavin oxidoreductase/NADH oxidase |
| 123999 | 4,223 | 4,373 | 0,0092 | 93,25 | NADH:flavin oxidoreductase/NADH oxidase |
| 69769 | 8,102 | 4,308 | 0,00472 | 218,839 | NADH:flavin oxidoreductase/NADH oxidase, putative |
| 79334 | 41,333 | 31,68 | 0,00627 | 149,764 | NADH-cytochrome b5 reductase, putative |
| 34327 | 2,134 | 2,17 | 0,0118 | 69,398 | NADH-ubiquinone oxidoreductase B12 subunit, putative |
| 122641 | 5,305 | 3,117 | 0,0165 | 47,87 | nicotinate phosphoribosyltransferase activity |
| 59672 | 7,256 | 2,334 | 0,00325 | 393,808 | nitroreductase family protein |
| 122767 | 2,009 | 2,058 | 0,00234 | 733,403 | N-terminal cooper fist DNA-binding domain-containing protein |
| 60346 | 5,39 | 2,612 | 0,00877 | 98,783 | Para-aminobenzoate (PABA) synthase PabaA |
| 77703 | 7,631 | 2,092 | 0,00681 | 133,274 | PDR-type ABC transporters |
| 59014 | 3,961 | 2,401 | 0,0184 | 42,108 | PDR-type ABC transporters |
| 47127 | 3,173 | 4,202 | 0,00108 | 3469,264 | peptidase M18 |
| 103039 | 30,068 | 2,064 | 0,00242 | 706,192 | peptidase S41 |
| 111451 | 10,838 | 10,738 | 0,00806 | 108,975 | peptidase S41 |
| 78264 | 8,723 | 4,131 | 0,0041 | 268,487 | Peptide methionine sulfoxide reductase |
| 53342 | 4,507 | 3,948 | 0,015 | 52,452 | Peroxin-3 |
| 59070 | 4,969 | 4,158 | 0,00652 | 142,758 | peroxisome biosynthesis protein (PAS1/Peroxin-1), |
| 65410 | 12,926 | 3,953 | 0,00211 | 936,901 | Phosphoadenosine phosphosulfate reductase |
| 77656 | 3,417 | 2,074 | 0,00646 | 144,561 | phosphoglycerate mutase |
| 57975 | 2,058 | 2,29 | 0,0365 | 20,791 | phospholipase C, related to Aspergillus fumigatus phosphatidylinositol phospholipase C |
| 75169 | 4,544 | 2,925 | 0,00217 | 880,638 | phosphoserine phosphatase |
| 73621 | 255,424 | 117,144 | 0,00142 | 2031,74 | PKS |
| 73618 | 139,697 | 54,849 | 0,000743 | 8873,051 | PKS |
| 104079 | 2,681 | 12,187 | 0,0171 | 45,809 | plant expansins, EXPN |
| 110890 | 3,392 | 2,116 | 0,0015 | 1849,181 | porphobilinogen deaminase-like protein |
| 46545 | 2,816 | 3,64 | 0,0175 | 44,646 | Prephenate dehydrogenase |
| 103625 | 2,425 | 2,67 | 0,0147 | 53,695 | proteasome maturation factor UMP1, putative |
| 68364 | 9,474 | 6 | 0,00136 | 2214,294 | Protein kinase |
| 27025 | 2,286 | 2,719 | 0,0156 | 50,501 | Protein kinase |
| 47290 | 2,163 | 2,783 | 0,00776 | 114,146 | protein palmitoyl transferase PFA3 |
| 66702 | 3,146 | 2,792 | 0,0207 | 37,155 | Protein phosphatase 2C-like |
| 122824 | 67,394 | 3,986 | 0,00248 | 650,912 | PTH11 GPCR |
| 122795 | 4,136 | 3,452 | 0,0124 | 65,393 | PTH11 GPCR |
| 76763 | 13,486 | 7,007 | 0,0183 | 42,465 | PTH11 GPCR |
| 66616 | 39,559 | 2,864 | 0,00446 | 237,001 | Purple acid phosphatase |
| 121534 | 2,45 | 2,955 | 0,00512 | 194,381 | pyruvate decarboxylase |
| 72259 | 2,148 | 6,314 | 0,00581 | 165,271 | RgsA, regulator of G-protein signaling |
| 53562 | 3,777 | 3,228 | 0,00281 | 504,289 | rho4 |
| 110655 | 2,492 | 5,066 | 0,00226 | 770,697 | RhoA GTPase effector arrestin |
| 82560 | 2,217 | 2,455 | 0,0474 | 15,979 | Ribokinase |
| 23059 | 2,476 | 2,443 | 0,048 | 15,765 | ribosomal protein L24/L26 |
| 4284 | 3,732 | 2,645 | 0,0139 | 56,811 | ribosomal protein L3 |
| 77932 | 2,03 | 2,227 | 0,0452 | 16,724 | ribosomal protein L30. |
| 31976 | 8,572 | 5,721 | 0,0138 | 57,33 | ribosomal protein L6 |
| 110295 | 2,603 | 2,413 | 0,045 | 16,832 | ribosomal protein S14 |
| 78661 | 3,072 | 2,116 | 0,0401 | 18,933 | ribosomal protein S5, MRPS5. |
| 76740 | 3,175 | 2,479 | 0,0193 | 39,998 | ribosome associated DnaJ chaperone Zuotin |
| 22689 | 2,292 | 2,033 | 0,0024 | 700,037 | ribosome biogenesis protein Pescadillo, putative |
| 38372 | 2,455 | 2,335 | 0,0173 | 45,098 | RluA family pseudouridine synthase |
| 80056 | 3,058 | 6,295 | 0,00466 | 222,422 | RNA 3'-terminal phosphate cyclase , putative |
| 55599 | 2,436 | 2,335 | 0,0252 | 30,222 | RNA12, putative |
| 110171 | 4,612 | 2,089 | 0,00629 | 148,854 | S-adenosyl-L-homocysteine hydrolase |
| 68348 | 2,714 | 5,766 | 0,00344 | 354,704 | SAM-dependent methyltransferase |
| 105242 | 75,229 | 20,968 | 0,00109 | 3386,644 | SAM-dependent methyltransferases |
| 69742 | 35,262 | 3,795 | 0,00215 | 908,899 | secreted esterase/lipase |
| 64167 | 89,906 | 3,539 | 0,00118 | 2942,021 | Sexual differentiation process protein ISP4 |
| 70520 | 52,084 | 41,005 | 0,00161 | 1393,269 | short chain dehydrogenase/reductase |
| 59624 | 3,616 | 5,323 | 0,00392 | 285,553 | short chain dehydrogenase/reductase |
| 60033 | 3,241 | 2,572 | 0,00447 | 236,772 | short chain dehydrogenase/reductase |
| 111966 | 21,536 | 10,54 | 0,00222 | 818,134 | short chain dehydrogenase/reductase |
| 58675 | 2,782 | 2,389 | 0,0168 | 46,803 | short chain dehydrogenase/reductase |
| 59196 | 15,013 | 2,483 | 0,00394 | 284,047 | short chain dehydrogenase/reductase |
| 60518 | 24,268 | 12,09 | 0,00246 | 657,482 | short chain-type dehydrogenase/reductase |
| 111417 | 9,29 | 2,624 | 0,00682 | 133,09 | short-chain alcohol dehydrogenases |
| 112201 | 8,701 | 3,847 | 0,00415 | 263,988 | Short-chain dehydrogenase/reductase |
| 40808 | 5,441 | 5,751 | 0,00992 | 85,236 | short-chain dehydrogenase/reductase |
| 105888 | 4,441 | 5,197 | 0,0157 | 49,885 | Short-chain dehydrogenase/reductase SDR |
| 103113 | 37,899 | 4,546 | 0,00216 | 891,524 | Short-chain dehydrogenase/reductase SDR |
| 22277 | 5,043 | 2,006 | 0,00465 | 224,43 | Sideroflexin (mitochindrial tricarboxylate/Fe carrier) |
| 50268 | 2,393 | 2,678 | 0,00493 | 204,665 | sir2 family histone deacetylase |
| 62522 | 11,551 | 2,039 | 0,00427 | 251,565 | small, cystein-rich protein, secreted |
| 5350 | 2,509 | 3,82 | 0,0193 | 39,882 | SRP receptor, beta subunit |
| 81690 | 2,961 | 2,453 | 0,00819 | 106,658 | ß-arrestin protein, shares similarity with Aspergillus idulans CreD, possible inhibitor of G-protein coupled receptors |
| 29709 | 3,589 | 2,457 | 0,00664 | 138,396 | stomatin family protein |
| 47066 | 8,259 | 2,943 | 0,00696 | 129,984 | sulfate adenylyltransferase |
| 122943 | 3,129 | 3,964 | 0,0123 | 66,329 | SWI-SNF chromatin-remodeling complex protein |
| 111874 | 4,429 | 5,206 | 0,0106 | 79,732 | swollenin 2 |
| 4514 | 2,273 | 2,326 | 0,00275 | 523,119 | tetraspanin |
| 79565 | 5,787 | 3,311 | 0,00699 | 129,419 | Thioredoxin reductase TrxB |
| 104135 | 2,855 | 2,072 | 0,00696 | 129,873 | Thioredoxin, putative |
| 52924 | 2,284 | 2,253 | 0,0287 | 26,479 | transcriptional activator, zinc finger, NF-X1-type |
| 27649 | 2,549 | 2,229 | 0,00513 | 193,629 | transcriptional regulator Rme1, repressor of the meiosis regulator protein IME1 |
| 67907 | 3,2 | 8,845 | 0,0124 | 65,468 | transfer of mannosylphosphate |
| 31798 | 2,084 | 3,701 | 0,00645 | 144,769 | transfer of mannosylphosphate |
| 122909 | 2,487 | 2,452 | 0,00802 | 109,857 | translation elongation factor eEF-3 |
| 82623 | 422,807 | 8,305 | 0,000906 | 5886,798 | tripeptide peptidase |
| 58267 | 3,147 | 2,61 | 0,0138 | 57,348 | tRNA-dihydrouridine synthase. |
| 63754 | 2,168 | 2,081 | 0,0123 | 66,406 | t-SNARE, SSO1 |
| 50793 | 5,439 | 6,053 | 0,0261 | 29,072 | tyrosinase |
| 71759 | 5,456 | 2,677 | 0,0028 | 508,395 | tyrosyl-tRNA synthetase, class Ib. |
| 82334 | 2,685 | 2,063 | 0,00493 | 203,747 | tyrosyl-tRNA synthetase, class Ib. |
| 103321 | 2,339 | 2,148 | 0,0406 | 18,698 | U4/U6.U5 tri-snRNP-associated protein |
| 102581 | 2,378 | 2,52 | 0,0144 | 54,761 | Ubiquitin binding protein Rad23 |
| 80762 | 2,729 | 2,736 | 0,00495 | 205,703 | ubiquitin carboxyl-terminal hydrolase, family 1 |
| 80400 | 2,349 | 2,43 | 0,0421 | 18,008 | Ubiquitin C-terminal hydrolase |
| 59705 | 2,239 | 2,563 | 0,0327 | 23,317 | UBX domain-containing protein |
| 77227 | 3,538 | 3,042 | 0,00639 | 146,508 | UDP-glucose ceramide glucosyltransferase |
| 120879 | 5,311 | 8,269 | 0,00346 | 350,945 | unique protein |
| 110363 | 9,629 | 3,237 | 0,00615 | 153,623 | unique protein |
| 105313 | 8,665 | 41,937 | 0,00532 | 184,173 | unique protein |
| 108232 | 621,561 | 262,19 | 0,00196 | 1047,62 | unique protein |
| 121336 | 5,968 | 3,805 | 0,00807 | 108,809 | unique protein |
| 110880 | 5,916 | 3,942 | 0,0195 | 39,57 | unique protein |
| 122506 | 5,467 | 18,263 | 0,00379 | 305,333 | unique protein |
| 120362 | 4,807 | 14,488 | 0,00149 | 1738,053 | unique protein |
| 104173 | 4,772 | 9,711 | 0,00648 | 143,939 | unique protein |
| 111137 | 4,209 | 3,343 | 0,0106 | 79,5 | unique protein |
| 110127 | 34,018 | 97,557 | 0,00442 | 240,977 | unique protein |
| 121136 | 31,18 | 247,126 | 0,0016 | 1386,455 | unique protein |
| 102787 | 2,498 | 6,622 | 0,00958 | 88,903 | unique protein |
| 109945 | 2,485 | 19,352 | 0,00114 | 3933,826 | unique protein |
| 111122 | 19,034 | 136,683 | 0,00182 | 1141,894 | unique protein |
| 107674 | 12,588 | 12,277 | 0,00327 | 389,941 | unique protein |
| 107641 | 106,103 | 77,556 | 0,00386 | 297,752 | unique protein |
| 111865 | 10,36 | 5,13 | 0,0123 | 65,907 | unique protein |
| 106444 | 5,438 | 7,76 | 0,0335 | 22,724 | unique protein |
| 104455 | 4,215 | 8,984 | 0,0025 | 630,449 | unique protein |
| 109907 | 6,798 | 17,705 | 0,00459 | 227,719 | unique protein |
| 111764 | 4,511 | 5,208 | 0,00165 | 1296,348 | unique protein |
| 102500 | 299,844 | 90,877 | 0,00246 | 670,657 | unique protein |
| 122941 | 5,091 | 2,811 | 0,00783 | 112,724 | unique protein |
| 109081 | 7,704 | 3,407 | 0,0188 | 41,127 | unique protein |
| 103028 | 4,031 | 2,267 | 0,00517 | 191,504 | unique protein |
| 103002 | 3,405 | 4,538 | 0,0168 | 46,838 | unique protein |
| 108143 | 2,193 | 57,831 | 0,00162 | 1419,774 | unique protein |
| 103907 | 7,1 | 2,946 | 0,0165 | 47,784 | unique protein |
| 124296 | 3,821 | 10,842 | 0,00325 | 392,581 | unique protein |
| 111372 | 2,8 | 8,091 | 0,00925 | 92,707 | unique protein |
| 105156 | 894,882 | 700,26 | 0,00145 | 1952,089 | unique protein |
| 105155 | 200,595 | 219,014 | 0,00137 | 2179,571 | unknon protein |
| 74214 | 4,637 | 6,69 | 0,00328 | 385,702 | unknown protein |
| 105860 | 10,902 | 2,447 | 0,00148 | 1791,129 | unknown protein |
| 2365 | 2,133 | 2,062 | 0,0329 | 23,195 | unknown protein |
| 81473 | 3,024 | 2,323 | 0,019 | 40,5 | unknown protein |
| 37665 | 8,425 | 2,238 | 0,00384 | 299,233 | unknown protein |
| 105454 | 16,309 | 3,026 | 0,00379 | 305,948 | unknown protein |
| 74486 | 3,137 | 2,762 | 0,0134 | 59,632 | unknown protein |
| 109793 | 2,71 | 3,474 | 0,0359 | 21,145 | unknown protein |
| 121325 | 11,738 | 10,222 | 0,00216 | 877,551 | unknown protein |
| 59900 | 9,867 | 10,503 | 0,00501 | 200,133 | unknown protein |
| 32798 | 9,635 | 9,653 | 0,00216 | 883,512 | unknown protein |
| 106627 | 9,445 | 3,942 | 0,00473 | 216,963 | unknown protein |
| 59362 | 9,322 | 16,798 | 0,00467 | 221,939 | unknown protein |
| 108018 | 8,838 | 32,025 | 0,00378 | 307,205 | unknown protein |
| 104067 | 8,617 | 9,864 | 0,00261 | 595,501 | unknown protein |
| 59625 | 8,195 | 5,155 | 0,00486 | 210,807 | unknown protein |
| 105455 | 8,14 | 4,14 | 0,00328 | 383,81 | unknown protein |
| 69528 | 8,119 | 6,981 | 0,00493 | 204,112 | unknown protein |
| 104359 | 7,355 | 5,787 | 0,0225 | 34,021 | unknown protein |
| 120415 | 7,209 | 2,55 | 0,00109 | 3833,012 | unknown protein |
| 61134 | 7,049 | 3,991 | 0,00746 | 120,124 | unknown protein |
| 54622 | 64,883 | 22,825 | 0,0038 | 304,029 | unknown protein |
| 112288 | 6,959 | 3,939 | 0,00262 | 573,412 | unknown protein |
| 106147 | 6,93 | 3,601 | 0,00635 | 147,535 | unknown protein |
| 48280 | 6,923 | 7,827 | 0,0329 | 23,145 | unknown protein |
| 73904 | 6,907 | 8,966 | 0,00401 | 277,316 | unknown protein |
| 105290 | 6,872 | 4,345 | 0,0104 | 81,387 | unknown protein |
| 63107 | 6,722 | 3,583 | 0,00518 | 190,683 | unknown protein |
| 108583 | 6,706 | 21,234 | 0,00364 | 323,14 | unknown protein |
| 105465 | 6,139 | 9,54 | 0,00575 | 167,422 | unknown protein |
| 110740 | 5,713 | 49,104 | 0,00491 | 207,764 | unknown protein |
| 103973 | 5,654 | 7,015 | 0,0238 | 32,007 | unknown protein |
| 43195 | 5,631 | 4,192 | 0,00404 | 274,071 | unknown protein |
| 42571 | 5,416 | 5,917 | 0,00373 | 313,105 | unknown protein |
| 111307 | 5,392 | 5,565 | 0,0114 | 71,958 | unknown protein |
| 61715 | 5,315 | 5,391 | 0,0168 | 46,809 | unknown protein |
| 120980 | 5,18 | 2,114 | 0,00959 | 88,803 | unknown protein |
| 26277 | 5,058 | 4,233 | 0,0129 | 62,356 | unknown protein |
| 110173 | 46,862 | 4,723 | 0,00164 | 1529,435 | unknown protein |
| 106556 | 46,782 | 2,297 | 0,00244 | 679,385 | unknown protein |
| 61995 | 4,893 | 5,249 | 0,00989 | 85,68 | unknown protein |
| 106982 | 4,855 | 4,44 | 0,00358 | 331,674 | unknown protein |
| 22386 | 4,819 | 4,882 | 0,00929 | 92,229 | unknown protein |
| 63899 | 4,779 | 6,872 | 0,00244 | 678,301 | unknown protein |
| 104219 | 4,758 | 17,311 | 0,0025 | 634,967 | unknown protein |
| 58519 | 4,624 | 3,365 | 0,00643 | 145,5 | unknown protein |
| 60616 | 4,616 | 3,72 | 0,0103 | 82,127 | unknown protein |
| 47424 | 4,595 | 13,138 | 0,00701 | 128,817 | unknown protein |
| 120579 | 4,487 | 2,261 | 0,0105 | 79,927 | Unknown protein |
| 104494 | 4,451 | 4,078 | 0,0256 | 29,672 | unknown protein |
| 69171 | 4,439 | 2,749 | 0,00838 | 104,148 | unknown protein |
| 64778 | 4,392 | 2,627 | 0,0181 | 43,132 | unknown protein |
| 104898 | 4,372 | 3,004 | 0,00685 | 132,644 | unknown protein |
| 74570 | 4,276 | 2,124 | 0,00454 | 231,583 | unknown protein |
| 107639 | 4,274 | 4,428 | 0,00394 | 286,964 | unknown protein |
| 78576 | 4,269 | 3,915 | 0,0179 | 43,48 | unknown protein |
| 80115 | 4,264 | 2,902 | 0,00819 | 106,505 | unknown protein |
| 81329 | 4,26 | 3,536 | 0,00479 | 215,267 | unknown protein |
| 71123 | 4,215 | 11,413 | 0,00324 | 398,352 | unknown protein |
| 102454 | 4,158 | 3,619 | 0,00822 | 106,179 | unknown protein |
| 63558 | 4,081 | 9,162 | 0,007 | 129,094 | unknown protein |
| 106081 | 4,081 | 2,428 | 0,0122 | 66,516 | unknown protein |
| 59763 | 4,058 | 3,649 | 0,016 | 49,202 | unknown protein |
| 56344 | 4,036 | 2,147 | 0,00293 | 467,551 | unknown protein |
| 2852 | 32,092 | 15,929 | 0,00465 | 224,534 | unknown protein |
| 81586 | 30,738 | 19,412 | 0,00391 | 291,226 | unknown protein |
| 109372 | 3,937 | 6,271 | 0,00538 | 181,445 | unknown protein |
| 66562 | 3,916 | 3,883 | 0,012 | 68,297 | unknown protein |
| 21452 | 3,772 | 2,108 | 0,00775 | 114,72 | unknown protein |
| 54819 | 3,753 | 2,699 | 0,0404 | 18,807 | unknown protein |
| 105133 | 3,607 | 6,621 | 0,00443 | 239,083 | unknown protein |
| 70204 | 3,548 | 4,098 | 0,014 | 56,535 | unknown protein |
| 68618 | 3,525 | 2,161 | 0,0226 | 33,8 | unknown protein |
| 70195 | 3,514 | 2,198 | 0,0183 | 42,291 | unknown protein |
| 59558 | 3,462 | 3,196 | 0,0274 | 27,728 | unknown protein |
| 53314 | 3,419 | 3,318 | 0,0155 | 50,794 | unknown protein |
| 48883 | 3,384 | 2,298 | 0,0184 | 42,148 | unknown protein |
| 110456 | 3,379 | 9,429 | 0,00725 | 123,72 | unknown protein |
| 120332 | 3,364 | 2,531 | 0,00785 | 112,338 | unknown protein |
| 105628 | 3,297 | 3,226 | 0,046 | 16,48 | unknown protein |
| 56682 | 3,286 | 11,05 | 0,0042 | 259,22 | unknown protein |
| 76502 | 3,267 | 2,327 | 0,0237 | 32,222 | unknown protein |
| 110922 | 3,23 | 3,79 | 0,0147 | 53,502 | unknown protein |
| 69303 | 3,228 | 14,356 | 0,00162 | 1327,519 | unknown protein |
| 81756 | 3,214 | 4,37 | 0,00394 | 287,307 | unknown protein |
| 103112 | 3,212 | 33,524 | 0,00355 | 339,554 | unknown protein |
| 109815 | 3,19 | 3,006 | 0,0179 | 43,435 | unknown protein |
| 111332 | 3,142 | 3,574 | 0,0197 | 39,131 | unknown protein |
| 106606 | 3,139 | 13,767 | 0,00395 | 283,104 | unknown protein |
| 78044 | 3,068 | 2,391 | 0,025 | 30,449 | unknown protein |
| 1927 | 3,067 | 3,644 | 0,00112 | 4120,916 | unknown protein |
| 58640 | 3,065 | 2,685 | 0,0121 | 67,477 | unknown protein |
| 50429 | 3,047 | 2,569 | 0,0218 | 35,08 | unknown protein |
| 56196 | 3,025 | 2,041 | 0,00806 | 109,017 | unknown protein |
| 106797 | 3,015 | 5,681 | 0,0148 | 53,255 | unknown protein |
| 23240 | 28,252 | 3,049 | 0,00201 | 1011,392 | unknown protein |
| 70996 | 27,61 | 6,062 | 0,0112 | 73,186 | unknown protein |
| 110267 | 27,219 | 2,888 | 0,00262 | 596,237 | unknown protein |
| 111251 | 23,083 | 68,275 | 0,00159 | 1590,898 | unknown protein |
| 105154 | 22,814 | 25,455 | 0,0135 | 59,222 | unknown protein |
| 55374 | 22,716 | 32,858 | 0,00241 | 692,076 | unknown protein |
| 105157 | 22,49 | 15,797 | 0,00376 | 309,392 | unknown protein |
| 103073 | 2,999 | 2,865 | 0,0358 | 21,168 | unknown protein |
| 33067 | 2,994 | 2,894 | 0,00311 | 419,765 | unknown protein |
| 61223 | 2,97 | 3,192 | 0,00534 | 183,569 | unknown protein |
| 122043 | 2,94 | 3,415 | 0,00539 | 180,973 | unknown protein |
| 81819 | 2,929 | 6,114 | 0,00275 | 525,446 | unknown protein |
| 39827 | 2,91 | 3,533 | 0,0356 | 21,268 | unknown protein |
| 120359 | 2,895 | 6,171 | 0,00393 | 284,905 | unknown protein |
| 111027 | 2,883 | 5,122 | 0,0214 | 35,801 | unknown protein |
| 43101 | 2,869 | 10,918 | 0,00211 | 938,745 | unknown protein |
| 58602 | 2,864 | 5,391 | 0,0085 | 102,339 | unknown protein |
| 68876 | 2,844 | 2,254 | 0,0196 | 39,181 | unknown protein |
| 77915 | 2,821 | 3,183 | 0,0141 | 56,163 | unknown protein |
| 41501 | 2,794 | 5,864 | 0,00114 | 4167,285 | unknown protein |
| 67008 | 2,74 | 2,627 | 0,0197 | 39,052 | unknown protein |
| 108542 | 2,738 | 2,765 | 0,0042 | 259,61 | unknown protein |
| 103822 | 2,73 | 4,864 | 0,00397 | 279,782 | unknown protein |
| 60698 | 2,688 | 2,16 | 0,0172 | 45,294 | unknown protein |
| 82032 | 2,68 | 8,093 | 0,00338 | 368,435 | unknown protein |
| 120195 | 2,677 | 2,984 | 0,0182 | 42,781 | unknown protein |
| 62130 | 2,67 | 3,412 | 0,0149 | 52,839 | unknown protein |
| 66609 | 2,66 | 2,259 | 0,00291 | 478,702 | unknown protein |
| 109373 | 2,652 | 5,399 | 0,0188 | 41,231 | unknown protein |
| 106116 | 2,64 | 5,569 | 0,05 | 15,123 | unknown protein |
| 109071 | 2,638 | 5,231 | 0,0218 | 35,174 | unknown protein |
| 121315 | 2,629 | 2,214 | 0,0349 | 21,751 | unknown protein |
| 102976 | 2,619 | 2,27 | 0,0175 | 44,569 | unknown protein |
| 123476 | 2,604 | 9,689 | 0,00356 | 338,728 | unknown protein |
| 120600 | 2,602 | 4,117 | 0,00913 | 94,049 | unknown protein |
| 76098 | 2,6 | 2,563 | 0,00301 | 441,697 | unknown protein |
| 73760 | 2,592 | 3,786 | 0,0144 | 54,81 | unknown protein |
| 65437 | 2,56 | 2,363 | 0,026 | 29,133 | unknown protein |
| 77629 | 2,546 | 2,277 | 0,0436 | 17,391 | unknown protein |
| 65522 | 2,537 | 2,902 | 0,0102 | 83,149 | unknown protein |
| 64358 | 2,528 | 2,097 | 0,00779 | 113,601 | unknown protein |
| 105291 | 2,506 | 5,156 | 0,0346 | 21,942 | unknown protein |
| 55172 | 2,488 | 2,246 | 0,0181 | 43,129 | unknown protein |
| 56671 | 2,487 | 2,273 | 0,0185 | 41,863 | unknown protein |
| 57865 | 2,486 | 2,329 | 0,0181 | 42,938 | unknown protein |
| 3063 | 2,481 | 5,241 | 0,00413 | 265,845 | unknown protein |
| 121175 | 2,466 | 2,753 | 0,0373 | 20,36 | unknown protein |
| 65933 | 2,45 | 2,861 | 0,0419 | 18,104 | unknown protein |
| 111450 | 2,448 | 3,333 | 0,011 | 75,93 | unknown protein |
| 81259 | 2,445 | 2,382 | 0,0167 | 47,158 | unknown protein |
| 103031 | 2,441 | 3,746 | 0,00775 | 114,522 | unknown protein |
| 105882 | 2,429 | 2,68 | 0,00656 | 140,699 | unknown protein |
| 102668 | 2,406 | 2,275 | 0,0246 | 30,999 | unknown protein |
| 58910 | 2,405 | 2,132 | 0,0112 | 73,219 | unknown protein |
| 122792 | 2,396 | 12,342 | 0,00438 | 244,186 | unknown protein |
| 108953 | 2,393 | 4,759 | 0,0157 | 49,931 | unknown protein |
| 102461 | 2,383 | 2,392 | 0,0183 | 42,395 | unknown protein |
| 122455 | 2,382 | 3,244 | 0,0186 | 41,754 | unknown protein |
| 108144 | 2,35 | 53,235 | 0,00225 | 802,93 | unknown protein |
| 106219 | 2,28 | 2,605 | 0,0226 | 33,831 | unknown protein |
| 108866 | 2,279 | 2,275 | 0,0344 | 22,102 | unknown protein |
| 110761 | 2,278 | 5,404 | 0,0067 | 136,728 | unknown protein |
| 44684 | 2,277 | 2,393 | 0,0145 | 54,413 | unknown protein |
| 56771 | 2,237 | 5,699 | 0,00151 | 1808,353 | unknown protein |
| 74534 | 2,23 | 2,402 | 0,0256 | 29,648 | unknown protein |
| 62086 | 2,222 | 2,997 | 0,00404 | 273,708 | unknown protein |
| 65949 | 2,214 | 11,214 | 0,0038 | 303,391 | unknown protein |
| 119642 | 2,214 | 3,112 | 0,00514 | 192,897 | unknown protein |
| 104291 | 2,199 | 2,157 | 0,023 | 33,289 | unknown protein |
| 35183 | 2,17 | 2,288 | 0,0218 | 35,126 | unknown protein |
| 111103 | 2,166 | 2,648 | 0,0364 | 20,82 | unknown protein |
| 5072 | 2,165 | 2,1 | 0,0186 | 41,614 | unknown protein |
| 122679 | 2,149 | 3,412 | 0,00109 | 3261,033 | unknown protein |
| 21407 | 2,128 | 2,364 | 0,0111 | 74,934 | unknown protein |
| 56835 | 2,102 | 2,336 | 0,00581 | 165,288 | unknown protein |
| 108089 | 2,101 | 2,042 | 0,0191 | 40,502 | unknown protein |
| 77222 | 2,1 | 2,147 | 0,0191 | 40,531 | unknown protein |
| 45252 | 2,088 | 2,924 | 0,0158 | 49,742 | unknown protein |
| 74932 | 2,088 | 5,29 | 0,0126 | 64,215 | unknown protein |
| 53492 | 2,083 | 2,357 | 0,00653 | 141,982 | unknown protein |
| 122825 | 2,056 | 4,869 | 0,00891 | 96,582 | unknown protein |
| 63869 | 2,039 | 3,189 | 0,00639 | 146,544 | unknown protein |
| 108566 | 2,034 | 3,373 | 0,0136 | 58,734 | unknown protein |
| 119881 | 2,03 | 2,772 | 0,0201 | 38,233 | unknown protein |
| 106353 | 2,018 | 3,747 | 0,00397 | 280,24 | unknown protein |
| 64972 | 2,009 | 2,95 | 0,00589 | 161,282 | unknown protein |
| 68588 | 15,645 | 23,202 | 0,00115 | 4245,56 | unknown protein |
| 109934 | 14,727 | 3,437 | 0,00493 | 204,034 | unknown protein |
| 107244 | 14,42 | 12,726 | 0,00734 | 122,138 | unknown protein |
| 43401 | 13,529 | 16,342 | 0,0126 | 64,073 | unknown protein |
| 110457 | 13,175 | 35,762 | 0,00178 | 1191,212 | unknown protein |
| 29275 | 12,714 | 16,551 | 0,00887 | 97,001 | unknown protein |
| 74449 | 12,629 | 7,511 | 0,0111 | 74,963 | unknown protein |
| 105820 | 11,475 | 7,246 | 0,00314 | 413,45 | unknown protein |
| 121111 | 10,689 | 5,43 | 0,00986 | 85,927 | unknown protein |
| 54426 | 10,307 | 2,124 | 0,00971 | 87,371 | unknown protein |
| 111584 | 10,139 | 2,107 | 0,00451 | 233,654 | unknown protein |
| 34183 | 2,358 | 2,004 | 0,0157 | 50,061 | unknown protein |
| 112163 | 19,202 | 4,011 | 0,00301 | 446,125 | unknown protein |
| 123086 | 493,223 | 465,12 | 0,00139 | 2132,948 | unknown protein |
| 107667 | 7,063 | 2,472 | 0,00704 | 127,889 | unknown protein |
| 80578 | 4,868 | 6,987 | 0,00658 | 140,406 | unknown protein |
| 110893 | 4,292 | 5,741 | 0,00503 | 198,127 | unknown protein |
| 58396 | 3,243 | 2,656 | 0,00819 | 106,482 | unknown protein |
| 39426 | 26,795 | 3,057 | 0,00345 | 353,596 | unknown protein |
| 120558 | 2,087 | 4,096 | 0,00494 | 203,14 | unknown protein |
| 53525 | 7,272 | 3,552 | 0,00346 | 349,583 | unknown protein |
| 52165 | 3,099 | 11,24 | 0,00543 | 179,313 | unknown protein |
| 108683 | 2,453 | 2,439 | 0,0156 | 50,459 | unknown protein |
| 110035 | 8,621 | 2,235 | 0,00816 | 107,233 | unknown protein |
| 109239 | 331,67 | 79,371 | 0,000875 | 6694,241 | unknown protein |
| 106049 | 2,116 | 3,331 | 0,0406 | 18,654 | unknown protein |
| 103143 | 2,216 | 2,077 | 0,0423 | 17,913 | unknown protein |
| 59065 | 3,373 | 3,12 | 0,00877 | 98,694 | unknown protein |
| 56146 | 2,406 | 3,695 | 0,0219 | 34,964 | unknown protein with DUF500 domain |
| 58851 | 4,696 | 2,9 | 0,00109 | 3191,974 | unknown protein with F-box and JmjC domain |
| 75975 | 2,058 | 3,209 | 0,0396 | 19,122 | unknown protein with LMBR1 domain |
| 41152 | 18,143 | 3,744 | 0,00245 | 668,902 | unknown protein with NACHT domain |
| 107949 | 7,368 | 2,455 | 0,00465 | 224,016 | unknown protein with ThiJ/PfpI domain |
| 112387 | 10,877 | 8,033 | 0,0141 | 56,166 | unknown protein with WD40 repeats |
| 73523 | 2,222 | 2,127 | 0,0205 | 37,52 | unknown protein with WSC domains |
| 3363 | 15,468 | 38,476 | 0,0011 | 3842,735 | unknown protein, 9 TM |
| 109435 | 2,114 | 2,833 | 0,00853 | 101,91 | unknown protein, 1 TM |
| 76360 | 38,618 | 7,534 | 0,00245 | 666,793 | unknown protein, 1 TM domain |
| 41149 | 2,36 | 2,602 | 0,00582 | 164,045 | unknown protein, 2 TM |
| 105840 | 10,339 | 9,489 | 0,00264 | 558,977 | unknown protein, 2 TMs |
| 2071 | 2,284 | 2,114 | 0,0126 | 64,582 | unknown protein, 5 TM |
| 109387 | 12,834 | 6,255 | 0,00215 | 907,146 | unknown protein, in Aspergilli |
| 79345 | 2,31 | 2,243 | 0,0176 | 44,196 | unknown protein, Mpv17/PMP22 family |
| 3987 | 2,485 | 2,284 | 0,0255 | 29,772 | unknown protein, only in fungi |
| 122724 | 4,026 | 5,297 | 0,00483 | 212,053 | unknown protein, only in neurospora and Chaetomium |
| 76359 | 6,103 | 29,107 | 0,00383 | 300,364 | unknown protein, only present in ascomycota |
| 111739 | 12,607 | 9,737 | 0,00334 | 374,967 | unknown protein, only present in Aspergilli |
| 34272 | 19,224 | 2,103 | 0,0067 | 136,498 | unknown protein, only present in Neurospora, Magnaporthe, Chaetomium |
| 109905 | 12,12 | 3,946 | 0,0105 | 80,337 | unknown protein, potentially a transferase of secondary metabolism ? |
| 120927 | 7,652 | 11,035 | 0,0168 | 46,852 | unknown protein, related to A. oryzae ankyrin |
| 3267 | 2,671 | 4,591 | 0,0122 | 66,997 | unknown protein, secreted |
| 111345 | 3,041 | 2,817 | 0,0482 | 15,686 | unknown protein, Sordariomycetes, 1 TM |
| 106626 | 11,115 | 3,964 | 0,00357 | 332,941 | unknown protein, SWIFT domain |
| 4428 | 3,26 | 2,031 | 0,0112 | 73,004 | unknown protein, TPR domain |
| 55666 | 2,259 | 5,284 | 0,0232 | 32,998 | unknown protein, WD repeats |
| 30465 | 413,841 | 903,975 | 0,000761 | 15889,13 | unknown protein, WD-repeats |
| 102735 | 2,682 | 2,438 | 0,037 | 20,506 | unknown protein, WSC domain |
| 107881 | 4,904 | 189,106 | 0,00156 | 1627,356 | unknown protein; distant similarity in yeast is a stop codon bypass factor |
| 121818 | 6,285 | 3,559 | 0,00827 | 105,416 | unknown secreted protein |
| 105444 | 384,377 | 49,694 | 0,00397 | 279,624 | unknown secreted protein |
| 120953 | 32,846 | 8,16 | 0,00329 | 381,23 | unknown secreted protein |
| 105977 | 5,927 | 7,327 | 0,00776 | 114,387 | unknown secreted protein, 3 TM |
| 68997 | 6,352 | 19,158 | 0,00322 | 402,372 | unknown TPR domain protein, unknown |
| 104353 | 38,05 | 11,177 | 0,0016 | 1441,941 | unknown unknown protein, contains F-box |
| 58603 | 5,996 | 2,797 | 0,0134 | 59,867 | unknown unknown protein, contains Splecktrin motif |
| 36373 | 4,269 | 8,15 | 0,0022 | 832,738 | Vacuolar assembly/sorting protein VPS9 |
| 61470 | 2,406 | 2,342 | 0,00263 | 598,964 | Vesicle coat complex COPII, Sar1 GTPase |
| 121350 | 20,404 | 41,359 | 0,00149 | 1739,305 | xenobiotic monooxygenase CipC1 |
| 30759 | 17,756 | 5,876 | 0,00264 | 565,968 | zinc containing alcohol dehydrogenase superfamily |
| 52463 | 3,604 | 5,897 | 0,00245 | 675,596 | zinc metalloprotease |
| 123325 | 5,294 | 5,539 | 0,00656 | 141 | zinc transporter |
| 107479 | 3,839 | 7,417 | 0,0074 | 120,905 | zinc/iron transporter |
| 79738 | 12,271 | 6,376 | 0,00457 | 229,083 | Zinc-binding oxidoreductase |
| 77770 | 3,042 | 2,325 | 0,0261 | 28,954 | Zinc-containing alcohol dehydrogenase |
| 102499 | 594,508 | 104,325 | 0,00216 | 876,054 | Zn2Cys6 transcriptional regulator |
| 39221 | 5,63 | 7,868 | 0,00278 | 512,19 | Zn2Cys6 transcriptional regulator |
| 26871 | 3,403 | 2,304 | 0,0191 | 40,312 | Zn2Cys6 transcriptional regulator |
| 66047 | 3,333 | 2,225 | 0,00546 | 177,544 | Zn2Cys6 transcriptional regulator |
| 102497 | 10,737 | 8,377 | 0,00359 | 328,959 | Zn2Cys6 transcriptional regulator |
| 75204 | 4,116 | 2,056 | 0,00519 | 189,791 | Zn-finger protein AN1 |
| 103669 | 6,19 | 2,973 | 0,00766 | 116,604 | α/ß hydrolase lipase |

*GL, fold increase/decrease on lactose vs glucose; **YL, fold increase/decrease on lactose vs glycerol
