# Supplementary material for: Systems Analysis of Lactose Metabolism in Trichoderma reesei Identifies a Lactose Permease That Is Essential for Cellulase Induction
Source: PLoS One. 2013 May 8;8(5):e62631. doi: 10.1371/journal.pone.0062631 (PMC3648571; doi:10.1371/journal.pone.0062631)
Supplement: Table S2 — Genes significantly regulated on lactose and by CRE1 and/or growth rate. (DOCX) [file pone.0062631.s003.docx]

**Table S2.** Genes significantly regulated on lactose and by CRE1 and/or growth rate*

| **Protein ID** | **GL** | **YL** | **Annotation** | ***cre1* regulation** |
| --- | --- | --- | --- | --- |
| 22912 | 51,688 | 8,802 | glucose transporter HXT1 | A |
| 123234 | 4,847 | 5,455 | Coproporphyrinogen III oxidase | A |
| 104322 | 38,115 | 6,253 | unique protein | A |
| 74807 | 3,086 | 3,263 | hypothetical glycosyl hydrolase, not aligned with a GH family | A |
| 79677 | 2,241 | 3,451 | N-acetyl-glucosamine kinase | A |
| 45717 | 11,069 | 8,48 | GH47 α-1,2-mannosidase | A |
| 22459 | 10,917 | 2,56 | putative carboxypeptidase A | A |
| 49205 | 3,481 down | 2,488 down | cytochrome C peroxidase | B |
| 82032 | 2,680 down | 8,093 down | hypothetical protein | B |
| 65522 | 2,537 down | 2,902 down | unique protein | B |
| 76722 | 2,273 down | 3,891 down | flavohemoglobin | B |
| 77481 | 2,040 down | 2,783 down | D-xylulose 5-phosphate/D-fructose 6-phosphate phosphoketolase | B |
| 111082 | 11,895 down | 8,214 down | putative glutathione S transferase | B |
| 81022 | 4,222 | 3,303 | allantoate permease | C |
| 70860 | 3,934 | 3,631 | allantoate permease | C |
| 62172 | 3,319 | 2,631 | amino acid permease | C |
| 123718 | 628,761 down | 496,845 down | neutral amino acid permease | C |
| 82095 | 5,157 down | 3,130 down | high affinity ammonium transporter | C |
| 121136 | 31,180 down | 247,126 down | unique protein | C |
| 80086 | 15,511 down | 2,062 down | MSF peptide transporter | C |
| 3363 | 15,468 down | 38,476 down | hypothetical protein with 9 transmembrane domains | C |
| 81778 | 8,115 | 4,276 | glutaminase A | D |
| 80659 | 70,153 | 203,478 | alcohol oxidase AOX1 | D |
| 60489 | 52,831 | 21,566 | CE5 cutinase | D |
| 74198 | 40,21 | 20,923 | GH92 α-1,2-mannosidase | D |
| 121475 | 4,667 | 2,379 | unique protein | D |
| 120784 | 4,27 | 2,734 | putative cell wall mannoprotein | D |
| 55319 | 3,309 | 5,048 | GH54, L-α-arabinofuranosidase | D |
| 65741 | 3,001 | 2,394 | hypothetical transmembrane protein | D |
| 123978 | 29,714 | 12,931 | hypothetical methanol oxidase | D |
| 77093 | 23,571 | 30,769 | acid sphingomyelin phosphodiesterase (probably vacuolar) | D |
| 38812 | 154,968 | 301,65 | iron transporter | D |
| 69245 | 101,158 | 38,535 | GH2 ß-mannosidase | D |
| 108143 | 2,193 down | 57,831 down | unique protein | D |
| 45445 | 6,39 | 2,04 | tyrosinase | E |
| 80149 | 5,419 | 2,942 | hypothetical protein, conserved | E |
| 23415 | 4,576 | 15,128 | amino acid permease Dip5 | E |
| 46794 | 4,221 | 12,688 | MSF peptide transporter | E |
| 58282 | 3,346 | 2,096 | CE9 esterase | E |
| 80863 | 2,624 | 2,358 | GH18 endochitinase CHI18-5 | E |
| 120017 | 2,414 | 4,723 | small oligopeptide transporter | E |
| 74282 | 2,12 | 8,081 | hypothetical cysteine-rich protein | E |
| 76897 | 171,453 | 26,09 | MSF transporter | E |
| 123456 | 11,275 | 2,234 | GH65 αα'-trehalase | E |
| 123468 | 9,888 down | 5,014 down | IMP dehydrogenase | F |
| 80879 | 81,861 down | 45,529 down | H+/oligopeptide transporter | F |
| 102499 | 594,508 down | 104,325 down | Zn2Cys6-transcription factor | F |
| 123084 | 54,473 down | 45,916 down | (chloro)peroxidase | F |
| 106556 | 46,782 down | 2,297 down | hypothetical protein | F |
| 60616 | 4,616 down | 3,720 down | integral membrane protein | F |
| 107639 | 4,274 down | 4,428 down | hypothetical protein | F |
| 59014 | 3,961 down | 2,401 down | ABC multidrug transporter | F |
| 73621 | 255,424 down | 117,144 down | polyketide synthase | F |
| 23382 | 24,352 down | 6,148 down | aldehyde reductase AKR7 | F |
| 73623 | 1986,095 down | 1234,879 down | FAD-monooxygenase | F |
| 81525 | 19,159 down | 6,699 down | isoflavone reductase | F |
| 30759 | 17,756 down | 5,876 down | protein of zinc containing alcohol dehydrogenase superfamily | F |
| 73631 | 166,217 down | 100,128 down | isoamyl alcohol oxidase | F |
| 82204 | 14,408 down | 4,517 down | high affinity ammonium transporter | F |
| 73618 | 139,697 down | 54,849 down | polyketide synthase | F |
| 76763 | 13,486 down | 7,007 down | PTH11-type G-coupled receptor | F |
| 3327 | 11,032 down | 5,963 down | NADH:flavin oxidoreductase/NADH oxidase | F |
| 43701 | 101,205 down | 97,534 down | MSF multidrug transporter | F |
| 102497 | 10,737 down | 8,377 down | Zn2Cys6 transcription factor | F |
| 105106 | 7,594 | 2,127 | hypothetical protein | G |
| 59151 | 29,897 | 2,031 | BYS1 domain protein (=Blastomyces yeast-phase-specific protein) secreted | G |
| 71532 | 2,649 | 7,183 | GH71 α-1,3-glucanase | G |
| 105313 | 8,665 down | 41,937 down | unique protein | G |
| 121818 | 6,285 down | 3,559 down | hypothetical secreted protein | G |
| 107881 | 4,904 down | 189,106 down | hypothetical protein | G |
| 111750 | 23,153 down | 23,115 down | ferric reductase | G |
| 77547 | 2,640 down | 2,285 down | GT1 glycosyltransferase | G |
| 65406 | 2,090 down | 3,055 down | GH16, glucanosyltransferase MWG2 (orthologue of A. fumgatus Crf1) | G |
| 52315 | 1756,734 down | 1966,144 down | Ctr copper transporter | G |
| 112258 | 8,938 | 8,244 | hypothetical protein | H |
| 107960 | 7,494 | 4,339 | hypothetical protein with WSC-carbohydrate binding domain, secreted | H |
| 108642 | 64,054 | 75,806 | hypothetical secreted protein | H |
| 55886 | 4,553 | 3,907 | GH16 ß-glycosidase (GPI anchor) | H |
| 54352 | 33,965 | 2,775 | hypothetical protain, conserved | H |
| 47066 | 8,259 down | 2,943 down | candidate sulfate adenylyltransferase | H |
| 70520 | 52,084 down | 41,005 down | short chain dehydrogenase/reductase | H |
| 76633 | 5,107 down | 5,726 down | MDR transporter | H |
| 81576 | 45,898 down | 12,741 down | hypothetical assimilatory sulfite reductase, alpha subunit | H |
| 79741 | 26,586 down | 5,900 down | ER-bound Farnesyl-diphosphate farnesyltransferase | H |
| 65410 | 12,926 down | 3,953 down | Phosphoadenosine phosphosulfate reductase | H |
| 60847 | 4,140 down | 2,608 down | ATP synthase subunit 9, proteolipid P2 | X |
| 43671 | 22,897 down | 16,919 down | ammonium permease MEA1 | X |
| 81690 | 2,961 down | 2,453 down | orthologue of Aspergillus nidulans CreD | X |
| 1751 | 2,886 down | 3,963 down | FAD monooxygenase | X |
| 121534 | 2,450 down | 2,955 down | pyruvate decarboxylase | X |
| 123382 | 2,268 down | 3,427 down | subtilisin-type serine protease | X |
| 39637 | 2,187 down | 5,115 down | C4-dicarboxylate transporter | X |
| 49274 | 19,080 down | 11,250 down | GH16 ß-1,3/4-glucanase | X |

* abbreviations are used as in Supplementary Table S1. The letters in the column “*cre1* regulation” refer to the regulatory pattern as described by Portnoy et al. [9].
