# Supplementary material for: Systems Analysis of Lactose Metabolism in Trichoderma reesei Identifies a Lactose Permease That Is Essential for Cellulase Induction
Source: PLoS One. 2013 May 8;8(5):e62631. doi: 10.1371/journal.pone.0062631 (PMC3648571; doi:10.1371/journal.pone.0062631)
Supplement: Table S3 — Expression rates of the ten most upregulated MFS permeases determined qPCR analysis. (DOCX) [file pone.0062631.s004.docx]

**Table S3.** Expression ratios* of the ten most upregulated MFS permeases determined qPCR analysis. The results and standard errors of three independent experiments are shown.

| **Gene ID** | **Ratio**** |  |
| --- | --- | --- |
| 105260 | 0.14 ± 0.17 |  |
| 3405 | 758.32 ± 346.77 |  |
| 79202 | 165.80 ± 42.40 |  |
| 56289 | 2.16 ± 0.23 |  |
| 104072 | 28.05 ± 16.04 |  |
| 50894 | 2.11 ± 0.56 |  |
| 56684 | 7.67 ± 5.74 |  |
| 70349 | 1.59 ± 0.15 |  |
| 69957 | 1.25 ± 0.68 |  |
| 121608 | 0.76 ± 0.16 |  |

*Ratios are defined as the normalized expression of the gene on lactose versus expression of the gene on glucose. The consitutively expressed gene *tef1* was used for normalization.

** RNA was isolated after 30 hrs of cultivation.
