# Supplementary material for: Systems Analysis of Lactose Metabolism in Trichoderma reesei Identifies a Lactose Permease That Is Essential for Cellulase Induction
Source: PLoS One. 2013 May 8;8(5):e62631. doi: 10.1371/journal.pone.0062631 (PMC3648571; doi:10.1371/journal.pone.0062631)
Supplement: Table S4 — Oligonucleotides used for the construction of deletion cassettes. (DOCX) [file pone.0062631.s005.docx]

**Table S4.** Oligonucleotides used for the construction of deletion cassettes.

| Gene ID | Oligonucleotide | Sequence |
| --- | --- | --- |
| 105260 | 5F_ht | 5‘-GTAACGCCAGGGTTTTCCCAGTCACGACGCCTGGATATCCCATGGATGT-3‘ |
|  | 5R_ht | 5‘-CGACGATATCAGCTTCCATATTCCGACTATGTATGACCAAGAATTTGCC-3‘ |
|  | 3F_ht | 5‘-AGAAAAGCACAAAGAAGAGGCTCCAACTATGGAGCATCAGGGTGAGTAT-3‘ |
|  | 3R_ht | 5‘-GCGGATAACAATTTCACACAGGAAACAGCTTGCCGTATCCTTGTCAT-3‘ |
| 3405 | ch_ht | 5‘-GGAAGTGGGCAGATGTTT-3‘ |
|  | 5F_st | 5‘-GTAACGCCAGGGTTTTCCCAGTCACGACGTTCTTCACACCTAGGTTCG -3‘ |
|  | 5R_st | 5‘-CGACGATATCAGCTTCCATATTCCGACTAGATCTCTCCCTACTCAGGCA-3‘ |
|  | 3F_st | 5‘-AGAAAAGCACAAAGAAGAGGCTCCAACTAATATCTGGGCAGTTGTGCG-3‘ |
|  | 3R_st | 5‘-GCGGATAACAATTTCACACAGGAAACAGCTAGACGGACAGGGAACAGAG-3‘ |
| 79202 | ch_st | 5‘-ATCCGATCATACCTGCCA-3‘ |
|  | 5F_tp | 5‘-GTAACGCCAGGGTTTTCCCAGTCACGACGGGCTTGCCAATCATAACTAA-3‘ |
|  | 5R_tp | 5‘-CGACGATATCAGCTTCCATATTCCGACTACCGTCCTTTGACCGAGAT-3‘ |
|  | 3F_tp | 5‘-AGAAAAGCACAAAGAAGAGGCTCCAACTAAGTTGGGAGTGCATATTGAA-3‘ |
|  | 3R_tp | 5‘-GCGGATAACAATTTCACACAGGAAACAGCATGAGGCTGGACGAATCTAG-3‘ |
| 56289 | ch_tp | 5‘-GGAGATTCCTACGGCTGA-3‘ |
|  | 5F_pmfs1 | 5‘-GTAACGCCAGGGTTTTCCCAGTCACGACGACTGTTGATCGTTCTCCTCC-3‘ |
|  | 5R_pmfs1 | 5‘-CGACGATATCAGCTTCCATATTCCGACTAGGTCAGAGACTGCAAGGCT-3‘ |
|  | 3F_pmfs1 | 5‘-AGAAAAGCACAAAGAAGAGGCTCCAACTAGTATTGCGATCTGTCATTGG-3‘ |
|  | 3R_pmfs1 | 5‘-GCGGATAACAATTTCACACAGGAAACAGCCAGGAGTGGAATACGTATGC-3‘ |
| 123473 | ch_pmfs1 | 5‘-AATCTCTGGAATCACGATGG-3‘ |
|  | 5F_mfs3 | 5‘-GTAACGCCAGGGTTTTCCCAGTCACGACGATAGTCTGGCCATGGGATC-3‘ |
|  | 5R_mfs3 | 5‘-CGACGATATCAGCTTCCATATTCCGACTAAGCAGAACTGAGGGAAACAT-3‘ |
|  | 3F_mfs3 | 5‘-AGAAAAGCACAAAGAAGAGGCTCCAACTACAGCAGCCAATTGTCACG-3‘ |
|  | 3R_mfs3 | 5‘-GCGGATAACAATTTCACACAGGAAACAGCCACTCTCACTCGTCGACACC-3‘ |
| 104072 | ch_pmfs3 | 5‘-GTACGAGTATGGAGCAATGC-3‘ |
|  | 5F_pt | 5‘-GTAACGCCAGGGTTTTCCCAGTCACGACGGGCTTCCCTTGCACTAATG-3‘ |
|  | 5R_pt | 5‘-CGACGATATCAGCTTCCATATTCCGACTAAAATCCGATACATCTTGGC-3‘ |
|  | 3F_pt | 5‘-AGAAAAGCACAAAGAAGAGGCTCCAACTACAGGATCAGCGTGGAATATT-3‘ |
|  | 3R_pt | 5‘-GCGGATAACAATTTCACACAGGAAACAGCAAAGAATCGATCCACTACGG-3‘ |
| 50894 | ch_pt | 5‘-AAATCCAGATACTCCAGTCG-3‘ |
|  | 5F_pmfs4 | 5‘-GTAACGCCAGGGTTTTCCCAGTCACGACGCACTGACGCTAAAGCATACG-3‘ |
|  | 5R_pmfs4 | 5‘-CGACGATATCAGCTTCCATATTCCGACTACCTTGTCTTCATGTCGCCT-3‘ |
|  | 3F_pmfs4 | 5‘-AGAAAAGCACAAAGAAGAGGCTCCAACTACGAGTCACGGATCTTCTGG-3‘ |
|  | 3R_pmfs4 | 5‘-GCGGATAACAATTTCACACAGGAAACAGCATACTTGCCAGCAGCTTCA-3‘ |
| 56684 | ch_pmfs4 | 5‘-GGAGACGGTGTTACATGCT-3‘ |
|  | 5F_pt2 | 5‘-GTAACGCCAGGGTTTTCCCAGTCACGACGCGATTCGACGAGCTACTGAG-3‘ |
|  | 5R_pt2 | 5‘-CGACGATATCAGCTTCCATATTCCGACTAAAATGAAGGGTAAGCAAAGC-3‘ |
|  | 3F_pt2 | 5‘-AGAAAAGCACAAAGAAGAGGCTCCAACTAGTCGTGAAATCGCTAATGAA-3‘ |
|  | 3R_pt2 | 5‘-GCGGATAACAATTTCACACAGGAAACAGCAATTGCCGCTGTGAGTCAT-3‘ |
| 62380 | ch_pt2 | 5‘-TTCGTTTCATCTCAGGCC-3‘ |
|  | 5F_msf2 | 5‘-GTAACGCCAGGGTTTTCCCAGTCACGACGGCTGAATCATACGCATGTTT-3‘ |
|  | 5R_msf2 | 5‘-CGACGATATCAGCTTCCATATTCCGACTATGTGTGAGACCGTGGAGG-3‘ |
|  | 3F_msf2 | 5‘-AGAAAAGCACAAAGAAGAGGCTCCAACTATGGCTGTGGTGAATATCCTA-3‘ |
|  | 3R_msf2 | 5‘-GCGGATAACAATTTCACACAGGAAACAGCGAGTGCTGCTATAGTGCGAA-3‘ |
|  | ch_msf2 | 5‘-CAGTCAGAAGGAGCGGTT-3‘ |
| 44956 | 5F_pmfs2 | 5‘-GTAACGCCAGGGTTTTCCCAGTCACGACGAGCACCAGAGCATCACCAT-3‘ |
|  | 5R_pmfs2 | 5‘-CGACGATATCAGCTTCCATATTCCGACTAGGCGTGCACAGTAGATTCA-3‘ |
|  | 3F_pmfs2 | 5‘-AGAAAAGCACAAAGAAGAGGCTCCAACTACAGGCGACAGACACAATCAT-3‘ |
|  | 3R_pmfs2 | 5‘-GCGGATAACAATTTCACACAGGAAACAGCCTTCGCGTCCTCCATCTC-3‘ |
|  | ch_pmfs2 | 5‘-GGGTTGTCGCAGTTCTCA-3‘ |
| 21595 | 5F_pmfs3 | 5‘-GTAACGCCAGGGTTTTCCCAGTCACGACGGCTGCCAGGGGTGTATATG-3‘ |
|  | 5R_pmfs3 | 5‘-CGACGATATCAGCTTCCATATTCCGACTAGGACCGGGAGAGATGATGT-3‘ |
|  | 3F_pmfs3 | 5‘-AGAAAAGCACAAAGAAGAGGCTCCAACTACGGCAGCTATAACAGACCAC-3‘ |
|  | 3R_pmfs3 | 5‘-GCGGATAACAATTTCACACAGGAAACAGCACATCTTGGCAATCTTGAGG-3‘ |
|  | ch_pmfs3 | 5‘-GTACGAGTATGGAGCAATGC-3‘ |
| 70349 | 5F_pmfs5 | 5‘-GTAACGCCAGGGTTTTCCCAGTCACGACGCTCGTTGCAGTGCTCGTTAT-3‘ |
|  | 5R_pmfs5 | 5‘-CGACGATATCAGCTTCCATATTCCGACTAAATGAGGGCTATGGAACAAA-3‘ |
|  | 3F_pmfs5 | 5‘-AGAAAAGCACAAAGAAGAGGCTCCAACTATCCATATCTTCGAGCTGTGC-3‘ |
|  | 3R_pmfs5 | 5‘-GCGGATAACAATTTCACACAGGAAACAGCGCAGTAGCATCGATAAGCAT-3‘ |
|  | ch_pmfs5 | 5‘-GGGAAAGATAGGTGGCAA-3‘ |
| 69957 | 5F_pt4 | 5‘-GTAACGCCAGGGTTTTCCCAGTCACGACGCAGGTATAATTGTCTCCGCC-3‘ |
|  | 5R_pt4 | 5‘-CGACGATATCAGCTTCCATATTCCGACTACCCTTATACTCGCATGCAGA-3‘ |
|  | 3F_pt4 | 5‘-AGAAAAGCACAAAGAAGAGGCTCCAACTAGCTACTGGAGACAACGACGA-3‘ |
|  | 3R_pt4 | 5‘-GCGGATAACAATTTCACACAGGAAACAGCTGTGGCAGAAGGTCTCGATA-3‘ |
|  | ch_pt4 | 5‘-GCAAAGTAGGCATGATCAAC-3‘ |
| 121608 | 5F_pmfs6 | 5‘-GTAACGCCAGGGTTTTCCCAGTCACGACGCTAGCAGGCATCCGTCTCT-3‘ |
|  | 5R_pmfs6 | 5‘-CGACGATATCAGCTTCCATATTCCGACTAATCTCACGAGCGAAGGAACT-3‘ |
|  | 3F_pmfs6 | 5‘-AGAAAAGCACAAAGAAGAGGCTCCAACTAGTAGCGAGCCAGACACATTC-3‘ |
|  | 3R_pmfs6 | 5‘-GCGGATAACAATTTCACACAGGAAACAGCGACGATGAGGACCACCAGAT-3‘ |
|  | ch_pmfs6 | 5‘-ATCATATGCCTTGTCTCACC-3‘ |
| 65915 | 5F_pmfs7 | 5‘-GTAACGCCAGGGTTTTCCCAGTCACGACGACGATATGGTGCTCTTGACA-3‘ |
|  | 5R_pmfs7 | 5‘-CGACGATATCAGCTTCCATATTCCGACTACAACCGTCTTGAGATCCTTC-3‘ |
|  | 3F_pmfs7 | 5‘-AGAAAAGCACAAAGAAGAGGCTCCAACTACCAGCATAAGCACACTACCC-3‘ |
|  | 3R_pmfs7 | 5‘-GCGGATAACAATTTCACACAGGAAACAGCTCAACAACCTGCAAGAGTCC-3‘ |
|  | ch_pmfs7 | 5‘-GACCTACAGCTTCTAAACGG-3‘ |
| 82309 | 5F_pt5 | 5‘-GTAACGCCAGGGTTTTCCCAGTCACGACGTCAGTTCTATGGCGTATGGG-3‘ |
|  | 5R_pt5 | 5‘-CGACGATATCAGCTTCCATATTCCGACTACTCCCCAGGATTGAGATGC-3‘ |
|  | 3F_pt5 | 5‘-AGAAAAGCACAAAGAAGAGGCTCCAACTAACCAACCTCGATTACCTCAC-3‘ |
|  | 3R_pt5 | 5‘-GCGGATAACAATTTCACACAGGAAACAGCGATGAGATATTCGCCAGCAC-3‘ |
|  | ch_pt5 | 5‘-ATTCACCCGTACGTCGTC-3‘ |
| 78833 | 5F_pfp | 5‘-GTAACGCCAGGGTTTTCCCAGTCACGACGAACAAACTGCAGAGATCCGA-3‘ |
|  | 5R_pfp | 5‘-CGACGATATCAGCTTCCATATTCCGACTATTTGACTGACTTGGGACGAC-3‘ |
|  | 3F_pfp | 5‘-AGAAAAGCACAAAGAAGAGGCTCCAACTAGCCAAACACACATAGACCTG-3‘ |
|  | 3R_pfp | 5‘-GCGGATAACAATTTCACACAGGAAACAGCTAGTCCAGTACCGCAAGAGC-3‘ |
|  | ch_pfp | 5‘-AAGGGAGGAGGTTCACGT-3‘ |
| 74020/pyr4 | Pyr4 fw: | 5‘-ATTCCAACGCCTCTTCTTTGTGCTTTTCT-3‘ |
|  | Pyr4 rev | 5‘-ATTCCGACATATGGAAGCTGATATCGTCG-3‘ |
|  | ch_pyr4 neu | 5‘-ACAAGCCCATCTACTCGAA-3‘ |
| 3405 | st_infusion_1 | 5‘-CGTCATCGATGTCGACAGGGAGAGATCAGGACCAA-3‘ |
|  | st_infusion_2 | 5‘-TGATTACGCCAAGCTTGAGGTAGGCATGGGTGTAT-3‘ |
|  | Ptef F ch | 5‘-CACCATCTGCTTTCGAAA-3‘ |
|  | St R ch | 5‘-CGACGACACCACCAATCT-3‘ |
