# Supplementary material for: Systems Analysis of Lactose Metabolism in Trichoderma reesei Identifies a Lactose Permease That Is Essential for Cellulase Induction
Source: PLoS One. 2013 May 8;8(5):e62631. doi: 10.1371/journal.pone.0062631 (PMC3648571; doi:10.1371/journal.pone.0062631)
Supplement: Table S5 — Oligonucleotides used in quantitative real-time PCR. (DOCX) [file pone.0062631.s006.docx]

**Table S5**. Oligonucleotides used in quantitative real-time PCR.

| **Gene ID** | **Oligonucleotide** | **Sequence** | **Amplification Efficiency [%]** | **R^2^ value** |
| --- | --- | --- | --- | --- |
| 123902/tef1 | tef1 qPCR fw | 5‘-CCACATTGCCTGCAAGTTCGC-3‘ | 91 | 0,998 |
|  | tef1 qPCR rv | 5‘-GTCGGTGAAAGCCTCAACGCAC-3‘ |  |  |
| 122208/xyr1 | qPCR_xyr1_for | 5‘-CCATCAACCTTCTAGACGAC-3‘ | 105 | 0,997 |
|  | qPCR_xyr1_rev | 5‘-AACCCTGCAGGAGATAGAC-3‘ |  |  |
| 72567/cel6a | cbh2 qPCR fw | 5‘-ACTACAACGGGTGGAACATTAC-3‘ | 106 | 0,999 |
|  | cbh2 qPCR rv | 5‘-CGTGGATGTACAGCTTCTCG-3‘ |  |  |
| 123989/cel7a | cbh1 qPCR fw | 5‘-CCGAGCTTGGTAGTTACTCTG-3‘ | 101 | 0,999 |
|  | cbh1 qPCR rv | 5‘-GGTAGCCTTCTTGAACTGAGT-3‘ |  |  |
| 105260 | ht RT 1 | 5'-AACTTTGGGTGTCCTTTGGC-3' | 98 | 0,999 |
|  | ht RT 2 | 5'-CGCAAACGCAGAAGCAAG-3' |  |  |
| 3405 | st RT 1 | 5'-CCGTCTACCGTCTGTTGTGC-3' | 97 | 0,998 |
|  | st RT 2 | 5'-GAAGTAGGAAAGAACCGCATTG-3' |  |  |
| 79202 | tp RT 1 | 5'-AGGCACGGCAATCTTTCG-3' | 99 | 0,999 |
|  | tp RT 2 | 5'-CGCTCGCCAAACCTATGTG-3' |  |  |
| 56289 | pmfs1 RT 1 | 5'-TATTTCATTGCCTTTGCTACCC-3' | 95 | 0,997 |
|  | pmfs1 RT 2 | 5'-CTCGCCTTCTATTCTCCCTTG-3' |  |  |
| 104072 | pt1 RT 1 | 5'-GCGGAGTCAAAGGAGATCG-3' | 100 | 0,998 |
|  | pt1 RT 2 | 5'-CATTACCACCGAGGAGTTGC-3' |  |  |
| 50894 | pmfs4 RT 1 | 5'-CAGTGGACAGGAATCAATGCC-3' | 104 | 0,999 |
|  | pmfs4 RT 2 | 5'-CCAGTAGCGAGCAGGGAAAC-3' |  |  |
| 56684 | pt2 RT 1 | 5'-TTGGGCATGGGAGCTGTAC-3' | 102 | 0,998 |
|  | pt2 RT 2 | 5'-AGAAGCAAGCCTGGTAAGCAC-3' |  |  |
| 70349 | pmfs5 RT 1 | 5'-CGGCTTCTTCTTCTTCCCAG-3' | 99 | 0,999 |
|  | pmfs5 RT 2 | 5'-CGACAGGCACACGAATAACAG-3' |  |  |
| 69957 | pt4 RT 1 | 5'-TGCCGTGCTACTCGTTGGTC-3' | 96 | 0,998 |
|  | pt4 RT 2 | 5'-GGTTCGTTGGACAGACTTCGG-3' |  |  |
| 121608 | pmfs6 RT 1 | 5'-GGCTTTCCTCCCGTTTGAC-3' | 101 | 0,999 |
|  | pmfs6 RT 2 | 5'-ATCCACAACAGCGTCACCG-3' |  |  |
